# Supplementary material for: The Effect of Aspergillus flavus on Seedling Development in Maize
Source: Plants (Basel). 2025 Apr 2;14(7):1109. doi: 10.3390/plants14071109 (PMC11991196; doi:10.3390/plants14071109)
Supplement: Supplementary file 1 [file plants-14-01109-s001.zip › plants-3490317-supplementary.pdf]

**Table S1.** Phenotypic measures for the tested 92 inbred lines across years. Each inbred line is identified as specific LINE_CODE. The score frequencies (from 1 to 5) of germination inbred line are labeled as SCORE_C in the control and SCORE_A after inoculation with *A. flavus*. The length of coleoptile (cm) is identified as PLANT_C in the control and PLANT_A in the inoculated lines.

| YEAR | LINE_CODE | REPLICATION | SCORE_C | SCORE_A | PLANT_C | PLANT_A |
|---|---|---|---|---|---|---|
| 2001 | 1 | 1 | 1 | 5 | 3.10 | 0.00 |
| 2001 | 1 | 1 | 1 | 5 | 3.70 | 0.00 |
| 2001 | 1 | 1 | 1 | 5 | 3.90 | 0.00 |
| 2001 | 1 | 1 | 1 | 5 | 1.50 | 0.00 |
| 2001 | 1 | 1 | 2 | 5 | 7.50 | 0.00 |
| 2001 | 1 | 1 | 2 | 5 | 3.60 | 0.00 |
| 2001 | 1 | 1 | 2 | 5 | 0.50 | 0.00 |
| 2001 | 1 | 1 | 5 | 5 | 0.00 | 0.00 |
| 2001 | 1 | 1 | 5 | 2 | 0.00 | 4.70 |
| 2001 | 1 | 1 | 5 | 2 | 0.00 | 4.20 |
| 2001 | 1 | 2 | 5 | 5 | 0.00 | 0.00 |
| 2001 | 1 | 2 | 5 | 5 | 0.00 | 0.00 |
| 2001 | 1 | 2 | 5 | 5 | 0.00 | 0.00 |
| 2001 | 1 | 2 | 5 | 5 | 0.00 | 0.00 |
| 2001 | 1 | 2 | 5 | 1 | 0.00 | 5.50 |
| 2001 | 1 | 2 | 1 | 2 | 5.50 | 4.70 |
| 2001 | 1 | 2 | 1 | 2 | 6.00 | 3.00 |
| 2001 | 1 | 2 | 3 | 2 | 3.30 | 2.60 |
| 2001 | 1 | 2 | 2 | 2 | 2.00 | 2.10 |
| 2001 | 1 | 2 | 2 | 2 | 1.50 | 3.60 |
| 2001 | 2 | 1 | 5 | 5 | 0.00 | 0.00 |
| 2001 | 2 | 1 | 3 | 2 | 3.30 | 2.60 |
| 2001 | 2 | 1 | 1 | 2 | 5.10 | 6.90 |
| 2001 | 2 | 1 | 1 | 2 | 6.40 | 7.20 |
| 2001 | 2 | 1 | 1 | 2 | 0.50 | 0.50 |
| 2001 | 2 | 1 | 1 | 2 | 2.20 | 0.50 |
| 2001 | 2 | 1 | 1 | 2 | 1.90 | 6.20 |
| 2001 | 2 | 1 | 1 | 2 | 2.10 | 3.10 |
| 2001 | 2 | 1 | 1 | 2 | 2.60 | 5.30 |
| 2001 | 2 | 1 | 1 | 2 | 0.50 | 3.60 |
| 2001 | 2 | 2 | 5 | 5 | 0.00 | 0.00 |
| 2001 | 2 | 2 | 5 | 5 | 0.00 | 0.00 |
| 2001 | 2 | 2 | 5 | 2 | 0.00 | 7.30 |
| 2001 | 2 | 2 | 5 | 2 | 0.00 | 9.60 |
| 2001 | 2 | 2 | 5 | 2 | 0.00 | 0.90 |
| 2001 | 2 | 2 | 5 | 2 | 0.00 | 2.60 |
| 2001 | 2 | 2 | 1 | 2 | 5.50 | 1.50 |
| 2001 | 2 | 2 | 1 | 2 | 2.50 | 6.80 |
| 2001 | 2 | 2 | 2 | 2 | 0.50 | 5.40 |
| 2001 | 2 | 2 | 2 | 2 | 0.50 | 3.20 |
| 2001 | 3 | 1 | 5 | 5 | 0.00 | 0.00 |
| 2001 | 3 | 1 | 5 | 5 | 0.00 | 0.00 |
| 2001 | 3 | 1 | 5 | 5 | 0.00 | 0.00 |
| 2001 | 3 | 1 | 5 | 5 | 0.00 | 0.00 |
| 2001 | 3 | 1 | 5 | 5 | 0.00 | 0.00 |
| 2001 | 3 | 1 | 5 | 5 | 0.00 | 0.00 |
| 2001 | 3 | 1 | 5 | 4 | 0.00 | 0.50 |
| 2001 | 3 | 1 | 3 | 4 | 0.50 | 0.50 |
| 2001 | 3 | 1 | 4 | 4 | 3.90 | 3.50 |
| 2001 | 3 | 1 | 4 | 4 | 0.90 | 0.50 |

| | | | | | | |
|---|---|---|---|---|---|---|
| 2001 | 3 | 2 | 5 | 2 | 0.00 | 5.60 |
| 2001 | 3 | 2 | 5 | 2 | 0.00 | 3.60 |
| 2001 | 3 | 2 | 5 | 2 | 0.00 | 2.40 |
| 2001 | 3 | 2 | 5 | 2 | 0.00 | 3.00 |
| 2001 | 3 | 2 | 3 | 2 | 2.90 | 4.60 |
| 2001 | 3 | 2 | 3 | 2 | 2.80 | 2.80 |
| 2001 | 3 | 2 | 3 | 2 | 3.50 | 0.50 |
| 2001 | 3 | 2 | 3 | 5 | 1.50 | 0.00 |
| 2001 | 3 | 2 | 3 | 5 | 2.00 | 0.00 |
| 2001 | 3 | 2 | 3 | 5 | 2.20 | 0.00 |
| 2001 | 4 | 1 | 5 | 5 | 0.00 | 0.00 |
| 2001 | 4 | 1 | 5 | 5 | 0.00 | 0.00 |
| 2001 | 4 | 1 | 5 | 5 | 0.00 | 0.00 |
| 2001 | 4 | 1 | 5 | 5 | 0.00 | 0.00 |
| 2001 | 4 | 1 | 5 | 5 | 0.00 | 0.00 |
| 2001 | 4 | 1 | 3 | 5 | 2.90 | 0.00 |
| 2001 | 4 | 1 | 2 | 2 | 4.70 | 4.00 |
| 2001 | 4 | 1 | 2 | 2 | 4.60 | 4.00 |
| 2001 | 4 | 1 | 1 | 2 | 8.40 | 4.20 |
| 2001 | 4 | 1 | 1 | 3 | 4.90 | 4.50 |
| 2001 | 4 | 2 | 5 | 5 | 0.00 | 0.00 |
| 2001 | 4 | 2 | 5 | 5 | 0.00 | 0.00 |
| 2001 | 4 | 2 | 5 | 5 | 0.00 | 0.00 |
| 2001 | 4 | 2 | 5 | 5 | 0.00 | 0.00 |
| 2001 | 4 | 2 | 5 | 5 | 0.00 | 0.00 |
| 2001 | 4 | 2 | 3 | 2 | 4.20 | 5.30 |
| 2001 | 4 | 2 | 3 | 2 | 5.70 | 6.50 |
| 2001 | 4 | 2 | 3 | 2 | 6.50 | 2.60 |
| 2001 | 4 | 2 | 1 | 2 | 14.20 | 4.90 |
| 2001 | 4 | 2 | 2 | 2 | 4.60 | 2.80 |
| 2001 | 5 | 1 | 3 | 2 | 1.90 | 2.80 |
| 2001 | 5 | 1 | 3 | 2 | 4.60 | 4.70 |
| 2001 | 5 | 1 | 2 | 2 | 5.00 | 7.00 |
| 2001 | 5 | 1 | 1 | 2 | 6.40 | 4.90 |
| 2001 | 5 | 1 | 1 | 2 | 7.80 | 4.60 |
| 2001 | 5 | 1 | 1 | 2 | 6.90 | 4.80 |
| 2001 | 5 | 1 | 1 | 2 | 4.90 | 5.00 |
| 2001 | 5 | 1 | 1 | 2 | 4.60 | 0.50 |
| 2001 | 5 | 1 | 1 | 1 | 4.70 | 3.10 |
| 2001 | 5 | 1 | 1 | 1 | 4.50 | 2.10 |
| 2001 | 5 | 2 | 5 | 5 | 0.00 | 0.00 |
| 2001 | 5 | 2 | 5 | 2 | 0.00 | 9.80 |
| 2001 | 5 | 2 | 3 | 2 | 3.60 | 7.40 |
| 2001 | 5 | 2 | 2 | 2 | 0.70 | 3.50 |
| 2001 | 5 | 2 | 1 | 2 | 5.10 | 6.40 |
| 2001 | 5 | 2 | 1 | 2 | 9.30 | 5.90 |
| 2001 | 5 | 2 | 1 | 2 | 7.90 | 7.90 |
| 2001 | 5 | 2 | 1 | 2 | 10.60 | 4.50 |
| 2001 | 5 | 2 | 1 | 2 | 7.60 | 3.70 |
| 2001 | 5 | 2 | 2 | 2 | 6.80 | 3.60 |
| 2001 | 6 | 1 | 1 | 5 | 14.30 | 0.00 |
| 2001 | 6 | 1 | 1 | 5 | 5.00 | 0.00 |
| 2001 | 6 | 1 | 1 | 5 | 8.50 | 0.00 |
| 2001 | 6 | 1 | 1 | 5 | 8.30 | 0.00 |
| 2001 | 6 | 1 | 1 | 5 | 8.00 | 0.00 |
| 2001 | 6 | 1 | 2 | 5 | 3.00 | 0.00 |
| 2001 | 6 | 1 | 2 | 2 | 4.60 | 1.00 |
| 2001 | 6 | 1 | 1 | 2 | 7.60 | 3.70 |

| | | | | | | |
|---|---|---|---|---|---|---|
| 2001 | 6 | 1 | 2 | 2 | 14.40 | 6.60 |
| 2001 | 6 | 1 | 1 | 2 | 12.30 | 9.50 |
| 2001 | 6 | 2 | 1 | 5 | 2.50 | 0.00 |
| 2001 | 6 | 2 | 1 | 5 | 3.00 | 0.00 |
| 2001 | 6 | 2 | 1 | 5 | 4.40 | 0.00 |
| 2001 | 6 | 2 | 1 | 5 | 5.00 | 0.00 |
| 2001 | 6 | 2 | 1 | 5 | 11.50 | 0.00 |
| 2001 | 6 | 2 | 1 | 5 | 18.00 | 0.00 |
| 2001 | 6 | 2 | 1 | 5 | 12.50 | 0.00 |
| 2001 | 6 | 2 | 1 | 5 | 5.40 | 0.00 |
| 2001 | 6 | 2 | 5 | 2 | 0.00 | 7.00 |
| 2001 | 6 | 2 | 5 | 2 | 0.00 | 10.00 |
| 2001 | 7 | 1 | 5 | 2 | 0.00 | 6.20 |
| 2001 | 7 | 1 | 5 | 2 | 0.00 | 6.00 |
| 2001 | 7 | 1 | 4 | 2 | 1.80 | 4.80 |
| 2001 | 7 | 1 | 1 | 2 | 0.90 | 4.30 |
| 2001 | 7 | 1 | 1 | 2 | 3.20 | 4.40 |
| 2001 | 7 | 1 | 1 | 5 | 5.20 | 0.00 |
| 2001 | 7 | 1 | 1 | 5 | 3.10 | 0.00 |
| 2001 | 7 | 1 | 1 | 5 | 3.20 | 0.00 |
| 2001 | 7 | 1 | 1 | 5 | 1.80 | 0.00 |
| 2001 | 7 | 1 | 1 | 5 | 3.90 | 0.00 |
| 2001 | 7 | 2 | 5 | 5 | 0.00 | 0.00 |
| 2001 | 7 | 2 | 5 | 5 | 0.00 | 0.00 |
| 2001 | 7 | 2 | 5 | 5 | 0.00 | 0.00 |
| 2001 | 7 | 2 | 5 | 5 | 0.00 | 0.00 |
| 2001 | 7 | 2 | 5 | 5 | 0.00 | 0.00 |
| 2001 | 7 | 2 | 3 | 5 | 3.00 | 0.00 |
| 2001 | 7 | 2 | 3 | 2 | 2.30 | 3.50 |
| 2001 | 7 | 2 | 3 | 2 | 2.50 | 2.00 |
| 2001 | 7 | 2 | 4 | 2 | 0.10 | 5.00 |
| 2001 | 7 | 2 | 4 | 2 | 0.10 | 5.00 |
| 2001 | 8 | 1 | 5 | 5 | 0.00 | 0.00 |
| 2001 | 8 | 1 | 5 | 5 | 0.00 | 0.00 |
| 2001 | 8 | 1 | 2 | 5 | 9.30 | 0.00 |
| 2001 | 8 | 1 | 2 | 5 | 5.60 | 0.00 |
| 2001 | 8 | 1 | 2 | 5 | 3.90 | 0.00 |
| 2001 | 8 | 1 | 3 | 2 | 3.50 | 2.90 |
| 2001 | 8 | 1 | 2 | 2 | 2.50 | 2.50 |
| 2001 | 8 | 1 | 3 | 2 | 5.50 | 3.80 |
| 2001 | 8 | 1 | 3 | 2 | 9.30 | 0.50 |
| 2001 | 8 | 1 | 1 | 2 | 7.50 | 0.50 |
| 2001 | 8 | 2 | 5 | 5 | 0.00 | 0.00 |
| 2001 | 8 | 2 | 5 | 5 | 0.00 | 0.00 |
| 2001 | 8 | 2 | 5 | 4 | 0.00 | 3.70 |
| 2001 | 8 | 2 | 3 | 4 | 2.90 | 0.50 |
| 2001 | 8 | 2 | 3 | 3 | 3.90 | 6.70 |
| 2001 | 8 | 2 | 3 | 2 | 3.00 | 3.30 |
| 2001 | 8 | 2 | 3 | 2 | 2.50 | 6.60 |
| 2001 | 8 | 2 | 1 | 2 | 3.80 | 5.40 |
| 2001 | 8 | 2 | 1 | 5 | 3.60 | 0.00 |
| 2001 | 8 | 2 | 2 | 5 | 3.10 | 0.00 |
| 2001 | 9 | 1 | 5 | 5 | 0.00 | 0.00 |
| 2001 | 9 | 1 | 5 | 5 | 0.00 | 0.00 |
| 2001 | 9 | 1 | 2 | 5 | 10.90 | 0.00 |
| 2001 | 9 | 1 | 2 | 5 | 7.40 | 0.00 |
| 2001 | 9 | 1 | 2 | 5 | 10.90 | 0.00 |
| 2001 | 9 | 1 | 2 | 5 | 6.70 | 0.00 |

| | | | | | | |
|---|---|---|---|---|---|---|
| 2001 | 9 | 1 | 2 | 5 | 3.50 | 0.00 |
| 2001 | 9 | 1 | 2 | 3 | 1.50 | 5.00 |
| 2001 | 9 | 1 | 2 | 3 | 9.50 | 8.50 |
| 2001 | 9 | 1 | 2 | 2 | 5.40 | 0.00 |
| 2001 | 9 | 2 | 5 | 5 | 0.00 | 0.00 |
| 2001 | 9 | 2 | 2 | 5 | 5.60 | 0.00 |
| 2001 | 9 | 2 | 2 | 2 | 8.50 | 11.20 |
| 2001 | 9 | 2 | 2 | 2 | 14.00 | 14.40 |
| 2001 | 9 | 2 | 2 | 2 | 2.50 | 8.90 |
| 2001 | 9 | 2 | 2 | 2 | 5.50 | 2.00 |
| 2001 | 9 | 2 | 2 | 2 | 5.00 | 4.20 |
| 2001 | 9 | 2 | 2 | 2 | 3.00 | 3.00 |
| 2001 | 9 | 2 | 2 | 2 | 7.50 | 3.70 |
| 2001 | 9 | 2 | 2 | 2 | 4.00 | 4.50 |
| 2001 | 10 | 1 | 5 | 5 | 0.00 | 0.00 |
| 2001 | 10 | 1 | 5 | 5 | 0.00 | 0.00 |
| 2001 | 10 | 1 | 2 | 5 | 6.20 | 0.00 |
| 2001 | 10 | 1 | 2 | 5 | 4.90 | 0.00 |
| 2001 | 10 | 1 | 2 | 5 | 7.30 | 0.00 |
| 2001 | 10 | 1 | 2 | 5 | 7.20 | 0.00 |
| 2001 | 10 | 1 | 2 | 2 | 1.60 | 2.60 |
| 2001 | 10 | 1 | 2 | 2 | 2.30 | 5.50 |
| 2001 | 10 | 1 | 2 | 2 | 3.70 | 4.00 |
| 2001 | 10 | 1 | 2 | 2 | 2.90 | 3.60 |
| 2001 | 10 | 2 | 5 | 5 | 0.00 | 0.00 |
| 2001 | 10 | 2 | 3 | 5 | 4.20 | 0.00 |
| 2001 | 10 | 2 | 2 | 5 | 5.60 | 0.00 |
| 2001 | 10 | 2 | 2 | 2 | 4.30 | 2.00 |
| 2001 | 10 | 2 | 2 | 2 | 2.60 | 2.80 |
| 2001 | 10 | 2 | 2 | 2 | 10.80 | 2.70 |
| 2001 | 10 | 2 | 2 | 2 | 0.50 | 1.80 |
| 2001 | 10 | 2 | 2 | 2 | 2.30 | 1.80 |
| 2001 | 10 | 2 | 2 | 2 | 10.50 | 1.50 |
| 2001 | 10 | 2 | 2 | 3 | 3.70 | 3.00 |
| 2001 | 11 | 1 | 5 | 5 | 0.00 | 0.00 |
| 2001 | 11 | 1 | 3 | 3 | 3.90 | 11.00 |
| 2001 | 11 | 1 | 2 | 3 | 4.40 | 3.00 |
| 2001 | 11 | 1 | 2 | 2 | 5.70 | 10.00 |
| 2001 | 11 | 1 | 2 | 2 | 5.80 | 5.40 |
| 2001 | 11 | 1 | 2 | 2 | 7.90 | 7.30 |
| 2001 | 11 | 1 | 2 | 2 | 6.50 | 3.00 |
| 2001 | 11 | 1 | 2 | 2 | 7.60 | 10.00 |
| 2001 | 11 | 1 | 2 | 2 | 3.70 | 7.80 |
| 2001 | 11 | 1 | 2 | 2 | 12.90 | 10.30 |
| 2001 | 11 | 2 | 5 | 5 | 0.00 | 0.00 |
| 2001 | 11 | 2 | 2 | 5 | 5.20 | 0.00 |
| 2001 | 11 | 2 | 2 | 5 | 11.10 | 0.00 |
| 2001 | 11 | 2 | 2 | 5 | 14.50 | 0.00 |
| 2001 | 11 | 2 | 2 | 2 | 13.80 | 4.80 |
| 2001 | 11 | 2 | 2 | 2 | 7.60 | 4.00 |
| 2001 | 11 | 2 | 2 | 2 | 14.30 | 7.80 |
| 2001 | 11 | 2 | 2 | 2 | 5.70 | 9.80 |
| 2001 | 11 | 2 | 2 | 2 | 4.20 | 6.40 |
| 2001 | 11 | 2 | 2 | 2 | 6.80 | 6.50 |
| 2001 | 12 | 1 | 5 | 5 | 0.00 | 0.00 |
| 2001 | 12 | 1 | 5 | 5 | 0.00 | 0.00 |
| 2001 | 12 | 1 | 2 | 5 | 9.80 | 0.00 |
| 2001 | 12 | 1 | 2 | 5 | 10.60 | 0.00 |

| | | | | | | |
|---|---|---|---|---|---|---|
| 2001 | 12 | 1 | 2 | 2 | 2.70 | 5.00 |
| 2001 | 12 | 1 | 2 | 2 | 13.40 | 8.30 |
| 2001 | 12 | 1 | 2 | 2 | 8.20 | 7.00 |
| 2001 | 12 | 1 | 2 | 2 | 13.00 | 11.00 |
| 2001 | 12 | 1 | 2 | 2 | 4.80 | 4.90 |
| 2001 | 12 | 1 | 2 | 2 | 5.90 | 7.80 |
| 2001 | 12 | 2 | 3 | 5 | 7.40 | 0.00 |
| 2001 | 12 | 2 | 3 | 5 | 4.20 | 0.00 |
| 2001 | 12 | 2 | 2 | 2 | 6.30 | 2.40 |
| 2001 | 12 | 2 | 2 | 2 | 3.90 | 1.50 |
| 2001 | 12 | 2 | 2 | 2 | 2.60 | 1.50 |
| 2001 | 12 | 2 | 2 | 2 | 4.20 | 5.50 |
| 2001 | 12 | 2 | 2 | 2 | 1.20 | 1.50 |
| 2001 | 12 | 2 | 2 | 2 | 2.70 | 2.50 |
| 2001 | 12 | 2 | 2 | 2 | 4.40 | 2.50 |
| 2001 | 12 | 2 | 2 | 2 | 1.60 | 4.00 |
| 2001 | 13 | 1 | 5 | 5 | 0.00 | 0.00 |
| 2001 | 13 | 1 | 2 | 5 | 13.20 | 0.00 |
| 2001 | 13 | 1 | 2 | 5 | 10.50 | 0.00 |
| 2001 | 13 | 1 | 2 | 2 | 7.50 | 13.50 |
| 2001 | 13 | 1 | 2 | 2 | 3.60 | 11.70 |
| 2001 | 13 | 1 | 2 | 2 | 9.30 | 8.20 |
| 2001 | 13 | 1 | 2 | 2 | 10.20 | 10.30 |
| 2001 | 13 | 1 | 2 | 2 | 15.40 | 8.40 |
| 2001 | 13 | 1 | 2 | 2 | 14.70 | 8.50 |
| 2001 | 13 | 1 | 2 | 2 | 0.00 | 11.20 |
| 2001 | 13 | 2 | 5 | 5 | 0.00 | 0.00 |
| 2001 | 13 | 2 | 2 | 2 | 6.80 | 4.00 |
| 2001 | 13 | 2 | 2 | 2 | 11.70 | 4.40 |
| 2001 | 13 | 2 | 2 | 2 | 3.90 | 5.00 |
| 2001 | 13 | 2 | 2 | 2 | 2.70 | 6.00 |
| 2001 | 13 | 2 | 2 | 2 | 12.30 | 6.20 |
| 2001 | 13 | 2 | 2 | 2 | 4.30 | 3.80 |
| 2001 | 13 | 2 | 2 | 2 | 5.90 | 4.30 |
| 2001 | 13 | 2 | 2 | 2 | 6.70 | 3.60 |
| 2001 | 13 | 2 | 2 | 2 | 1.80 | 3.50 |
| 2001 | 14 | 1 | 5 | 5 | 0.00 | 0.00 |
| 2001 | 14 | 1 | 5 | 3 | 0.00 | 3.20 |
| 2001 | 14 | 1 | 5 | 3 | 0.00 | 4.10 |
| 2001 | 14 | 1 | 3 | 3 | 3.20 | 32.00 |
| 2001 | 14 | 1 | 2 | 3 | 1.20 | 2.30 |
| 2001 | 14 | 1 | 2 | 3 | 1.10 | 2.60 |
| 2001 | 14 | 1 | 2 | 3 | 1.30 | 2.80 |
| 2001 | 14 | 1 | 2 | 3 | 1.50 | 2.80 |
| 2001 | 14 | 1 | 2 | 3 | 0.70 | 3.00 |
| 2001 | 14 | 1 | 2 | 3 | 2.50 | 2.50 |
| 2001 | 14 | 2 | 5 | 5 | 0.00 | 0.00 |
| 2001 | 14 | 2 | 5 | 2 | 0.00 | 2.00 |
| 2001 | 14 | 2 | 2 | 2 | 2.50 | 3.00 |
| 2001 | 14 | 2 | 2 | 2 | 3.00 | 2.40 |
| 2001 | 14 | 2 | 2 | 2 | 2.50 | 2.00 |
| 2001 | 14 | 2 | 2 | 2 | 2.70 | 1.50 |
| 2001 | 14 | 2 | 2 | 2 | 3.20 | 2.50 |
| 2001 | 14 | 2 | 2 | 2 | 3.30 | 2.40 |
| 2001 | 14 | 2 | 2 | 2 | 3.20 | 2.00 |
| 2001 | 14 | 2 | 2 | 2 | 0.50 | 2.50 |
| 2001 | 15 | 1 | 5 | 2 | 0.00 | 4.30 |
| 2001 | 15 | 1 | 5 | 2 | 0.00 | 11.70 |

| | | | | | | |
|---|---|---|---|---|---|---|
| 2001 | 15 | 1 | 5 | 2 | 0.00 | 3.70 |
| 2001 | 15 | 1 | 3 | 2 | 5.40 | 5.30 |
| 2001 | 15 | 1 | 2 | 3 | 6.30 | 3.80 |
| 2001 | 15 | 1 | 2 | 5 | 7.50 | 0.00 |
| 2001 | 15 | 1 | 2 | 5 | 7.80 | 0.00 |
| 2001 | 15 | 1 | 2 | 5 | 4.20 | 0.00 |
| 2001 | 15 | 1 | 2 | 2 | 1.50 | 0.00 |
| 2001 | 15 | 1 | 5 | 5 | 0.00 | 0.00 |
| 2001 | 15 | 2 | 5 | 5 | 0.00 | 0.00 |
| 2001 | 15 | 2 | 5 | 5 | 0.00 | 0.00 |
| 2001 | 15 | 2 | 2 | 5 | 6.20 | 0.00 |
| 2001 | 15 | 2 | 2 | 2 | 4.30 | 2.00 |
| 2001 | 15 | 2 | 2 | 2 | 9.00 | 3.80 |
| 2001 | 15 | 2 | 2 | 2 | 6.50 | 2.50 |
| 2001 | 15 | 2 | 2 | 2 | 5.20 | 4.50 |
| 2001 | 15 | 2 | 2 | 2 | 2.50 | 0.00 |
| 2001 | 15 | 2 | 2 | 2 | 3.60 | 0.00 |
| 2001 | 15 | 2 | 2 | 2 | 0.80 | 0.00 |
| 2001 | 16 | 1 | 5 | 5 | 0.00 | 0.00 |
| 2001 | 16 | 1 | 5 | 5 | 0.00 | 0.00 |
| 2001 | 16 | 1 | 2 | 5 | 2.10 | 0.00 |
| 2001 | 16 | 1 | 2 | 5 | 0.70 | 0.00 |
| 2001 | 16 | 1 | 2 | 5 | 2.60 | 0.00 |
| 2001 | 16 | 1 | 2 | 2 | 2.00 | 4.40 |
| 2001 | 16 | 1 | 2 | 2 | 0.40 | 4.60 |
| 2001 | 16 | 1 | 2 | 2 | 0.30 | 1.00 |
| 2001 | 16 | 1 | 2 | 2 | 2.60 | 1.50 |
| 2001 | 16 | 1 | 2 | 5 | 1.80 | 0.00 |
| 2001 | 16 | 2 | 5 | 5 | 0.00 | 0.00 |
| 2001 | 16 | 2 | 5 | 5 | 0.00 | 0.00 |
| 2001 | 16 | 2 | 5 | 5 | 0.00 | 0.00 |
| 2001 | 16 | 2 | 5 | 5 | 0.00 | 0.00 |
| 2001 | 16 | 2 | 5 | 5 | 0.00 | 0.00 |
| 2001 | 16 | 2 | 2 | 5 | 2.60 | 0.00 |
| 2001 | 16 | 2 | 2 | 5 | 1.00 | 0.00 |
| 2001 | 16 | 2 | 2 | 5 | 2.80 | 0.00 |
| 2001 | 16 | 2 | 2 | 5 | 1.50 | 0.00 |
| 2001 | 16 | 2 | 2 | 2 | 0.50 | 1.80 |
| 2001 | 17 | 1 | 3 | 2 | 5.60 | 13.30 |
| 2001 | 17 | 1 | 2 | 2 | 4.90 | 8.40 |
| 2001 | 17 | 1 | 2 | 2 | 9.20 | 8.00 |
| 2001 | 17 | 1 | 2 | 2 | 11.50 | 10.60 |
| 2001 | 17 | 1 | 2 | 2 | 7.40 | 16.30 |
| 2001 | 17 | 1 | 2 | 2 | 16.80 | 11.00 |
| 2001 | 17 | 1 | 2 | 2 | 14.90 | 4.00 |
| 2001 | 17 | 1 | 2 | 2 | 13.00 | 0.00 |
| 2001 | 17 | 1 | 2 | 3 | 10.20 | 10.50 |
| 2001 | 17 | 1 | 2 | 3 | 11.40 | 16.40 |
| 2001 | 17 | 2 | 2 | 5 | 11.50 | 0.00 |
| 2001 | 17 | 2 | 2 | 5 | 11.60 | 0.00 |
| 2001 | 17 | 2 | 2 | 2 | 14.00 | 6.80 |
| 2001 | 17 | 2 | 2 | 2 | 18.50 | 7.90 |
| 2001 | 17 | 2 | 2 | 2 | 11.00 | 10.30 |
| 2001 | 17 | 2 | 2 | 2 | 19.00 | 7.40 |
| 2001 | 17 | 2 | 2 | 2 | 15.00 | 11.30 |
| 2001 | 17 | 2 | 2 | 2 | 17.50 | 11.90 |
| 2001 | 17 | 2 | 2 | 2 | 19.60 | 9.70 |
| 2001 | 17 | 2 | 2 | 2 | 12.00 | 8.00 |

| | | | | | | |
|---|---|---|---|---|---|---|
| 2001 | 18 | 1 | 5 | 5 | 0.00 | 0.00 |
| 2001 | 18 | 1 | 5 | 5 | 0.00 | 0.00 |
| 2001 | 18 | 1 | 5 | 5 | 0.00 | 0.00 |
| 2001 | 18 | 1 | 5 | 5 | 0.00 | 0.00 |
| 2001 | 18 | 1 | 5 | 5 | 0.00 | 0.00 |
| 2001 | 18 | 1 | 5 | 5 | 0.00 | 0.00 |
| 2001 | 18 | 1 | 2 | 5 | 5.90 | 0.00 |
| 2001 | 18 | 1 | 2 | 5 | 5.30 | 0.00 |
| 2001 | 18 | 1 | 2 | 5 | 3.10 | 0.00 |
| 2001 | 18 | 1 | 2 | 5 | 3.20 | 0.00 |
| 2001 | 18 | 2 | 5 | 5 | 0.00 | 0.00 |
| 2001 | 18 | 2 | 5 | 5 | 0.00 | 0.00 |
| 2001 | 18 | 2 | 5 | 5 | 0.00 | 0.00 |
| 2001 | 18 | 2 | 5 | 5 | 0.00 | 0.00 |
| 2001 | 18 | 2 | 5 | 5 | 0.00 | 0.00 |
| 2001 | 18 | 2 | 5 | 5 | 0.00 | 0.00 |
| 2001 | 18 | 2 | 5 | 5 | 0.00 | 0.00 |
| 2001 | 18 | 2 | 2 | 5 | 3.70 | 0.00 |
| 2001 | 18 | 2 | 2 | 2 | 3.80 | 6.10 |
| 2001 | 18 | 2 | 1 | 4 | 10.00 | 6.20 |
| 2001 | 19 | 1 | 5 | 5 | 0.00 | 0.00 |
| 2001 | 19 | 1 | 5 | 5 | 0.00 | 0.00 |
| 2001 | 19 | 1 | 5 | 5 | 0.00 | 0.00 |
| 2001 | 19 | 1 | 5 | 5 | 0.00 | 0.00 |
| 2001 | 19 | 1 | 5 | 5 | 0.00 | 0.00 |
| 2001 | 19 | 1 | 5 | 5 | 0.00 | 0.00 |
| 2001 | 19 | 1 | 5 | 5 | 0.00 | 0.00 |
| 2001 | 19 | 1 | 5 | 5 | 0.00 | 0.00 |
| 2001 | 19 | 1 | 5 | 5 | 0.00 | 0.00 |
| 2001 | 19 | 1 | 2 | 5 | 3.40 | 0.00 |
| 2001 | 19 | 2 | 5 | 5 | 0.00 | 0.00 |
| 2001 | 19 | 2 | 5 | 5 | 0.00 | 0.00 |
| 2001 | 19 | 2 | 5 | 5 | 0.00 | 0.00 |
| 2001 | 19 | 2 | 5 | 5 | 0.00 | 0.00 |
| 2001 | 19 | 2 | 5 | 5 | 0.00 | 0.00 |
| 2001 | 19 | 2 | 5 | 5 | 0.00 | 0.00 |
| 2001 | 19 | 2 | 5 | 5 | 0.00 | 0.00 |
| 2001 | 19 | 2 | 5 | 5 | 0.00 | 0.00 |
| 2001 | 19 | 2 | 5 | 5 | 0.00 | 0.00 |
| 2001 | 19 | 2 | 5 | 5 | 0.00 | 0.00 |
| 2010 | 20 | 1 | 1 | 2 | 7.50 | 1.90 |
| 2010 | 20 | 1 | 1 | 2 | 6.60 | 4.10 |
| 2010 | 20 | 1 | 1 | 2 | 11.40 | 6.80 |
| 2010 | 20 | 1 | 1 | 2 | 9.20 | 5.90 |
| 2010 | 20 | 1 | 1 | 2 | 3.60 | 9.40 |
| 2010 | 20 | 1 | 1 | 2 | 5.30 | 9.80 |
| 2010 | 20 | 1 | 1 | 2 | 4.20 | 1.80 |
| 2010 | 20 | 1 | 1 | 2 | 7.30 | 7.40 |
| 2010 | 20 | 1 | 1 | 2 | 2.50 | 6.80 |
| 2010 | 20 | 2 | 1 | 2 | 0.50 | 7.30 |
| 2010 | 20 | 2 | 1 | 2 | 2.80 | 0.50 |
| 2010 | 20 | 2 | 1 | 2 | 9.00 | 2.40 |
| 2010 | 20 | 2 | 1 | 2 | 10.50 | 8.40 |
| 2010 | 20 | 2 | 1 | 2 | 11.80 | 5.10 |
| 2010 | 20 | 2 | 1 | 2 | 9.90 | 2.30 |
| 2010 | 20 | 2 | 1 | 2 | 10.90 | 7.30 |
| 2010 | 20 | 2 | 1 | 2 | 10.40 | 6.40 |
| 2010 | 20 | 2 | 1 | 2 | 6.40 | 6.10 |

| | | | | | | |
|---|---|---|---|---|---|---|
| 2010 | 20 | 2 | 1 | 2 | 5.60 | 2.50 |
| 2010 | 20 | 1 | 1 | 2 | 3.50 | 3.40 |
| 2010 | 21 | 1 | 1 | 4 | 4.80 | 4.00 |
| 2010 | 21 | 1 | 1 | 2 | 5.10 | 7.30 |
| 2010 | 21 | 1 | 1 | 2 | 6.10 | 1.50 |
| 2010 | 21 | 1 | 1 | 2 | 4.60 | 8.80 |
| 2010 | 21 | 1 | 1 | 2 | 6.90 | 6.50 |
| 2010 | 21 | 1 | 1 | 2 | 5.60 | 7.00 |
| 2010 | 21 | 1 | 1 | 2 | 7.50 | 5.60 |
| 2010 | 21 | 1 | 1 | 2 | 6.20 | 5.90 |
| 2010 | 21 | 1 | 1 | 2 | 10.00 | 6.10 |
| 2010 | 21 | 1 | 1 | 2 | 5.50 | 4.50 |
| 2010 | 21 | 2 | 1 | 2 | 10.20 | 5.20 |
| 2010 | 21 | 2 | 1 | 1 | 11.50 | 6.60 |
| 2010 | 21 | 2 | 1 | 1 | 4.00 | 6.70 |
| 2010 | 21 | 2 | 1 | 1 | 5.60 | 2.60 |
| 2010 | 21 | 2 | 3 | 1 | 4.60 | 8.10 |
| 2010 | 21 | 2 | 1 | 1 | 9.70 | 1.90 |
| 2010 | 21 | 2 | 1 | 1 | 8.50 | 1.80 |
| 2010 | 21 | 2 | 1 | 1 | 8.90 | 4.30 |
| 2010 | 21 | 2 | 1 | 1 | 10.40 | 6.50 |
| 2010 | 21 | 2 | 1 | 1 | 8.70 | 5.80 |
| 2010 | 22 | 1 | 1 | 5 | 4.20 | 0.00 |
| 2010 | 22 | 1 | 1 | 5 | 0.50 | 0.00 |
| 2010 | 22 | 1 | 1 | 5 | 2.20 | 0.00 |
| 2010 | 22 | 1 | 1 | 5 | 3.20 | 0.00 |
| 2010 | 22 | 1 | 1 | 5 | 3.50 | 0.00 |
| 2010 | 22 | 1 | 1 | 5 | 2.50 | 0.00 |
| 2010 | 22 | 1 | 1 | 4 | 1.50 | 4.30 |
| 2010 | 22 | 1 | 1 | 4 | 4.50 | 4.60 |
| 2010 | 22 | 1 | 1 | 2 | 2.50 | 2.80 |
| 2010 | 22 | 1 | 1 | 2 | 1.20 | 3.40 |
| 2010 | 22 | 2 | 1 | 5 | 3.60 | 0.00 |
| 2010 | 22 | 2 | 1 | 5 | 8.10 | 0.00 |
| 2010 | 22 | 2 | 1 | 2 | 8.90 | 0.50 |
| 2010 | 22 | 2 | 1 | 2 | 8.40 | 0.50 |
| 2010 | 22 | 2 | 1 | 2 | 9.60 | 0.50 |
| 2010 | 22 | 2 | 1 | 2 | 3.20 | 0.50 |
| 2010 | 22 | 2 | 1 | 2 | 11.50 | 0.50 |
| 2010 | 22 | 2 | 1 | 2 | 6.90 | 0.40 |
| 2010 | 22 | 2 | 1 | 2 | 9.30 | 1.10 |
| 2010 | 22 | 2 | 1 | 4 | 11.40 | 3.10 |
| 2010 | 23 | 1 | 5 | 2 | 0.00 | 6.30 |
| 2010 | 23 | 1 | 5 | 2 | 0.00 | 5.20 |
| 2010 | 23 | 1 | 5 | 2 | 0.00 | 6.00 |
| 2010 | 23 | 1 | 1 | 2 | 3.50 | 7.60 |
| 2010 | 23 | 1 | 1 | 2 | 2.90 | 3.40 |
| 2010 | 23 | 1 | 1 | 2 | 5.20 | 4.30 |
| 2010 | 23 | 1 | 1 | 2 | 4.30 | 0.50 |
| 2010 | 23 | 1 | 1 | 2 | 0.50 | 3.20 |
| 2010 | 23 | 1 | 1 | 2 | 5.50 | 5.60 |
| 2010 | 23 | 1 | 1 | 2 | 2.20 | 7.30 |
| 2010 | 23 | 2 | 5 | 2 | 3.60 | 9.40 |
| 2010 | 23 | 2 | 5 | 2 | 1.90 | 3.60 |
| 2010 | 23 | 2 | 5 | 2 | 5.50 | 7.20 |
| 2010 | 23 | 2 | 1 | 2 | 5.80 | 6.50 |
| 2010 | 23 | 2 | 1 | 2 | 8.60 | 4.40 |
| 2010 | 23 | 2 | 1 | 2 | 4.50 | 5.20 |

| | | | | | | |
|---|---|---|---|---|---|---|
| 2010 | 23 | 2 | 1 | 2 | 4.30 | 2.90 |
| 2010 | 23 | 2 | 1 | 2 | 4.40 | 9.20 |
| 2010 | 23 | 2 | 1 | 2 | 4.30 | 4.10 |
| 2010 | 23 | 2 | 1 | 2 | 4.10 | 6.30 |
| 2010 | 24 | 1 | 1 | 2 | 0.50 | 0.50 |
| 2010 | 24 | 1 | 1 | 2 | 1.70 | 6.30 |
| 2010 | 24 | 1 | 1 | 2 | 1.20 | 4.20 |
| 2010 | 24 | 1 | 1 | 3 | 1.90 | 5.10 |
| 2010 | 24 | 1 | 1 | 2 | 1.60 | 4.30 |
| 2010 | 24 | 1 | 1 | 2 | 1.70 | 3.60 |
| 2010 | 24 | 1 | 1 | 2 | 6.50 | 4.90 |
| 2010 | 24 | 1 | 1 | 2 | 5.50 | 4.10 |
| 2010 | 24 | 1 | 1 | 2 | 1.30 | 2.30 |
| 2010 | 24 | 1 | 5 | 2 | 0.00 | 4.20 |
| 2010 | 24 | 2 | 5 | 2 | 0.00 | 7.70 |
| 2010 | 24 | 2 | 1 | 2 | 1.60 | 5.40 |
| 2010 | 24 | 2 | 1 | 2 | 2.00 | 5.60 |
| 2010 | 24 | 2 | 1 | 2 | 3.50 | 4.80 |
| 2010 | 24 | 2 | 1 | 2 | 0.50 | 4.50 |
| 2010 | 24 | 2 | 1 | 2 | 0.50 | 0.50 |
| 2010 | 24 | 2 | 1 | 2 | 2.60 | 0.50 |
| 2010 | 24 | 2 | 1 | 2 | 5.50 | 3.30 |
| 2010 | 24 | 2 | 1 | 2 | 5.30 | 4.20 |
| 2010 | 24 | 2 | 1 | 2 | 0.70 | 5.10 |
| 2010 | 25 | 1 | 1 | 2 | 3.20 | 10.40 |
| 2010 | 25 | 1 | 1 | 2 | 12.50 | 13.30 |
| 2010 | 25 | 1 | 1 | 2 | 13.00 | 11.30 |
| 2010 | 25 | 1 | 1 | 2 | 11.00 | 9.60 |
| 2010 | 25 | 1 | 1 | 2 | 11.50 | 12.20 |
| 2010 | 25 | 1 | 1 | 2 | 10.50 | 14.70 |
| 2010 | 25 | 1 | 1 | 2 | 9.20 | 11.10 |
| 2010 | 25 | 1 | 1 | 2 | 12.50 | 10.50 |
| 2010 | 25 | 1 | 1 | 4 | 10.70 | 6.60 |
| 2010 | 25 | 1 | 5 | 4 | 0.00 | 11.60 |
| 2010 | 25 | 2 | 1 | 2 | 0.50 | 11.20 |
| 2010 | 25 | 2 | 1 | 2 | 9.20 | 12.20 |
| 2010 | 25 | 2 | 1 | 2 | 14.30 | 3.50 |
| 2010 | 25 | 2 | 1 | 2 | 15.80 | 8.50 |
| 2010 | 25 | 2 | 1 | 2 | 12.00 | 6.30 |
| 2010 | 25 | 2 | 1 | 2 | 10.20 | 5.50 |
| 2010 | 25 | 2 | 1 | 2 | 18.50 | 0.90 |
| 2010 | 25 | 2 | 1 | 2 | 18.90 | 5.90 |
| 2010 | 25 | 2 | 1 | 2 | 9.60 | 3.80 |
| 2010 | 25 | 2 | 1 | 5 | 10.80 | 0.00 |
| 2010 | 26 | 1 | 1 | 2 | 1.00 | 0.50 |
| 2010 | 26 | 1 | 1 | 2 | 0.50 | 0.50 |
| 2010 | 26 | 1 | 1 | 2 | 0.50 | 0.50 |
| 2010 | 26 | 1 | 1 | 2 | 1.20 | 0.50 |
| 2010 | 26 | 1 | 1 | 2 | 1.40 | 0.50 |
| 2010 | 26 | 1 | 5 | 2 | 0.00 | 2.30 |
| 2010 | 26 | 1 | 5 | 2 | 0.00 | 1.90 |
| 2010 | 26 | 1 | 5 | 2 | 0.00 | 1.60 |
| 2010 | 26 | 1 | 5 | 2 | 0.00 | 3.20 |
| 2010 | 26 | 1 | 5 | 2 | 0.00 | 4.10 |
| 2010 | 26 | 2 | 1 | 2 | 0.50 | 1.20 |
| 2010 | 26 | 2 | 1 | 5 | 3.20 | 0.00 |
| 2010 | 26 | 2 | 1 | 1 | 3.90 | 0.50 |
| 2010 | 26 | 2 | 1 | 1 | 0.50 | 0.50 |

| | | | | | | |
|---|---|---|---|---|---|---|
| 2010 | 26 | 2 | 1 | 1 | 0.50 | 0.50 |
| 2010 | 26 | 2 | 1 | 1 | 4.20 | 0.50 |
| 2010 | 26 | 2 | 1 | 1 | 0.50 | 0.50 |
| 2010 | 26 | 2 | 1 | 1 | 4.20 | 0.50 |
| 2010 | 26 | 2 | 1 | 1 | 2.70 | 0.50 |
| 2010 | 26 | 2 | 1 | 1 | 1.30 | 0.50 |
| 2010 | 27 | 1 | 1 | 2 | 8.70 | 0.00 |
| 2010 | 27 | 1 | 1 | 2 | 4.50 | 0.00 |
| 2010 | 27 | 1 | 1 | 2 | 5.10 | 0.00 |
| 2010 | 27 | 1 | 1 | 2 | 11.30 | 1.10 |
| 2010 | 27 | 1 | 1 | 2 | 5.20 | 1.50 |
| 2010 | 27 | 1 | 1 | 2 | 3.90 | 1.30 |
| 2010 | 27 | 1 | 1 | 2 | 11.70 | 0.90 |
| 2010 | 27 | 1 | 1 | 2 | 7.70 | 1.60 |
| 2010 | 27 | 1 | 1 | 2 | 10.60 | 2.30 |
| 2010 | 27 | 1 | 1 | 2 | 4.50 | 1.30 |
| 2010 | 27 | 2 | 1 | 4 | 4.30 | 3.10 |
| 2010 | 27 | 2 | 1 | 2 | 4.60 | 4.30 |
| 2010 | 27 | 2 | 1 | 2 | 4.90 | 6.20 |
| 2010 | 27 | 2 | 1 | 2 | 10.30 | 2.00 |
| 2010 | 27 | 2 | 1 | 2 | 5.50 | 3.60 |
| 2010 | 27 | 2 | 1 | 2 | 6.10 | 0.50 |
| 2010 | 27 | 2 | 1 | 2 | 4.40 | 1.10 |
| 2010 | 27 | 2 | 1 | 2 | 8.90 | 0.90 |
| 2010 | 27 | 2 | 1 | 2 | 6.10 | 0.70 |
| 2010 | 27 | 2 | 1 | 2 | 5.30 | 1.40 |
| 2010 | 28 | 1 | 1 | 5 | 6.50 | 0.00 |
| 2010 | 28 | 1 | 1 | 2 | 5.40 | 3.90 |
| 2010 | 28 | 1 | 1 | 2 | 4.70 | 4.20 |
| 2010 | 28 | 1 | 1 | 2 | 4.60 | 3.40 |
| 2010 | 28 | 1 | 1 | 2 | 3.80 | 3.30 |
| 2010 | 28 | 1 | 1 | 2 | 6.10 | 4.20 |
| 2010 | 28 | 1 | 1 | 2 | 1.50 | 4.60 |
| 2010 | 28 | 1 | 1 | 4 | 6.50 | 6.40 |
| 2010 | 28 | 1 | 1 | 4 | 3.40 | 5.80 |
| 2010 | 28 | 1 | 1 | 4 | 5.20 | 2.30 |
| 2010 | 28 | 2 | 1 | 2 | 5.30 | 4.20 |
| 2010 | 28 | 2 | 1 | 2 | 5.90 | 3.90 |
| 2010 | 28 | 2 | 1 | 2 | 0.50 | 4.40 |
| 2010 | 28 | 2 | 1 | 2 | 3.60 | 3.50 |
| 2010 | 28 | 2 | 1 | 2 | 5.80 | 3.80 |
| 2010 | 28 | 2 | 1 | 2 | 8.30 | 1.90 |
| 2010 | 28 | 2 | 1 | 2 | 6.60 | 4.00 |
| 2010 | 28 | 2 | 1 | 2 | 4.20 | 3.00 |
| 2010 | 28 | 2 | 1 | 2 | 3.10 | 4.20 |
| 2010 | 28 | 2 | 1 | 2 | 4.20 | 3.70 |
| 2010 | 29 | 1 | 5 | 5 | 0.00 | 0.00 |
| 2010 | 29 | 1 | 1 | 5 | 2.20 | 0.00 |
| 2010 | 29 | 1 | 1 | 2 | 2.40 | 6.20 |
| 2010 | 29 | 1 | 1 | 2 | 2.20 | 4.30 |
| 2010 | 29 | 1 | 1 | 2 | 2.30 | 2.90 |
| 2010 | 29 | 1 | 1 | 2 | 0.50 | 2.80 |
| 2010 | 29 | 1 | 1 | 2 | 4.50 | 2.80 |
| 2010 | 29 | 1 | 1 | 2 | 0.50 | 11.20 |
| 2010 | 29 | 1 | 1 | 2 | 0.50 | 7.40 |
| 2010 | 29 | 1 | 1 | 2 | 2.80 | 3.70 |
| 2010 | 29 | 2 | 5 | 5 | 0.00 | 0.00 |
| 2010 | 29 | 2 | 1 | 1 | 3.20 | 0.50 |

| | | | | | | |
|---|---|---|---|---|---|---|
| 2010 | 29 | 2 | 1 | 1 | 6.80 | 0.50 |
| 2010 | 29 | 2 | 1 | 2 | 2.70 | 1.50 |
| 2010 | 29 | 2 | 1 | 2 | 5.90 | 0.60 |
| 2010 | 29 | 2 | 1 | 2 | 4.60 | 1.70 |
| 2010 | 29 | 2 | 1 | 2 | 4.70 | 3.40 |
| 2010 | 29 | 2 | 1 | 2 | 4.10 | 0.90 |
| 2010 | 29 | 2 | 1 | 2 | 4.30 | 0.50 |
| 2010 | 29 | 2 | 1 | 2 | 5.30 | 0.40 |
| 2010 | 30 | 1 | 1 | 2 | 3.80 | 2.90 |
| 2010 | 30 | 1 | 1 | 2 | 6.70 | 3.60 |
| 2010 | 30 | 1 | 1 | 2 | 10.50 | 2.70 |
| 2010 | 30 | 1 | 1 | 2 | 10.10 | 0.50 |
| 2010 | 30 | 1 | 1 | 2 | 9.00 | 3.70 |
| 2010 | 30 | 1 | 1 | 2 | 7.90 | 6.90 |
| 2010 | 30 | 1 | 1 | 2 | 9.30 | 5.50 |
| 2010 | 30 | 1 | 1 | 2 | 6.90 | 9.60 |
| 2010 | 30 | 1 | 1 | 2 | 2.50 | 3.70 |
| 2010 | 30 | 1 | 1 | 2 | 5.00 | 4.50 |
| 2010 | 30 | 2 | 1 | 5 | 14.60 | 0.00 |
| 2010 | 30 | 2 | 1 | 2 | 17.20 | 4.40 |
| 2010 | 30 | 2 | 1 | 2 | 14.20 | 6.20 |
| 2010 | 30 | 2 | 1 | 2 | 17.40 | 5.20 |
| 2010 | 30 | 2 | 1 | 2 | 14.40 | 5.30 |
| 2010 | 30 | 2 | 1 | 2 | 14.70 | 5.70 |
| 2010 | 30 | 2 | 1 | 2 | 8.30 | 5.40 |
| 2010 | 30 | 2 | 3 | 2 | 3.00 | 10.10 |
| 2010 | 30 | 2 | 3 | 2 | 9.20 | 5.30 |
| 2010 | 30 | 2 | 1 | 2 | 0.50 | 7.20 |
| 2010 | 31 | 1 | 1 | 2 | 4.70 | 10.00 |
| 2010 | 31 | 1 | 1 | 2 | 10.20 | 6.20 |
| 2010 | 31 | 1 | 1 | 2 | 8.90 | 7.20 |
| 2010 | 31 | 1 | 1 | 2 | 3.50 | 9.50 |
| 2010 | 31 | 1 | 1 | 2 | 9.00 | 9.00 |
| 2010 | 31 | 1 | 1 | 2 | 8.50 | 8.40 |
| 2010 | 31 | 1 | 1 | 2 | 5.90 | 6.00 |
| 2010 | 31 | 1 | 1 | 2 | 7.80 | 2.50 |
| 2010 | 31 | 1 | 1 | 2 | 3.60 | 4.00 |
| 2010 | 31 | 1 | 1 | 2 | 6.00 | 5.00 |
| 2010 | 31 | 2 | 1 | 2 | 12.60 | 7.20 |
| 2010 | 31 | 2 | 1 | 2 | 12.70 | 6.10 |
| 2010 | 31 | 2 | 1 | 2 | 13.10 | 7.90 |
| 2010 | 31 | 2 | 1 | 2 | 9.30 | 4.30 |
| 2010 | 31 | 2 | 1 | 2 | 8.20 | 4.30 |
| 2010 | 31 | 2 | 1 | 2 | 9.90 | 5.10 |
| 2010 | 31 | 2 | 1 | 2 | 11.60 | 7.80 |
| 2010 | 31 | 2 | 1 | 2 | 8.40 | 4.60 |
| 2010 | 31 | 2 | 1 | 2 | 7.10 | 5.70 |
| 2010 | 31 | 2 | 1 | 3 | 7.40 | 4.80 |
| 2010 | 32 | 1 | 5 | 5 | 0.00 | 0.00 |
| 2010 | 32 | 1 | 5 | 5 | 0.00 | 0.00 |
| 2010 | 32 | 1 | 1 | 5 | 2.30 | 0.00 |
| 2010 | 32 | 1 | 1 | 5 | 3.50 | 0.00 |
| 2010 | 32 | 1 | 1 | 5 | 8.70 | 0.00 |
| 2010 | 32 | 1 | 1 | 5 | 4.50 | 0.00 |
| 2010 | 32 | 1 | 1 | 4 | 3.20 | 4.20 |
| 2010 | 32 | 1 | 1 | 4 | 3.50 | 2.90 |
| 2010 | 32 | 1 | 1 | 2 | 5.10 | 2.20 |
| 2010 | 32 | 1 | 1 | 2 | 4.30 | 2.10 |

| | | | | | | |
|---|---|---|---|---|---|---|
| 2010 | 32 | 2 | 1 | 5 | 3.90 | 0.00 |
| 2010 | 32 | 2 | 1 | 2 | 2.80 | 0.50 |
| 2010 | 32 | 2 | 1 | 2 | 3.60 | 0.50 |
| 2010 | 32 | 2 | 1 | 2 | 1.90 | 0.50 |
| 2010 | 32 | 2 | 1 | 2 | 4.90 | 0.50 |
| 2010 | 32 | 2 | 1 | 2 | 10.80 | 0.50 |
| 2010 | 32 | 2 | 1 | 2 | 2.60 | 0.50 |
| 2010 | 32 | 2 | 1 | 2 | 3.80 | 0.50 |
| 2010 | 32 | 2 | 1 | 2 | 3.40 | 0.50 |
| 2010 | 32 | 2 | 1 | 2 | 1.40 | 0.50 |
| 2010 | 33 | 1 | 5 | 5 | 0.00 | 0.00 |
| 2010 | 33 | 1 | 5 | 5 | 0.00 | 0.00 |
| 2010 | 33 | 1 | 5 | 5 | 0.00 | 0.00 |
| 2010 | 33 | 1 | 5 | 5 | 0.00 | 0.00 |
| 2010 | 33 | 1 | 5 | 5 | 0.00 | 0.00 |
| 2010 | 33 | 1 | 1 | 2 | 3.90 | 1.30 |
| 2010 | 33 | 1 | 1 | 2 | 1.20 | 2.90 |
| 2010 | 33 | 1 | 1 | 2 | 4.70 | 1.10 |
| 2010 | 33 | 1 | 1 | 2 | 6.50 | 2.70 |
| 2010 | 33 | 1 | 1 | 2 | 4.20 | 3.90 |
| 2010 | 33 | 2 | 5 | 5 | 0.00 | 0.00 |
| 2010 | 33 | 2 | 5 | 5 | 0.00 | 0.00 |
| 2010 | 33 | 2 | 5 | 5 | 0.00 | 0.00 |
| 2010 | 33 | 2 | 5 | 5 | 0.00 | 0.00 |
| 2010 | 33 | 2 | 5 | 5 | 0.00 | 0.00 |
| 2010 | 33 | 2 | 5 | 2 | 0.00 | 0.50 |
| 2010 | 33 | 2 | 5 | 2 | 0.00 | 0.50 |
| 2010 | 33 | 2 | 1 | 2 | 0.50 | 0.50 |
| 2010 | 33 | 2 | 1 | 2 | 0.50 | 1.20 |
| 2010 | 33 | 2 | 1 | 2 | 5.80 | 3.40 |
| 2010 | 34 | 1 | 1 | 2 | 14.30 | 5.50 |
| 2010 | 34 | 1 | 1 | 2 | 7.90 | 4.40 |
| 2010 | 34 | 1 | 1 | 2 | 11.10 | 3.60 |
| 2010 | 34 | 1 | 1 | 2 | 4.60 | 7.90 |
| 2010 | 34 | 1 | 1 | 2 | 11.30 | 5.60 |
| 2010 | 34 | 1 | 1 | 2 | 12.50 | 5.60 |
| 2010 | 34 | 1 | 1 | 2 | 11.40 | 7.50 |
| 2010 | 34 | 1 | 1 | 2 | 9.80 | 2.50 |
| 2010 | 34 | 1 | 1 | 2 | 13.30 | 9.50 |
| 2010 | 34 | 1 | 1 | 2 | 5.50 | 5.50 |
| 2010 | 34 | 2 | 1 | 2 | 10.20 | 11.00 |
| 2010 | 34 | 2 | 1 | 2 | 9.50 | 6.00 |
| 2010 | 34 | 2 | 1 | 2 | 9.70 | 4.20 |
| 2010 | 34 | 2 | 1 | 2 | 16.60 | 8.90 |
| 2010 | 34 | 2 | 1 | 2 | 13.70 | 5.80 |
| 2010 | 34 | 2 | 1 | 2 | 17.80 | 10.10 |
| 2010 | 34 | 2 | 1 | 2 | 19.90 | 7.30 |
| 2010 | 34 | 2 | 1 | 2 | 13.60 | 11.50 |
| 2010 | 34 | 2 | 1 | 2 | 18.00 | 10.50 |
| 2010 | 34 | 2 | 1 | 2 | 27.20 | 3.00 |
| 2010 | 35 | 1 | 5 | 2 | 0.00 | 4.90 |
| 2010 | 35 | 1 | 1 | 2 | 4.50 | 5.60 |
| 2010 | 35 | 1 | 1 | 2 | 2.70 | 5.20 |
| 2010 | 35 | 1 | 1 | 2 | 5.00 | 5.50 |
| 2010 | 35 | 1 | 1 | 2 | 6.80 | 4.00 |
| 2010 | 35 | 1 | 1 | 2 | 5.40 | 6.50 |
| 2010 | 35 | 1 | 1 | 2 | 3.90 | 5.50 |
| 2010 | 35 | 1 | 1 | 2 | 2.50 | 2.70 |

| | | | | | | |
|---|---|---|---|---|---|---|
| 2010 | 35 | 1 | 1 | 2 | 2.50 | 1.50 |
| 2010 | 35 | 1 | 1 | 2 | 4.10 | 9.40 |
| 2010 | 35 | 2 | 1 | 2 | 21.50 | 4.20 |
| 2010 | 35 | 2 | 1 | 2 | 12.30 | 5.70 |
| 2010 | 35 | 2 | 1 | 2 | 5.60 | 3.60 |
| 2010 | 35 | 2 | 1 | 2 | 10.30 | 7.70 |
| 2010 | 35 | 2 | 1 | 2 | 6.40 | 7.20 |
| 2010 | 35 | 2 | 1 | 2 | 7.30 | 7.10 |
| 2010 | 35 | 2 | 1 | 2 | 14.60 | 13.10 |
| 2010 | 35 | 2 | 1 | 2 | 11.10 | 8.40 |
| 2010 | 35 | 2 | 1 | 2 | 10.20 | 9.40 |
| 2010 | 35 | 2 | 1 | 2 | 9.60 | 10.90 |
| 2010 | 36 | 1 | 1 | 4 | 7.20 | 8.30 |
| 2010 | 36 | 1 | 1 | 4 | 9.50 | 5.50 |
| 2010 | 36 | 1 | 1 | 4 | 9.00 | 6.00 |
| 2010 | 36 | 1 | 1 | 2 | 13.60 | 6.20 |
| 2010 | 36 | 1 | 1 | 2 | 11.20 | 11.00 |
| 2010 | 36 | 1 | 1 | 2 | 15.50 | 10.10 |
| 2010 | 36 | 1 | 1 | 2 | 16.50 | 6.20 |
| 2010 | 36 | 1 | 1 | 2 | 11.60 | 6.90 |
| 2010 | 36 | 1 | 1 | 2 | 11.90 | 0.50 |
| 2010 | 36 | 1 | 2 | 2 | 13.20 | 11.00 |
| 2010 | 36 | 2 | 5 | 2 | 0.00 | 14.60 |
| 2010 | 36 | 2 | 5 | 2 | 0.00 | 13.80 |
| 2010 | 36 | 2 | 1 | 2 | 18.40 | 3.50 |
| 2010 | 36 | 2 | 1 | 2 | 19.20 | 6.10 |
| 2010 | 36 | 2 | 1 | 2 | 10.60 | 5.50 |
| 2010 | 36 | 2 | 1 | 2 | 21.50 | 13.30 |
| 2010 | 36 | 2 | 1 | 2 | 19.90 | 12.40 |
| 2010 | 36 | 2 | 1 | 2 | 13.60 | 12.90 |
| 2010 | 36 | 2 | 1 | 2 | 11.40 | 13.20 |
| 2010 | 36 | 2 | 1 | 2 | 4.90 | 8.20 |
| 2010 | 37 | 1 | 3 | 5 | 4.80 | 0.00 |
| 2010 | 37 | 1 | 3 | 5 | 11.10 | 0.00 |
| 2010 | 37 | 1 | 3 | 4 | 4.90 | 4.50 |
| 2010 | 37 | 1 | 1 | 2 | 2.60 | 0.20 |
| 2010 | 37 | 1 | 1 | 2 | 7.50 | 3.60 |
| 2010 | 37 | 1 | 1 | 2 | 9.10 | 4.50 |
| 2010 | 37 | 1 | 1 | 2 | 3.90 | 0.50 |
| 2010 | 37 | 1 | 1 | 2 | 10.40 | 2.40 |
| 2010 | 37 | 1 | 1 | 2 | 7.60 | 3.00 |
| 2010 | 37 | 1 | 1 | 2 | 8.90 | 6.10 |
| 2010 | 37 | 2 | 5 | 5 | 0.00 | 0.00 |
| 2010 | 37 | 2 | 1 | 5 | 4.50 | 0.00 |
| 2010 | 37 | 2 | 1 | 5 | 11.20 | 0.00 |
| 2010 | 37 | 2 | 1 | 5 | 10.20 | 0.00 |
| 2010 | 37 | 2 | 1 | 5 | 11.10 | 0.00 |
| 2010 | 37 | 2 | 1 | 5 | 17.10 | 0.00 |
| 2010 | 37 | 2 | 1 | 2 | 12.20 | 1.20 |
| 2010 | 37 | 2 | 2 | 2 | 10.60 | 2.10 |
| 2010 | 37 | 2 | 1 | 1 | 16.40 | 3.20 |
| 2010 | 37 | 2 | 1 | 1 | 14.00 | 1.10 |
| 2010 | 38 | 1 | 5 | 5 | 0.00 | 0.00 |
| 2010 | 38 | 1 | 5 | 5 | 0.00 | 0.00 |
| 2010 | 38 | 1 | 1 | 5 | 5.60 | 0.00 |
| 2010 | 38 | 1 | 1 | 5 | 10.50 | 0.00 |
| 2010 | 38 | 1 | 1 | 2 | 9.30 | 1.00 |
| 2010 | 38 | 1 | 1 | 2 | 7.90 | 0.40 |

| | | | | | | |
|---|---|---|---|---|---|---|
| 2010 | 38 | 1 | 1 | 2 | 7.90 | 2.10 |
| 2010 | 38 | 1 | 1 | 2 | 8.70 | 3.50 |
| 2010 | 38 | 1 | 1 | 2 | 5.40 | 2.10 |
| 2010 | 38 | 1 | 1 | 2 | 2.30 | 6.40 |
| 2010 | 38 | 2 | 3 | 5 | 10.30 | 0.00 |
| 2010 | 38 | 2 | 1 | 5 | 12.10 | 0.00 |
| 2010 | 38 | 2 | 1 | 5 | 13.60 | 0.00 |
| 2010 | 38 | 2 | 1 | 5 | 12.90 | 0.00 |
| 2010 | 38 | 2 | 1 | 5 | 12.30 | 0.00 |
| 2010 | 38 | 2 | 1 | 5 | 10.10 | 0.00 |
| 2010 | 38 | 2 | 1 | 5 | 12.70 | 0.00 |
| 2010 | 38 | 2 | 1 | 5 | 10.20 | 0.00 |
| 2010 | 38 | 2 | 1 | 2 | 11.30 | 4.80 |
| 2010 | 38 | 2 | 5 | 4 | 0.00 | 6.60 |
| 2010 | 39 | 1 | 5 | 2 | 0.00 | 5.00 |
| 2010 | 39 | 1 | 5 | 2 | 0.00 | 6.40 |
| 2010 | 39 | 1 | 5 | 2 | 0.00 | 6.50 |
| 2010 | 39 | 1 | 1 | 2 | 1.10 | 2.30 |
| 2010 | 39 | 1 | 1 | 2 | 1.90 | 7.40 |
| 2010 | 39 | 1 | 1 | 2 | 1.50 | 0.50 |
| 2010 | 39 | 1 | 1 | 2 | 1.60 | 2.90 |
| 2010 | 39 | 1 | 1 | 2 | 1.50 | 2.30 |
| 2010 | 39 | 1 | 1 | 2 | 1.00 | 9.50 |
| 2010 | 39 | 1 | 1 | 2 | 2.50 | 10.50 |
| 2010 | 39 | 2 | 5 | 5 | 0.00 | 0.00 |
| 2010 | 39 | 2 | 5 | 2 | 0.00 | 3.80 |
| 2010 | 39 | 2 | 5 | 2 | 0.00 | 1.90 |
| 2010 | 39 | 2 | 5 | 2 | 0.00 | 5.60 |
| 2010 | 39 | 2 | 5 | 2 | 0.00 | 1.70 |
| 2010 | 39 | 2 | 1 | 2 | 2.60 | 8.10 |
| 2010 | 39 | 2 | 1 | 2 | 10.10 | 3.30 |
| 2010 | 39 | 2 | 1 | 2 | 7.10 | 8.50 |
| 2010 | 39 | 2 | 3 | 2 | 2.50 | 9.60 |
| 2010 | 39 | 2 | 1 | 2 | 4.10 | 8.90 |
| 2010 | 40 | 1 | 3 | 4 | 4.60 | 5.80 |
| 2010 | 40 | 1 | 1 | 2 | 11.10 | 4.40 |
| 2010 | 40 | 1 | 1 | 2 | 10.10 | 6.90 |
| 2010 | 40 | 1 | 1 | 2 | 8.50 | 4.50 |
| 2010 | 40 | 1 | 1 | 2 | 8.20 | 6.60 |
| 2010 | 40 | 1 | 1 | 2 | 9.00 | 7.50 |
| 2010 | 40 | 1 | 1 | 2 | 8.50 | 8.70 |
| 2010 | 40 | 1 | 1 | 2 | 12.00 | 4.60 |
| 2010 | 40 | 1 | 1 | 2 | 8.60 | 9.50 |
| 2010 | 40 | 1 | 1 | 2 | 12.00 | 7.50 |
| 2010 | 40 | 2 | 5 | 2 | 0.00 | 12.70 |
| 2010 | 40 | 2 | 1 | 2 | 6.00 | 3.50 |
| 2010 | 40 | 2 | 1 | 2 | 10.10 | 0.20 |
| 2010 | 40 | 2 | 1 | 2 | 12.10 | 4.00 |
| 2010 | 40 | 2 | 1 | 2 | 10.30 | 3.50 |
| 2010 | 40 | 2 | 1 | 2 | 11.40 | 5.60 |
| 2010 | 40 | 2 | 1 | 2 | 9.50 | 4.90 |
| 2010 | 40 | 2 | 1 | 2 | 12.60 | 3.20 |
| 2010 | 40 | 2 | 1 | 2 | 15.30 | 9.90 |
| 2010 | 40 | 2 | 1 | 2 | 12.30 | 8.60 |
| 2022 | 41 | 1 | 1 | 5 | 10.20 | 0.00 |
| 2022 | 41 | 1 | 1 | 5 | 9.00 | 0.00 |
| 2022 | 41 | 1 | 5 | 5 | 0.00 | 0.00 |
| 2022 | 41 | 1 | 5 | 5 | 0.00 | 0.00 |

| | | | | | | |
|---|---|---|---|---|---|---|
| 2022 | 41 | 1 | 5 | 5 | 0.00 | 0.00 |
| 2022 | 41 | 1 | 5 | 5 | 0.00 | 0.00 |
| 2022 | 41 | 1 | 5 | 2 | 0.00 | 4.80 |
| 2022 | 41 | 1 | 5 | 2 | 0.00 | 3.90 |
| 2022 | 41 | 1 | 5 | 2 | 0.00 | 6.50 |
| 2022 | 41 | 1 | 5 | 4 | 0.00 | 4.20 |
| 2022 | 41 | 2 | 5 | 5 | 0.00 | 0.00 |
| 2022 | 41 | 2 | 5 | 5 | 0.00 | 0.00 |
| 2022 | 41 | 2 | 5 | 5 | 0.00 | 0.00 |
| 2022 | 41 | 2 | 5 | 5 | 0.00 | 0.00 |
| 2022 | 41 | 2 | 5 | 5 | 0.00 | 0.00 |
| 2022 | 41 | 2 | 5 | 5 | 0.00 | 0.00 |
| 2022 | 41 | 2 | 5 | 2 | 0.00 | 0.50 |
| 2022 | 41 | 2 | 5 | 2 | 0.00 | 0.50 |
| 2022 | 41 | 2 | 5 | 2 | 0.00 | 2.20 |
| 2022 | 41 | 2 | 1 | 2 | 17.30 | 6.50 |
| 2022 | 42 | 1 | 1 | 5 | 12.30 | 0.00 |
| 2022 | 42 | 1 | 1 | 5 | 14.50 | 0.00 |
| 2022 | 42 | 1 | 1 | 5 | 15.50 | 0.00 |
| 2022 | 42 | 1 | 1 | 5 | 13.20 | 0.00 |
| 2022 | 42 | 1 | 1 | 5 | 10.70 | 0.00 |
| 2022 | 42 | 1 | 1 | 2 | 10.40 | 0.50 |
| 2022 | 42 | 1 | 5 | 2 | 0.00 | 0.50 |
| 2022 | 42 | 1 | 5 | 2 | 0.00 | 7.90 |
| 2022 | 42 | 1 | 5 | 2 | 0.00 | 8.90 |
| 2022 | 42 | 1 | 5 | 2 | 0.00 | 4.60 |
| 2022 | 42 | 2 | 5 | 5 | 0.00 | 0.00 |
| 2022 | 42 | 2 | 5 | 5 | 0.00 | 0.00 |
| 2022 | 42 | 2 | 5 | 5 | 0.00 | 0.00 |
| 2022 | 42 | 2 | 5 | 5 | 0.00 | 0.00 |
| 2022 | 42 | 2 | 1 | 4 | 25.00 | 6.30 |
| 2022 | 42 | 2 | 1 | 4 | 20.20 | 5.50 |
| 2022 | 42 | 2 | 1 | 2 | 9.80 | 3.90 |
| 2022 | 42 | 2 | 1 | 2 | 13.30 | 4.60 |
| 2022 | 42 | 2 | 1 | 3 | 10.70 | 2.20 |
| 2022 | 42 | 2 | 1 | 3 | 8.90 | 2.20 |
| 2022 | 43 | 1 | 1 | 5 | 6.00 | 0.00 |
| 2022 | 43 | 1 | 1 | 5 | 7.00 | 0.00 |
| 2022 | 43 | 1 | 1 | 5 | 7.50 | 0.00 |
| 2022 | 43 | 1 | 1 | 5 | 5.00 | 0.00 |
| 2022 | 43 | 1 | 5 | 5 | 0.00 | 0.00 |
| 2022 | 43 | 1 | 5 | 2 | 0.00 | 7.00 |
| 2022 | 43 | 1 | 5 | 2 | 0.00 | 10.00 |
| 2022 | 43 | 1 | 5 | 2 | 0.00 | 12.00 |
| 2022 | 43 | 1 | 5 | 2 | 0.00 | 11.00 |
| 2022 | 43 | 1 | 5 | 2 | 0.00 | 10.00 |
| 2022 | 43 | 2 | 5 | 5 | 0.00 | 0.00 |
| 2022 | 43 | 2 | 5 | 5 | 0.00 | 0.00 |
| 2022 | 43 | 2 | 5 | 5 | 0.00 | 0.00 |
| 2022 | 43 | 2 | 1 | 5 | 6.50 | 0.00 |
| 2022 | 43 | 2 | 1 | 4 | 12.00 | 4.00 |
| 2022 | 43 | 2 | 1 | 3 | 10.00 | 2.00 |
| 2022 | 43 | 2 | 1 | 3 | 19.00 | 13.00 |
| 2022 | 43 | 2 | 1 | 3 | 6.00 | 5.00 |
| 2022 | 43 | 2 | 1 | 2 | 13.00 | 5.50 |
| 2022 | 43 | 2 | 1 | 2 | 6.50 | 13.00 |
| 2022 | 44 | 1 | 1 | 5 | 22.30 | 0.00 |
| 2022 | 44 | 1 | 1 | 5 | 14.50 | 0.00 |

| | | | | | | |
|---|---|---|---|---|---|---|
| 2022 | 44 | 1 | 1 | 5 | 14.30 | 0.00 |
| 2022 | 44 | 1 | 1 | 5 | 25.20 | 0.00 |
| 2022 | 44 | 1 | 1 | 4 | 18.30 | 21.50 |
| 2022 | 44 | 1 | 1 | 4 | 7.50 | 14.50 |
| 2022 | 44 | 1 | 1 | 4 | 17.80 | 16.20 |
| 2022 | 44 | 1 | 1 | 2 | 0.00 | 17.50 |
| 2022 | 44 | 1 | 5 | 2 | 0.00 | 7.50 |
| 2022 | 44 | 1 | 5 | 2 | 0.00 | 8.90 |
| 2022 | 44 | 2 | 5 | 5 | 0.00 | 0.00 |
| 2022 | 44 | 2 | 5 | 5 | 0.00 | 0.00 |
| 2022 | 44 | 2 | 1 | 5 | 12.30 | 0.00 |
| 2022 | 44 | 2 | 1 | 5 | 23.70 | 0.00 |
| 2022 | 44 | 2 | 1 | 5 | 19.60 | 0.00 |
| 2022 | 44 | 2 | 1 | 4 | 21.00 | 6.50 |
| 2022 | 44 | 2 | 1 | 4 | 14.90 | 2.70 |
| 2022 | 44 | 2 | 1 | 2 | 2.50 | 13.50 |
| 2022 | 44 | 2 | 1 | 2 | 4.50 | 14.30 |
| 2022 | 44 | 2 | 1 | 2 | 16.40 | 12.50 |
| 2022 | 45 | 1 | 1 | 4 | 3.50 | 5.20 |
| 2022 | 45 | 1 | 1 | 2 | 7.60 | 10.00 |
| 2022 | 45 | 1 | 1 | 2 | 8.10 | 8.50 |
| 2022 | 45 | 1 | 1 | 2 | 5.40 | 7.00 |
| 2022 | 45 | 1 | 1 | 2 | 8.60 | 8.10 |
| 2022 | 45 | 1 | 1 | 2 | 5.50 | 1.50 |
| 2022 | 45 | 1 | 1 | 2 | 7.60 | 3.00 |
| 2022 | 45 | 1 | 1 | 2 | 12.30 | 6.10 |
| 2022 | 45 | 1 | 1 | 2 | 10.10 | 8.30 |
| 2022 | 45 | 1 | 1 | 2 | 6.70 | 0.50 |
| 2022 | 45 | 2 | 5 | 2 | 0.00 | 5.50 |
| 2022 | 45 | 2 | 5 | 2 | 0.00 | 8.00 |
| 2022 | 45 | 2 | 1 | 2 | 4.50 | 3.20 |
| 2022 | 45 | 2 | 1 | 2 | 6.00 | 6.00 |
| 2022 | 45 | 2 | 1 | 2 | 11.50 | 5.50 |
| 2022 | 45 | 2 | 1 | 2 | 5.90 | 3.20 |
| 2022 | 45 | 2 | 1 | 2 | 0.50 | 2.00 |
| 2022 | 45 | 2 | 1 | 2 | 5.50 | 3.00 |
| 2022 | 45 | 2 | 1 | 2 | 10.50 | 4.20 |
| 2022 | 45 | 2 | 1 | 2 | 5.60 | 8.70 |
| 2022 | 46 | 1 | 5 | 4 | 0.00 | 5.30 |
| 2022 | 46 | 1 | 5 | 2 | 0.00 | 11.20 |
| 2022 | 46 | 1 | 5 | 5 | 0.00 | 0.00 |
| 2022 | 46 | 1 | 5 | 5 | 0.00 | 0.00 |
| 2022 | 46 | 1 | 5 | 5 | 0.00 | 0.00 |
| 2022 | 46 | 1 | 1 | 5 | 24.50 | 0.00 |
| 2022 | 46 | 1 | 1 | 5 | 22.30 | 0.00 |
| 2022 | 46 | 1 | 1 | 5 | 4.50 | 0.00 |
| 2022 | 46 | 1 | 1 | 5 | 7.30 | 0.00 |
| 2022 | 46 | 1 | 1 | 5 | 5.50 | 0.00 |
| 2022 | 46 | 2 | 5 | 2 | 0.00 | 13.30 |
| 2022 | 46 | 2 | 5 | 4 | 0.00 | 12.70 |
| 2022 | 46 | 2 | 5 | 4 | 0.00 | 2.50 |
| 2022 | 46 | 2 | 5 | 4 | 0.00 | 8.30 |
| 2022 | 46 | 2 | 5 | 4 | 0.00 | 12.30 |
| 2022 | 46 | 2 | 5 | 5 | 0.00 | 0.00 |
| 2022 | 46 | 2 | 5 | 5 | 0.00 | 0.00 |
| 2022 | 46 | 2 | 1 | 5 | 12.60 | 0.00 |
| 2022 | 46 | 2 | 1 | 5 | 14.30 | 0.00 |
| 2022 | 46 | 2 | 1 | 5 | 5.50 | 0.00 |

| | | | | | | |
|---|---|---|---|---|---|---|
| 2022 | 47 | 1 | 1 | 2 | 10.80 | 0.50 |
| 2022 | 47 | 1 | 1 | 2 | 11.30 | 0.50 |
| 2022 | 47 | 1 | 1 | 2 | 1.50 | 0.50 |
| 2022 | 47 | 1 | 1 | 2 | 0.50 | 4.00 |
| 2022 | 47 | 1 | 1 | 2 | 3.50 | 0.50 |
| 2022 | 47 | 1 | 1 | 2 | 5.70 | 0.50 |
| 2022 | 47 | 1 | 1 | 2 | 5.50 | 1.50 |
| 2022 | 47 | 1 | 1 | 2 | 3.50 | 3.50 |
| 2022 | 47 | 1 | 1 | 2 | 3.70 | 4.60 |
| 2022 | 47 | 1 | 1 | 2 | 4.00 | 5.70 |
| 2022 | 47 | 2 | 1 | 2 | 5.50 | 0.50 |
| 2022 | 47 | 2 | 1 | 2 | 3.50 | 0.50 |
| 2022 | 47 | 2 | 1 | 2 | 6.70 | 0.50 |
| 2022 | 47 | 2 | 1 | 2 | 6.90 | 3.50 |
| 2022 | 47 | 2 | 1 | 2 | 5.80 | 4.20 |
| 2022 | 47 | 2 | 1 | 2 | 8.55 | 9.70 |
| 2022 | 47 | 2 | 1 | 2 | 0.50 | 4.80 |
| 2022 | 47 | 2 | 1 | 2 | 3.50 | 9.50 |
| 2022 | 47 | 2 | 1 | 2 | 3.70 | 16.20 |
| 2022 | 47 | 2 | 1 | 2 | 4.50 | 0.50 |
| 2022 | 48 | 1 | 5 | 5 | 0.00 | 0.00 |
| 2022 | 48 | 1 | 5 | 5 | 0.00 | 0.00 |
| 2022 | 48 | 1 | 5 | 5 | 0.00 | 0.00 |
| 2022 | 48 | 1 | 5 | 5 | 0.00 | 0.00 |
| 2022 | 48 | 1 | 5 | 5 | 0.00 | 0.00 |
| 2022 | 48 | 1 | 5 | 5 | 0.00 | 0.00 |
| 2022 | 48 | 1 | 5 | 5 | 0.00 | 0.00 |
| 2022 | 48 | 1 | 5 | 2 | 0.00 | 6.00 |
| 2022 | 48 | 1 | 5 | 2 | 0.00 | 2.00 |
| 2022 | 48 | 1 | 1 | 2 | 5.00 | 2.50 |
| 2022 | 48 | 2 | 5 | 5 | 0.00 | 0.00 |
| 2022 | 48 | 2 | 5 | 5 | 0.00 | 0.00 |
| 2022 | 48 | 2 | 5 | 5 | 0.00 | 0.00 |
| 2022 | 48 | 2 | 5 | 5 | 0.00 | 0.00 |
| 2022 | 48 | 2 | 5 | 5 | 0.00 | 0.00 |
| 2022 | 48 | 2 | 1 | 5 | 3.00 | 0.00 |
| 2022 | 48 | 2 | 1 | 5 | 13.00 | 0.00 |
| 2022 | 48 | 2 | 1 | 3 | 11.00 | 1.50 |
| 2022 | 48 | 2 | 1 | 2 | 13.00 | 4.50 |
| 2022 | 48 | 2 | 2 | 2 | 12.00 | 6.00 |
| 2022 | 49 | 1 | 1 | 2 | 12.30 | 2.50 |
| 2022 | 49 | 1 | 1 | 2 | 2.50 | 2.50 |
| 2022 | 49 | 1 | 1 | 2 | 3.50 | 2.50 |
| 2022 | 49 | 1 | 1 | 2 | 4.60 | 3.70 |
| 2022 | 49 | 1 | 1 | 2 | 4.30 | 4.20 |
| 2022 | 49 | 1 | 1 | 2 | 6.30 | 5.40 |
| 2022 | 49 | 1 | 1 | 2 | 2.50 | 4.50 |
| 2022 | 49 | 1 | 1 | 2 | 2.40 | 1.50 |
| 2022 | 49 | 1 | 1 | 2 | 1.80 | 3.00 |
| 2022 | 49 | 1 | 1 | 2 | 2.30 | 2.50 |
| 2022 | 49 | 2 | 5 | 2 | 0.00 | 0.50 |
| 2022 | 49 | 2 | 5 | 2 | 0.00 | 0.50 |
| 2022 | 49 | 2 | 1 | 2 | 1.50 | 0.50 |
| 2022 | 49 | 2 | 1 | 2 | 2.50 | 1.50 |
| 2022 | 49 | 2 | 1 | 2 | 1.50 | 4.00 |
| 2022 | 49 | 2 | 1 | 2 | 2.70 | 3.60 |
| 2022 | 49 | 2 | 1 | 2 | 5.40 | 1.50 |
| 2022 | 49 | 2 | 1 | 2 | 0.50 | 2.00 |

| | | | | | | |
|---|---|---|---|---|---|---|
| 2022 | 49 | 2 | 1 | 2 | 2.60 | 0.50 |
| 2022 | 49 | 2 | 1 | 5 | 0.50 | 0.00 |
| 2022 | 50 | 1 | 5 | 4 | 0.00 | 4.40 |
| 2022 | 50 | 1 | 5 | 4 | 0.00 | 0.50 |
| 2022 | 50 | 1 | 5 | 5 | 0.00 | 0.00 |
| 2022 | 50 | 1 | 5 | 5 | 0.00 | 0.00 |
| 2022 | 50 | 1 | 1 | 5 | 5.60 | 0.00 |
| 2022 | 50 | 1 | 1 | 5 | 11.20 | 0.00 |
| 2022 | 50 | 1 | 1 | 5 | 16.20 | 0.00 |
| 2022 | 50 | 1 | 1 | 5 | 0.50 | 0.00 |
| 2022 | 50 | 1 | 1 | 5 | 4.50 | 0.00 |
| 2022 | 50 | 1 | 2 | 5 | 13.20 | 0.00 |
| 2022 | 50 | 2 | 1 | 4 | 21.50 | 4.90 |
| 2022 | 50 | 2 | 1 | 4 | 8.50 | 9.50 |
| 2022 | 50 | 2 | 5 | 5 | 0.00 | 0.00 |
| 2022 | 50 | 2 | 5 | 5 | 0.00 | 0.00 |
| 2022 | 50 | 2 | 5 | 5 | 0.00 | 0.00 |
| 2022 | 50 | 2 | 5 | 5 | 0.00 | 0.00 |
| 2022 | 50 | 2 | 5 | 5 | 0.00 | 0.00 |
| 2022 | 50 | 2 | 5 | 5 | 0.00 | 0.00 |
| 2022 | 50 | 2 | 5 | 5 | 0.00 | 0.00 |
| 2022 | 50 | 2 | 5 | 5 | 0.00 | 0.00 |
| 2022 | 51 | 1 | 1 | 5 | 8.50 | 0.00 |
| 2022 | 51 | 1 | 5 | 5 | 0.00 | 0.00 |
| 2022 | 51 | 1 | 5 | 5 | 0.00 | 0.00 |
| 2022 | 51 | 1 | 5 | 5 | 0.00 | 0.00 |
| 2022 | 51 | 1 | 5 | 5 | 0.00 | 0.00 |
| 2022 | 51 | 1 | 5 | 5 | 0.00 | 0.00 |
| 2022 | 51 | 1 | 5 | 5 | 0.00 | 0.00 |
| 2022 | 51 | 1 | 5 | 5 | 0.00 | 0.00 |
| 2022 | 51 | 1 | 5 | 5 | 0.00 | 0.00 |
| 2022 | 51 | 1 | 5 | 5 | 0.00 | 0.00 |
| 2022 | 51 | 2 | 1 | 5 | 14.30 | 0.00 |
| 2022 | 51 | 2 | 1 | 5 | 16.50 | 0.00 |
| 2022 | 51 | 2 | 1 | 5 | 4.70 | 0.00 |
| 2022 | 51 | 2 | 1 | 5 | 0.50 | 0.00 |
| 2022 | 51 | 2 | 5 | 5 | 0.00 | 0.00 |
| 2022 | 51 | 2 | 5 | 5 | 0.00 | 0.00 |
| 2022 | 51 | 2 | 5 | 5 | 0.00 | 0.00 |
| 2022 | 51 | 2 | 5 | 5 | 0.00 | 0.00 |
| 2022 | 51 | 2 | 5 | 5 | 0.00 | 0.00 |
| 2022 | 51 | 2 | 5 | 5 | 0.00 | 0.00 |
| 2022 | 52 | 1 | 1 | 2 | 9.50 | 0.50 |
| 2022 | 52 | 1 | 1 | 2 | 10.20 | 1.50 |
| 2022 | 52 | 1 | 1 | 2 | 15.20 | 3.50 |
| 2022 | 52 | 1 | 1 | 2 | 1.50 | 8.10 |
| 2022 | 52 | 1 | 1 | 2 | 7.40 | 10.00 |
| 2022 | 52 | 1 | 1 | 2 | 4.20 | 9.00 |
| 2022 | 52 | 1 | 1 | 2 | 11.60 | 4.50 |
| 2022 | 52 | 1 | 1 | 2 | 10.30 | 5.00 |
| 2022 | 52 | 1 | 1 | 2 | 7.40 | 9.20 |
| 2022 | 52 | 1 | 1 | 2 | 6.20 | 5.60 |
| 2022 | 52 | 2 | 5 | 1 | 0.00 | 9.50 |
| 2022 | 52 | 2 | 1 | 2 | 11.50 | 7.90 |
| 2022 | 52 | 2 | 1 | 2 | 10.30 | 7.40 |
| 2022 | 52 | 2 | 1 | 2 | 9.50 | 10.00 |
| 2022 | 52 | 2 | 1 | 2 | 8.20 | 6.20 |
| 2022 | 52 | 2 | 1 | 2 | 7.40 | 7.30 |

| | | | | | | |
|---|---|---|---|---|---|---|
| 2022 | 52 | 2 | 1 | 2 | 6.50 | 6.50 |
| 2022 | 52 | 2 | 1 | 2 | 0.50 | 6.70 |
| 2022 | 52 | 2 | 1 | 2 | 2.50 | 2.90 |
| 2022 | 52 | 2 | 1 | 2 | 5.70 | 0.50 |
| 2022 | 53 | 1 | 5 | 2 | 0.00 | 0.50 |
| 2022 | 53 | 1 | 1 | 2 | 7.50 | 5.60 |
| 2022 | 53 | 1 | 1 | 2 | 8.20 | 3.50 |
| 2022 | 53 | 1 | 1 | 2 | 9.40 | 4.50 |
| 2022 | 53 | 1 | 1 | 2 | 0.50 | 6.40 |
| 2022 | 53 | 1 | 1 | 2 | 11.50 | 3.60 |
| 2022 | 53 | 1 | 1 | 2 | 12.10 | 7.00 |
| 2022 | 53 | 1 | 1 | 2 | 9.50 | 4.30 |
| 2022 | 53 | 1 | 1 | 2 | 8.40 | 5.20 |
| 2022 | 53 | 1 | 1 | 2 | 7.50 | 4.00 |
| 2022 | 53 | 2 | 1 | 5 | 2.50 | 0.00 |
| 2022 | 53 | 2 | 1 | 5 | 8.10 | 0.00 |
| 2022 | 53 | 2 | 1 | 2 | 4.50 | 2.40 |
| 2022 | 53 | 2 | 1 | 2 | 0.50 | 5.60 |
| 2022 | 53 | 2 | 1 | 2 | 0.50 | 5.50 |
| 2022 | 53 | 2 | 1 | 2 | 7.60 | 6.00 |
| 2022 | 53 | 2 | 1 | 2 | 8.50 | 10.50 |
| 2022 | 53 | 2 | 1 | 2 | 9.20 | 3.00 |
| 2022 | 53 | 2 | 5 | 2 | 0.00 | 4.90 |
| 2022 | 53 | 2 | 5 | 2 | 0.00 | 0.50 |
| 2022 | 54 | 1 | 5 | 5 | 0.00 | 0.00 |
| 2022 | 54 | 1 | 5 | 2 | 0.00 | 10.00 |
| 2022 | 54 | 1 | 5 | 1 | 0.00 | 14.00 |
| 2022 | 54 | 1 | 5 | 3 | 0.00 | 15.00 |
| 2022 | 54 | 1 | 1 | 3 | 9.00 | 12.50 |
| 2022 | 54 | 1 | 1 | 2 | 17.50 | 12.00 |
| 2022 | 54 | 1 | 1 | 2 | 15.50 | 19.50 |
| 2022 | 54 | 1 | 1 | 2 | 16.00 | 15.00 |
| 2022 | 54 | 1 | 1 | 2 | 20.00 | 20.50 |
| 2022 | 54 | 1 | 1 | 3 | 11.00 | 18.00 |
| 2022 | 54 | 2 | 5 | 5 | 0.00 | 0.00 |
| 2022 | 54 | 2 | 5 | 5 | 0.00 | 0.00 |
| 2022 | 54 | 2 | 1 | 5 | 18.00 | 0.00 |
| 2022 | 54 | 2 | 1 | 5 | 17.50 | 0.00 |
| 2022 | 54 | 2 | 1 | 5 | 21.00 | 0.00 |
| 2022 | 54 | 2 | 1 | 2 | 21.00 | 14.50 |
| 2022 | 54 | 2 | 1 | 2 | 17.50 | 12.00 |
| 2022 | 54 | 2 | 1 | 3 | 19.50 | 11.00 |
| 2022 | 54 | 2 | 1 | 3 | 20.50 | 17.00 |
| 2022 | 54 | 2 | 1 | 1 | 18.00 | 13.00 |
| 2022 | 55 | 1 | 5 | 5 | 0.00 | 0.00 |
| 2022 | 55 | 1 | 5 | 5 | 0.00 | 0.00 |
| 2022 | 55 | 1 | 5 | 5 | 0.00 | 0.00 |
| 2022 | 55 | 1 | 5 | 5 | 0.00 | 0.00 |
| 2022 | 55 | 1 | 5 | 5 | 0.00 | 0.00 |
| 2022 | 55 | 1 | 5 | 5 | 0.00 | 0.00 |
| 2022 | 55 | 1 | 1 | 3 | 8.00 | 4.50 |
| 2022 | 55 | 1 | 1 | 2 | 3.50 | 9.00 |
| 2022 | 55 | 1 | 1 | 2 | 8.50 | 8.00 |
| 2022 | 55 | 1 | 2 | 2 | 2.50 | 6.50 |
| 2022 | 55 | 2 | 5 | 5 | 0.00 | 0.00 |
| 2022 | 55 | 2 | 5 | 5 | 0.00 | 0.00 |
| 2022 | 55 | 2 | 5 | 5 | 0.00 | 0.00 |
| 2022 | 55 | 2 | 5 | 5 | 0.00 | 0.00 |

| | | | | | | |
|---|---|---|---|---|---|---|
| 2022 | 55 | 2 | 5 | 5 | 0.00 | 0.00 |
| 2022 | 55 | 2 | 5 | 5 | 0.00 | 0.00 |
| 2022 | 55 | 2 | 5 | 5 | 0.00 | 0.00 |
| 2022 | 55 | 2 | 5 | 2 | 0.00 | 3.50 |
| 2022 | 55 | 2 | 1 | 2 | 3.00 | 15.00 |
| 2022 | 55 | 2 | 1 | 2 | 19.00 | 10.00 |
| 2022 | 56 | 1 | 1 | 5 | 3.00 | 0.00 |
| 2022 | 56 | 1 | 1 | 5 | 9.00 | 0.00 |
| 2022 | 56 | 1 | 1 | 5 | 14.00 | 0.00 |
| 2022 | 56 | 1 | 5 | 5 | 0.00 | 0.00 |
| 2022 | 56 | 1 | 5 | 5 | 0.00 | 0.00 |
| 2022 | 56 | 1 | 5 | 5 | 0.00 | 0.00 |
| 2022 | 56 | 1 | 5 | 5 | 0.00 | 0.00 |
| 2022 | 56 | 1 | 5 | 5 | 0.00 | 0.00 |
| 2022 | 56 | 1 | 5 | 5 | 0.00 | 0.00 |
| 2022 | 56 | 1 | 5 | 5 | 0.00 | 0.00 |
| 2022 | 56 | 2 | 1 | 4 | 16.00 | 12.00 |
| 2022 | 56 | 2 | 5 | 5 | 0.00 | 0.00 |
| 2022 | 56 | 2 | 5 | 5 | 0.00 | 0.00 |
| 2022 | 56 | 2 | 5 | 5 | 0.00 | 0.00 |
| 2022 | 56 | 2 | 5 | 5 | 0.00 | 0.00 |
| 2022 | 56 | 2 | 5 | 5 | 0.00 | 0.00 |
| 2022 | 56 | 2 | 5 | 5 | 0.00 | 0.00 |
| 2022 | 56 | 2 | 5 | 5 | 0.00 | 0.00 |
| 2022 | 56 | 2 | 5 | 5 | 0.00 | 0.00 |
| 2022 | 56 | 2 | 5 | 5 | 0.00 | 0.00 |
| 2022 | 57 | 1 | 1 | 5 | 0.50 | 0.00 |
| 2022 | 57 | 1 | 1 | 5 | 0.50 | 0.00 |
| 2022 | 57 | 1 | 1 | 5 | 0.50 | 0.00 |
| 2022 | 57 | 1 | 1 | 2 | 4.50 | 0.50 |
| 2022 | 57 | 1 | 1 | 2 | 6.30 | 0.50 |
| 2022 | 57 | 1 | 1 | 2 | 7.10 | 0.50 |
| 2022 | 57 | 1 | 1 | 2 | 2.50 | 1.50 |
| 2022 | 57 | 1 | 1 | 2 | 3.40 | 4.00 |
| 2022 | 57 | 1 | 1 | 2 | 5.40 | 3.50 |
| 2022 | 57 | 1 | 1 | 2 | 7.30 | 5.20 |
| 2022 | 57 | 2 | 5 | 5 | 0.00 | 0.00 |
| 2022 | 57 | 2 | 5 | 2 | 0.00 | 3.50 |
| 2022 | 57 | 2 | 1 | 2 | 6.50 | 5.20 |
| 2022 | 57 | 2 | 1 | 2 | 5.40 | 0.50 |
| 2022 | 57 | 2 | 1 | 2 | 3.50 | 0.40 |
| 2022 | 57 | 2 | 1 | 2 | 5.90 | 3.20 |
| 2022 | 57 | 2 | 1 | 2 | 1.50 | 5.40 |
| 2022 | 57 | 2 | 1 | 2 | 1.50 | 2.90 |
| 2022 | 57 | 2 | 1 | 2 | 7.70 | 5.80 |
| 2022 | 57 | 2 | 1 | 2 | 4.20 | 8.00 |
| 2022 | 58 | 1 | 1 | 2 | 12.00 | 7.00 |
| 2022 | 58 | 1 | 1 | 5 | 12.00 | 0.00 |
| 2022 | 58 | 1 | 2 | 5 | 2.50 | 0.00 |
| 2022 | 58 | 1 | 5 | 5 | 0.00 | 0.00 |
| 2022 | 58 | 1 | 5 | 5 | 0.00 | 0.00 |
| 2022 | 58 | 1 | 5 | 5 | 0.00 | 0.00 |
| 2022 | 58 | 1 | 5 | 5 | 0.00 | 0.00 |
| 2022 | 58 | 1 | 5 | 5 | 0.00 | 0.00 |
| 2022 | 58 | 1 | 5 | 5 | 0.00 | 0.00 |
| 2022 | 58 | 1 | 5 | 5 | 0.00 | 0.00 |
| 2022 | 58 | 2 | 1 | 5 | 6.50 | 0.00 |
| 2022 | 58 | 2 | 1 | 5 | 3.00 | 0.00 |

| | | | | | | |
|---|---|---|---|---|---|---|
| 2022 | 58 | 2 | 1 | 5 | 3.50 | 0.00 |
| 2022 | 58 | 2 | 5 | 5 | 0.00 | 0.00 |
| 2022 | 58 | 2 | 5 | 5 | 0.00 | 0.00 |
| 2022 | 58 | 2 | 5 | 5 | 0.00 | 0.00 |
| 2022 | 58 | 2 | 5 | 5 | 0.00 | 0.00 |
| 2022 | 58 | 2 | 5 | 5 | 0.00 | 0.00 |
| 2022 | 58 | 2 | 5 | 5 | 0.00 | 0.00 |
| 2022 | 58 | 2 | 5 | 5 | 0.00 | 0.00 |
| 2022 | 59 | 1 | 5 | 5 | 0.00 | 0.00 |
| 2022 | 59 | 1 | 5 | 5 | 0.00 | 0.00 |
| 2022 | 59 | 1 | 5 | 5 | 0.00 | 0.00 |
| 2022 | 59 | 1 | 5 | 5 | 0.00 | 0.00 |
| 2022 | 59 | 1 | 5 | 3 | 0.00 | 3.00 |
| 2022 | 59 | 1 | 2 | 3 | 17.00 | 15.00 |
| 2022 | 59 | 1 | 2 | 3 | 15.00 | 4.50 |
| 2022 | 59 | 1 | 2 | 3 | 3.50 | 10.00 |
| 2022 | 59 | 1 | 1 | 2 | 20.50 | 18.00 |
| 2022 | 59 | 1 | 1 | 2 | 14.50 | 20.00 |
| 2022 | 59 | 2 | 5 | 5 | 0.00 | 0.00 |
| 2022 | 59 | 2 | 5 | 5 | 0.00 | 0.00 |
| 2022 | 59 | 2 | 5 | 5 | 0.00 | 0.00 |
| 2022 | 59 | 2 | 5 | 5 | 0.00 | 0.00 |
| 2022 | 59 | 2 | 5 | 5 | 0.00 | 0.00 |
| 2022 | 59 | 2 | 2 | 5 | 4.50 | 0.00 |
| 2022 | 59 | 2 | 1 | 5 | 22.00 | 0.00 |
| 2022 | 59 | 2 | 1 | 4 | 20.50 | 5.00 |
| 2022 | 59 | 2 | 1 | 4 | 30.00 | 11.00 |
| 2022 | 59 | 2 | 1 | 3 | 22.50 | 10.00 |
| 2022 | 60 | 1 | 5 | 5 | 0.00 | 0.00 |
| 2022 | 60 | 1 | 3 | 5 | 4.50 | 0.00 |
| 2022 | 60 | 1 | 1 | 5 | 9.50 | 0.00 |
| 2022 | 60 | 1 | 1 | 2 | 6.20 | 4.00 |
| 2022 | 60 | 1 | 1 | 2 | 0.50 | 6.20 |
| 2022 | 60 | 1 | 1 | 2 | 0.50 | 8.60 |
| 2022 | 60 | 1 | 1 | 2 | 3.50 | 7.30 |
| 2022 | 60 | 1 | 1 | 2 | 4.70 | 2.50 |
| 2022 | 60 | 1 | 1 | 2 | 8.30 | 6.00 |
| 2022 | 60 | 1 | 1 | 2 | 5.10 | 1.50 |
| 2022 | 60 | 2 | 5 | 2 | 0.00 | 0.50 |
| 2022 | 60 | 2 | 5 | 2 | 0.00 | 0.50 |
| 2022 | 60 | 2 | 5 | 4 | 0.00 | 0.50 |
| 2022 | 60 | 2 | 5 | 4 | 0.00 | 0.50 |
| 2022 | 60 | 2 | 3 | 2 | 2.80 | 0.50 |
| 2022 | 60 | 2 | 1 | 2 | 0.50 | 7.00 |
| 2022 | 60 | 2 | 1 | 2 | 4.30 | 4.50 |
| 2022 | 60 | 2 | 1 | 2 | 5.60 | 3.00 |
| 2022 | 60 | 2 | 1 | 2 | 4.40 | 5.20 |
| 2022 | 60 | 2 | 1 | 2 | 6.30 | 3.00 |
| 2022 | 61 | 1 | 5 | 4 | 0.00 | 16.50 |
| 2022 | 61 | 1 | 5 | 5 | 0.00 | 0.00 |
| 2022 | 61 | 1 | 5 | 5 | 0.00 | 0.00 |
| 2022 | 61 | 1 | 5 | 5 | 0.00 | 0.00 |
| 2022 | 61 | 1 | 5 | 5 | 0.00 | 0.00 |
| 2022 | 61 | 1 | 5 | 5 | 0.00 | 0.00 |
| 2022 | 61 | 1 | 5 | 5 | 0.00 | 0.00 |
| 2022 | 61 | 1 | 5 | 5 | 0.00 | 0.00 |
| 2022 | 61 | 1 | 5 | 5 | 0.00 | 0.00 |
| 2022 | 61 | 1 | 5 | 5 | 0.00 | 0.00 |

| | | | | | | |
|---|---|---|---|---|---|---|
| 2022 | 61 | 2 | 5 | 5 | 0.00 | 0.00 |
| 2022 | 61 | 2 | 5 | 5 | 0.00 | 0.00 |
| 2022 | 61 | 2 | 5 | 5 | 0.00 | 0.00 |
| 2022 | 61 | 2 | 5 | 5 | 0.00 | 0.00 |
| 2022 | 61 | 2 | 5 | 5 | 0.00 | 0.00 |
| 2022 | 61 | 2 | 5 | 5 | 0.00 | 0.00 |
| 2022 | 61 | 2 | 5 | 5 | 0.00 | 0.00 |
| 2022 | 61 | 2 | 5 | 5 | 0.00 | 0.00 |
| 2022 | 61 | 2 | 5 | 5 | 0.00 | 0.00 |
| 2022 | 61 | 2 | 5 | 5 | 0.00 | 0.00 |
| 2022 | 62 | 1 | 5 | 2 | 0.00 | 8.30 |
| 2022 | 62 | 1 | 5 | 2 | 0.00 | 7.50 |
| 2022 | 62 | 1 | 5 | 4 | 0.00 | 6.10 |
| 2022 | 62 | 1 | 1 | 4 | 0.50 | 5.20 |
| 2022 | 62 | 1 | 1 | 3 | 11.80 | 4.00 |
| 2022 | 62 | 1 | 1 | 2 | 12.90 | 6.50 |
| 2022 | 62 | 1 | 1 | 5 | 5.60 | 0.00 |
| 2022 | 62 | 1 | 1 | 5 | 8.70 | 0.00 |
| 2022 | 62 | 1 | 1 | 5 | 7.60 | 0.00 |
| 2022 | 62 | 1 | 1 | 5 | 7.30 | 0.00 |
| 2022 | 62 | 2 | 5 | 5 | 0.00 | 0.00 |
| 2022 | 62 | 2 | 5 | 5 | 0.00 | 0.00 |
| 2022 | 62 | 2 | 1 | 5 | 10.60 | 0.00 |
| 2022 | 62 | 2 | 1 | 5 | 10.40 | 0.00 |
| 2022 | 62 | 2 | 1 | 5 | 14.70 | 0.00 |
| 2022 | 62 | 2 | 1 | 2 | 8.60 | 0.50 |
| 2022 | 62 | 2 | 1 | 2 | 8.90 | 7.10 |
| 2022 | 62 | 2 | 1 | 2 | 10.30 | 9.40 |
| 2022 | 62 | 2 | 1 | 2 | 4.30 | 5.10 |
| 2022 | 62 | 2 | 1 | 2 | 6.80 | 0.50 |
| 2022 | 63 | 1 | 5 | 4 | 0.00 | 6.70 |
| 2022 | 63 | 1 | 5 | 4 | 0.00 | 5.40 |
| 2022 | 63 | 1 | 5 | 4 | 0.00 | 2.50 |
| 2022 | 63 | 1 | 5 | 5 | 0.00 | 0.00 |
| 2022 | 63 | 1 | 5 | 5 | 0.00 | 0.00 |
| 2022 | 63 | 1 | 5 | 5 | 0.00 | 0.00 |
| 2022 | 63 | 1 | 5 | 5 | 0.00 | 0.00 |
| 2022 | 63 | 1 | 5 | 5 | 0.00 | 0.00 |
| 2022 | 63 | 1 | 5 | 5 | 0.00 | 0.00 |
| 2022 | 63 | 1 | 5 | 5 | 0.00 | 0.00 |
| 2022 | 63 | 2 | 5 | 5 | 0.00 | 0.00 |
| 2022 | 63 | 2 | 5 | 5 | 0.00 | 0.00 |
| 2022 | 63 | 2 | 5 | 5 | 0.00 | 0.00 |
| 2022 | 63 | 2 | 5 | 5 | 0.00 | 0.00 |
| 2022 | 63 | 2 | 5 | 5 | 0.00 | 0.00 |
| 2022 | 63 | 2 | 5 | 5 | 0.00 | 0.00 |
| 2022 | 63 | 2 | 5 | 5 | 0.00 | 0.00 |
| 2022 | 63 | 2 | 5 | 2 | 0.00 | 7.30 |
| 2022 | 63 | 2 | 5 | 2 | 0.00 | 10.30 |
| 2022 | 63 | 2 | 5 | 2 | 0.00 | 10.10 |
| 2022 | 64 | 1 | 5 | 4 | 0.00 | 10.30 |
| 2022 | 64 | 1 | 5 | 4 | 0.00 | 7.20 |
| 2022 | 64 | 1 | 5 | 5 | 0.00 | 0.00 |
| 2022 | 64 | 1 | 5 | 5 | 0.00 | 0.00 |
| 2022 | 64 | 1 | 5 | 5 | 0.00 | 0.00 |
| 2022 | 64 | 1 | 5 | 5 | 0.00 | 0.00 |
| 2022 | 64 | 1 | 5 | 5 | 0.00 | 0.00 |
| 2022 | 64 | 1 | 5 | 5 | 0.00 | 0.00 |

| | | | | | | |
|---|---|---|---|---|---|---|
| 2022 | 64 | 1 | 5 | 5 | 0.00 | 0.00 |
| 2022 | 64 | 1 | 1 | 5 | 9.50 | 0.00 |
| 2022 | 64 | 2 | 5 | 5 | 0.00 | 0.00 |
| 2022 | 64 | 2 | 5 | 5 | 0.00 | 0.00 |
| 2022 | 64 | 2 | 5 | 5 | 0.00 | 0.00 |
| 2022 | 64 | 2 | 5 | 5 | 0.00 | 0.00 |
| 2022 | 64 | 2 | 5 | 5 | 0.00 | 0.00 |
| 2022 | 64 | 2 | 5 | 5 | 0.00 | 0.00 |
| 2022 | 64 | 2 | 5 | 5 | 0.00 | 0.00 |
| 2022 | 64 | 2 | 5 | 5 | 0.00 | 0.00 |
| 2022 | 64 | 2 | 5 | 5 | 0.00 | 0.00 |
| 2022 | 64 | 2 | 4 | 5 | 8.50 | 0.00 |
| 2022 | 65 | 1 | 1 | 5 | 4.00 | 0.00 |
| 2022 | 65 | 1 | 1 | 5 | 4.80 | 0.00 |
| 2022 | 65 | 1 | 1 | 5 | 10.60 | 0.00 |
| 2022 | 65 | 1 | 5 | 5 | 0.00 | 0.00 |
| 2022 | 65 | 1 | 5 | 5 | 0.00 | 0.00 |
| 2022 | 65 | 1 | 5 | 5 | 0.00 | 0.00 |
| 2022 | 65 | 1 | 5 | 5 | 0.00 | 0.00 |
| 2022 | 65 | 1 | 5 | 5 | 0.00 | 0.00 |
| 2022 | 65 | 1 | 5 | 5 | 0.00 | 0.00 |
| 2022 | 65 | 1 | 5 | 5 | 0.00 | 0.00 |
| 2022 | 65 | 2 | 5 | 5 | 0.00 | 0.00 |
| 2022 | 65 | 2 | 5 | 5 | 0.00 | 0.00 |
| 2022 | 65 | 2 | 5 | 5 | 0.00 | 0.00 |
| 2022 | 65 | 2 | 5 | 5 | 0.00 | 0.00 |
| 2022 | 65 | 2 | 5 | 5 | 0.00 | 0.00 |
| 2022 | 65 | 2 | 5 | 5 | 0.00 | 0.00 |
| 2022 | 65 | 2 | 5 | 5 | 0.00 | 0.00 |
| 2022 | 65 | 2 | 5 | 5 | 0.00 | 0.00 |
| 2022 | 65 | 2 | 5 | 4 | 0.00 | 6.90 |
| 2022 | 65 | 2 | 5 | 4 | 0.00 | 5.80 |
| 2022 | 66 | 1 | 5 | 4 | 0.00 | 8.50 |
| 2022 | 66 | 1 | 5 | 4 | 0.00 | 13.20 |
| 2022 | 66 | 1 | 5 | 5 | 0.00 | 0.00 |
| 2022 | 66 | 1 | 5 | 5 | 0.00 | 0.00 |
| 2022 | 66 | 1 | 1 | 5 | 6.50 | 0.00 |
| 2022 | 66 | 1 | 1 | 5 | 0.50 | 0.00 |
| 2022 | 66 | 1 | 1 | 5 | 6.80 | 0.00 |
| 2022 | 66 | 1 | 3 | 5 | 6.30 | 0.00 |
| 2022 | 66 | 1 | 1 | 5 | 5.10 | 0.00 |
| 2022 | 66 | 1 | 1 | 5 | 11.30 | 0.00 |
| 2022 | 66 | 2 | 1 | 5 | 18.90 | 0.00 |
| 2022 | 66 | 2 | 1 | 5 | 10.30 | 0.00 |
| 2022 | 66 | 2 | 1 | 5 | 10.80 | 0.00 |
| 2022 | 66 | 2 | 1 | 5 | 6.30 | 0.00 |
| 2022 | 66 | 2 | 5 | 5 | 0.00 | 0.00 |
| 2022 | 66 | 2 | 5 | 5 | 0.00 | 0.00 |
| 2022 | 66 | 2 | 5 | 5 | 0.00 | 0.00 |
| 2022 | 66 | 2 | 5 | 5 | 0.00 | 0.00 |
| 2022 | 66 | 2 | 5 | 5 | 0.00 | 0.00 |
| 2022 | 66 | 2 | 5 | 4 | 0.00 | 4.20 |
| 2022 | 67 | 1 | 1 | 5 | 1.50 | 0.00 |
| 2022 | 67 | 1 | 1 | 4 | 1.50 | 0.50 |
| 2022 | 67 | 1 | 1 | 2 | 0.50 | 0.50 |
| 2022 | 67 | 1 | 1 | 2 | 6.10 | 0.50 |
| 2022 | 67 | 1 | 1 | 2 | 4.20 | 0.50 |
| 2022 | 67 | 1 | 5 | 2 | 0.00 | 0.50 |

| | | | | | | |
|---|---|---|---|---|---|---|
| 2022 | 67 | 1 | 5 | 2 | 0.00 | 0.50 |
| 2022 | 67 | 1 | 5 | 2 | 0.00 | 5.50 |
| 2022 | 67 | 1 | 5 | 2 | 0.00 | 4.00 |
| 2022 | 67 | 1 | 5 | 2 | 0.00 | 3.60 |
| 2022 | 67 | 2 | 1 | 2 | 1.50 | 0.50 |
| 2022 | 67 | 2 | 1 | 2 | 1.50 | 0.50 |
| 2022 | 67 | 2 | 1 | 5 | 1.50 | 0.00 |
| 2022 | 67 | 2 | 1 | 5 | 1.50 | 0.00 |
| 2022 | 67 | 2 | 1 | 2 | 1.50 | 4.60 |
| 2022 | 67 | 2 | 1 | 2 | 3.50 | 2.80 |
| 2022 | 67 | 2 | 1 | 2 | 22.60 | 2.70 |
| 2022 | 67 | 2 | 1 | 2 | 6.50 | 3.70 |
| 2022 | 67 | 2 | 1 | 2 | 4.70 | 2.50 |
| 2022 | 67 | 2 | 1 | 2 | 3.60 | 0.50 |
| 2022 | 68 | 1 | 1 | 2 | 4.00 | 1.50 |
| 2022 | 68 | 1 | 1 | 2 | 5.50 | 0.50 |
| 2022 | 68 | 1 | 1 | 2 | 4.70 | 5.20 |
| 2022 | 68 | 1 | 1 | 2 | 4.60 | 6.50 |
| 2022 | 68 | 1 | 1 | 2 | 3.80 | 4.50 |
| 2022 | 68 | 1 | 1 | 2 | 7.50 | 2.60 |
| 2022 | 68 | 1 | 1 | 2 | 6.30 | 4.80 |
| 2022 | 68 | 1 | 1 | 2 | 5.50 | 5.00 |
| 2022 | 68 | 1 | 1 | 2 | 8.50 | 4.60 |
| 2022 | 68 | 1 | 5 | 2 | 0.00 | 7.00 |
| 2022 | 68 | 2 | 1 | 5 | 0.50 | 0.00 |
| 2022 | 68 | 2 | 1 | 5 | 6.50 | 0.00 |
| 2022 | 68 | 2 | 1 | 5 | 7.40 | 0.00 |
| 2022 | 68 | 2 | 1 | 2 | 8.10 | 2.90 |
| 2022 | 68 | 2 | 1 | 2 | 7.00 | 5.40 |
| 2022 | 68 | 2 | 1 | 2 | 8.20 | 3.50 |
| 2022 | 68 | 2 | 1 | 2 | 3.40 | 3.00 |
| 2022 | 68 | 2 | 5 | 2 | 0.00 | 1.50 |
| 2022 | 68 | 2 | 5 | 2 | 0.00 | 2.50 |
| 2022 | 68 | 2 | 5 | 2 | 0.00 | 0.50 |
| 2022 | 69 | 1 | 1 | 2 | 8.50 | 0.50 |
| 2022 | 69 | 1 | 1 | 2 | 2.50 | 4.50 |
| 2022 | 69 | 1 | 1 | 2 | 3.70 | 5.20 |
| 2022 | 69 | 1 | 1 | 2 | 1.50 | 2.60 |
| 2022 | 69 | 1 | 1 | 2 | 4.50 | 7.00 |
| 2022 | 69 | 1 | 1 | 2 | 8.20 | 4.90 |
| 2022 | 69 | 1 | 1 | 2 | 0.50 | 6.00 |
| 2022 | 69 | 1 | 1 | 2 | 5.70 | 6.20 |
| 2022 | 69 | 1 | 1 | 2 | 3.20 | 5.80 |
| 2022 | 69 | 1 | 5 | 2 | 0.00 | 4.50 |
| 2022 | 69 | 2 | 5 | 2 | 0.00 | 0.50 |
| 2022 | 69 | 2 | 1 | 2 | 6.50 | 0.50 |
| 2022 | 69 | 2 | 1 | 2 | 7.90 | 8.50 |
| 2022 | 69 | 2 | 1 | 2 | 6.60 | 7.00 |
| 2022 | 69 | 2 | 1 | 2 | 5.40 | 9.30 |
| 2022 | 69 | 2 | 1 | 2 | 3.50 | 9.60 |
| 2022 | 69 | 2 | 1 | 2 | 5.20 | 7.10 |
| 2022 | 69 | 2 | 1 | 2 | 2.50 | 3.50 |
| 2022 | 69 | 2 | 1 | 2 | 6.80 | 2.50 |
| 2022 | 69 | 2 | 1 | 2 | 7.10 | 1.00 |
| 2022 | 70 | 1 | 5 | 5 | 0.00 | 0.00 |
| 2022 | 70 | 1 | 5 | 4 | 0.00 | 4.50 |
| 2022 | 70 | 1 | 5 | 4 | 0.00 | 5.00 |
| 2022 | 70 | 1 | 1 | 2 | 9.00 | 7.00 |

| | | | | | | |
|---|---|---|---|---|---|---|
| 2022 | 70 | 1 | 1 | 2 | 1.50 | 5.50 |
| 2022 | 70 | 1 | 1 | 2 | 6.40 | 8.00 |
| 2022 | 70 | 1 | 1 | 2 | 5.10 | 2.50 |
| 2022 | 70 | 1 | 1 | 2 | 4.50 | 0.50 |
| 2022 | 70 | 1 | 1 | 2 | 8.00 | 4.00 |
| 2022 | 70 | 1 | 1 | 2 | 6.70 | 3.50 |
| 2022 | 70 | 2 | 5 | 5 | 0.00 | 0.00 |
| 2022 | 70 | 2 | 5 | 5 | 0.00 | 0.00 |
| 2022 | 70 | 2 | 1 | 5 | 0.50 | 0.00 |
| 2022 | 70 | 2 | 1 | 2 | 0.50 | 1.50 |
| 2022 | 70 | 2 | 1 | 2 | 0.50 | 2.50 |
| 2022 | 70 | 2 | 1 | 2 | 3.60 | 5.60 |
| 2022 | 70 | 2 | 1 | 2 | 7.10 | 4.70 |
| 2022 | 70 | 2 | 1 | 2 | 5.60 | 6.30 |
| 2022 | 70 | 2 | 1 | 2 | 6.20 | 3.80 |
| 2022 | 70 | 2 | 1 | 2 | 4.50 | 6.60 |
| | 71 | 1 | 5 | 5 | 0.00 | 0.00 |
| | 71 | 1 | 1 | 5 | 22.70 | 0.00 |
| | 71 | 1 | 1 | 5 | 20.10 | 0.00 |
| | 71 | 1 | 1 | 3 | 19.10 | 3.10 |
| | 71 | 1 | 1 | 2 | 18.60 | 3.50 |
| | 71 | 1 | 1 | 2 | 18.10 | 9.10 |
| | 71 | 1 | 1 | 2 | 25.90 | 5.50 |
| | 71 | 1 | 1 | 2 | 17.30 | 7.60 |
| | 71 | 1 | 1 | 2 | 16.10 | 6.50 |
| | 71 | 1 | 1 | 2 | 5.80 | 5.10 |
| | 71 | 2 | 1 | 5 | 7.00 | 0.00 |
| | 71 | 2 | 1 | 2 | 16.40 | 9.50 |
| | 71 | 2 | 1 | 2 | 16.50 | 8.50 |
| | 71 | 2 | 1 | 2 | 19.00 | 6.10 |
| | 71 | 2 | 1 | 2 | 14.50 | 8.60 |
| | 71 | 2 | 1 | 2 | 13.60 | 9.10 |
| | 71 | 2 | 1 | 2 | 10.10 | 7.40 |
| | 71 | 2 | 1 | 2 | 10.50 | 6.20 |
| | 71 | 2 | 1 | 2 | 11.20 | 4.10 |
| | 71 | 2 | 1 | 2 | 10.80 | 4.00 |
| | 72 | 1 | 1 | 2 | 29.50 | 10.50 |
| | 72 | 1 | 1 | 2 | 21.70 | 8.50 |
| | 72 | 1 | 1 | 2 | 21.30 | 9.20 |
| | 72 | 1 | 1 | 2 | 25.50 | 11.40 |
| | 72 | 1 | 1 | 2 | 29.20 | 10.50 |
| | 72 | 1 | 1 | 2 | 18.30 | 15.20 |
| | 72 | 1 | 1 | 2 | 8.70 | 9.40 |
| | 72 | 1 | 1 | 2 | 18.90 | 10.00 |
| | 72 | 1 | 1 | 2 | 19.30 | 9.50 |
| | 72 | 1 | 1 | 2 | 18.50 | 5.00 |
| | 72 | 2 | 1 | 3 | 15.30 | 8.50 |
| | 72 | 2 | 1 | 3 | 32.00 | 21.70 |
| | 72 | 2 | 1 | 2 | 17.20 | 23.50 |
| | 72 | 2 | 1 | 2 | 24.50 | 12.00 |
| | 72 | 2 | 1 | 2 | 15.20 | 11.20 |
| | 72 | 2 | 1 | 2 | 30.70 | 12.50 |
| | 72 | 2 | 1 | 2 | 29.50 | 9.00 |
| | 72 | 2 | 1 | 2 | 36.50 | 25.00 |
| | 72 | 2 | 1 | 2 | 13.50 | 25.30 |
| | 72 | 2 | 1 | 2 | 14.80 | 22.60 |
| | 73 | 1 | 5 | 5 | 0.00 | 0.00 |
| | 73 | 1 | 5 | 5 | 0.00 | 0.00 |

| | | | | | |
|---|---|---|---|---|---|
| 73 | 1 | 5 | 5 | 0.00 | 0.00 |
| 73 | 1 | 5 | 5 | 0.00 | 0.00 |
| 73 | 1 | 3 | 5 | 3.50 | 0.00 |
| 73 | 1 | 1 | 2 | 10.50 | 0.50 |
| 73 | 1 | 1 | 2 | 13.30 | 0.50 |
| 73 | 1 | 1 | 2 | 10.10 | 0.50 |
| 73 | 1 | 1 | 2 | 12.90 | 4.00 |
| 73 | 1 | 1 | 2 | 0.50 | 2.60 |
| 73 | 2 | 5 | 5 | 0.00 | 0.00 |
| 73 | 2 | 5 | 5 | 0.00 | 0.00 |
| 73 | 2 | 5 | 5 | 0.00 | 0.00 |
| 73 | 2 | 1 | 5 | 4.30 | 0.00 |
| 73 | 2 | 1 | 5 | 0.50 | 0.00 |
| 73 | 2 | 1 | 5 | 9.50 | 0.00 |
| 73 | 2 | 1 | 2 | 7.00 | 0.50 |
| 73 | 2 | 1 | 2 | 2.50 | 0.50 |
| 73 | 2 | 1 | 2 | 2.50 | 2.50 |
| 73 | 2 | 1 | 2 | 2.00 | 4.20 |
| 74 | 1 | 5 | 5 | 0.00 | 0.00 |
| 74 | 1 | 5 | 5 | 0.00 | 0.00 |
| 74 | 1 | 1 | 5 | 1.50 | 0.00 |
| 74 | 1 | 1 | 5 | 4.30 | 0.00 |
| 74 | 1 | 1 | 5 | 12.20 | 0.00 |
| 74 | 1 | 1 | 5 | 5.40 | 0.00 |
| 74 | 1 | 1 | 5 | 16.60 | 0.00 |
| 74 | 1 | 1 | 2 | 3.50 | 0.50 |
| 74 | 1 | 1 | 2 | 6.20 | 6.10 |
| 74 | 1 | 1 | 2 | 5.40 | 7.30 |
| 74 | 2 | 5 | 5 | 0.00 | 0.00 |
| 74 | 2 | 5 | 5 | 0.00 | 0.00 |
| 74 | 2 | 5 | 5 | 0.00 | 0.00 |
| 74 | 2 | 5 | 5 | 0.00 | 0.00 |
| 74 | 2 | 1 | 5 | 0.50 | 0.00 |
| 74 | 2 | 1 | 2 | 0.50 | 0.50 |
| 74 | 2 | 1 | 2 | 8.20 | 4.10 |
| 74 | 2 | 1 | 2 | 6.90 | 2.50 |
| 74 | 2 | 1 | 2 | 11.50 | 5.00 |
| 74 | 2 | 1 | 2 | 2.70 | 6.40 |
| 75 | 1 | 5 | 5 | 0.00 | 0.00 |
| 75 | 1 | 5 | 5 | 0.00 | 0.00 |
| 75 | 1 | 1 | 2 | 13.40 | 6.50 |
| 75 | 1 | 1 | 2 | 18.20 | 5.50 |
| 75 | 1 | 1 | 2 | 10.10 | 4.20 |
| 75 | 1 | 1 | 2 | 11.50 | 0.50 |
| 75 | 1 | 1 | 2 | 5.40 | 0.50 |
| 75 | 1 | 1 | 2 | 9.20 | 11.40 |
| 75 | 1 | 1 | 2 | 10.50 | 3.10 |
| 75 | 1 | 1 | 2 | 6.40 | 9.00 |
| 75 | 2 | 5 | 5 | 0.00 | 0.00 |
| 75 | 2 | 5 | 5 | 0.00 | 0.00 |
| 75 | 2 | 5 | 5 | 0.00 | 0.00 |
| 75 | 2 | 5 | 5 | 0.00 | 0.00 |
| 75 | 2 | 5 | 4 | 0.00 | 0.50 |
| 75 | 2 | 1 | 2 | 0.50 | 15.00 |
| 75 | 2 | 1 | 2 | 18.70 | 10.50 |
| 75 | 2 | 1 | 2 | 3.60 | 12.30 |
| 75 | 2 | 1 | 2 | 2.10 | 2.50 |
| 75 | 2 | 1 | 2 | 13.50 | 2.50 |

| | | | | | |
|---|---|---|---|---|---|
| 76 | 1 | 1 | 5 | 0.50 | 0.00 |
| 76 | 1 | 1 | 5 | 0.50 | 0.00 |
| 76 | 1 | 1 | 3 | 9.80 | 0.50 |
| 76 | 1 | 1 | 5 | 8.20 | 0.00 |
| 76 | 1 | 1 | 1 | 2.90 | 6.70 |
| 76 | 1 | 1 | 1 | 5.90 | 4.00 |
| 76 | 1 | 1 | 1 | 6.80 | 5.30 |
| 76 | 1 | 1 | 1 | 7.50 | 6.60 |
| 76 | 1 | 1 | 1 | 3.90 | 2.40 |
| 76 | 1 | 1 | 1 | 8.00 | 5.30 |
| 76 | 2 | 3 | 5 | 4.10 | 0.00 |
| 76 | 2 | 1 | 2 | 7.10 | 1.90 |
| 76 | 2 | 1 | 2 | 5.40 | 5.20 |
| 76 | 2 | 1 | 2 | 4.50 | 4.30 |
| 76 | 2 | 1 | 2 | 2.30 | 5.20 |
| 76 | 2 | 1 | 2 | 1.30 | 2.10 |
| 76 | 2 | 1 | 2 | 6.40 | 3.50 |
| 76 | 2 | 1 | 2 | 5.50 | 9.40 |
| 76 | 2 | 1 | 5 | 3.50 | 0.00 |
| 76 | 2 | 1 | 5 | 3.20 | 0.00 |
| 77 | 1 | 5 | 5 | 0.00 | 0.00 |
| 77 | 1 | 5 | 5 | 0.00 | 0.00 |
| 77 | 1 | 5 | 5 | 0.00 | 0.00 |
| 77 | 1 | 5 | 5 | 0.00 | 0.00 |
| 77 | 1 | 5 | 5 | 0.00 | 0.00 |
| 77 | 1 | 3 | 5 | 0.50 | 0.00 |
| 77 | 1 | 3 | 5 | 6.20 | 0.00 |
| 77 | 1 | 1 | 5 | 0.50 | 0.00 |
| 77 | 1 | 1 | 5 | 1.50 | 0.00 |
| 77 | 1 | 1 | 2 | 3.90 | 0.50 |
| 77 | 2 | 5 | 5 | 0.00 | 0.00 |
| 77 | 2 | 5 | 5 | 0.00 | 0.00 |
| 77 | 2 | 5 | 5 | 0.00 | 0.00 |
| 77 | 2 | 5 | 5 | 0.00 | 0.00 |
| 77 | 2 | 5 | 5 | 0.00 | 0.00 |
| 77 | 2 | 5 | 5 | 0.00 | 0.00 |
| 77 | 2 | 5 | 5 | 0.00 | 0.00 |
| 77 | 2 | 5 | 5 | 0.00 | 0.00 |
| 77 | 2 | 1 | 5 | 3.20 | 0.00 |
| 77 | 2 | 1 | 2 | 6.80 | 4.20 |
| 78 | 1 | 5 | 5 | 0.00 | 0.00 |
| 78 | 1 | 5 | 5 | 0.00 | 0.00 |
| 78 | 1 | 1 | 5 | 0.50 | 0.00 |
| 78 | 1 | 1 | 5 | 1.50 | 0.00 |
| 78 | 1 | 1 | 5 | 2.90 | 0.00 |
| 78 | 1 | 1 | 5 | 7.60 | 0.00 |
| 78 | 1 | 1 | 5 | 6.90 | 0.00 |
| 78 | 1 | 1 | 2 | 4.40 | 0.50 |
| 78 | 1 | 1 | 2 | 7.90 | 0.50 |
| 78 | 1 | 1 | 3 | 6.40 | 0.50 |
| 78 | 2 | 5 | 5 | 0.00 | 0.00 |
| 78 | 2 | 5 | 5 | 0.00 | 0.00 |
| 78 | 2 | 5 | 5 | 0.00 | 0.00 |
| 78 | 2 | 5 | 5 | 0.00 | 0.00 |
| 78 | 2 | 5 | 5 | 0.00 | 0.00 |
| 78 | 2 | 3 | 2 | 4.30 | 0.50 |
| 78 | 2 | 1 | 2 | 0.50 | 0.50 |
| 78 | 2 | 1 | 2 | 0.50 | 0.50 |

| | | | | | |
|---|---|---|---|---|---|
| 78 | 2 | 1 | 2 | 2.60 | 5.10 |
| 78 | 2 | 1 | 2 | 7.30 | 4.50 |
| 79 | 1 | 5 | 5 | 0.00 | 0.00 |
| 79 | 1 | 5 | 5 | 0.00 | 0.00 |
| 79 | 1 | 5 | 5 | 0.00 | 0.00 |
| 79 | 1 | 5 | 5 | 0.00 | 0.00 |
| 79 | 1 | 5 | 5 | 0.00 | 0.00 |
| 79 | 1 | 1 | 2 | 0.50 | 0.50 |
| 79 | 1 | 1 | 2 | 0.50 | 0.50 |
| 79 | 1 | 1 | 2 | 7.40 | 2.50 |
| 79 | 1 | 1 | 4 | 6.60 | 1.30 |
| 79 | 1 | 1 | 4 | 1.90 | 3.50 |
| 79 | 2 | 5 | 5 | 0.00 | 0.00 |
| 79 | 2 | 5 | 5 | 0.00 | 0.00 |
| 79 | 2 | 5 | 5 | 0.00 | 0.00 |
| 79 | 2 | 5 | 5 | 0.00 | 0.00 |
| 79 | 2 | 1 | 5 | 3.60 | 0.00 |
| 79 | 2 | 1 | 2 | 1.90 | 2.80 |
| 79 | 2 | 1 | 2 | 3.90 | 1.20 |
| 79 | 2 | 1 | 2 | 7.10 | 3.10 |
| 79 | 2 | 1 | 2 | 2.30 | 3.40 |
| 79 | 2 | 1 | 2 | 2.20 | 4.50 |
| 80 | 1 | 5 | 5 | 0.00 | 0.00 |
| 80 | 1 | 5 | 5 | 0.00 | 0.00 |
| 80 | 1 | 1 | 5 | 0.50 | 0.00 |
| 80 | 1 | 1 | 4 | 19.50 | 6.30 |
| 80 | 1 | 3 | 4 | 11.20 | 3.50 |
| 80 | 1 | 1 | 2 | 14.10 | 8.50 |
| 80 | 1 | 1 | 2 | 10.20 | 6.10 |
| 80 | 1 | 1 | 2 | 9.70 | 4.70 |
| 80 | 1 | 1 | 2 | 6.50 | 1.00 |
| 80 | 1 | 1 | 2 | 4.30 | 0.50 |
| 80 | 2 | 5 | 4 | 0.00 | 0.50 |
| 80 | 2 | 5 | 4 | 0.00 | 0.50 |
| 80 | 2 | 3 | 4 | 5.00 | 6.50 |
| 80 | 2 | 1 | 4 | 12.60 | 13.40 |
| 80 | 2 | 1 | 2 | 15.10 | 0.50 |
| 80 | 2 | 1 | 2 | 12.20 | 15.50 |
| 80 | 2 | 1 | 2 | 8.60 | 10.70 |
| 80 | 2 | 1 | 2 | 8.00 | 9.50 |
| 80 | 2 | 1 | 2 | 8.20 | 8.40 |
| 80 | 2 | 1 | 2 | 6.50 | 0.50 |
| 81 | 1 | 5 | 5 | 0.00 | 0.00 |
| 81 | 1 | 5 | 5 | 0.00 | 0.00 |
| 81 | 1 | 5 | 5 | 0.00 | 0.00 |
| 81 | 1 | 5 | 5 | 0.00 | 0.00 |
| 81 | 1 | 5 | 5 | 0.00 | 0.00 |
| 81 | 1 | 5 | 5 | 0.00 | 0.00 |
| 81 | 1 | 5 | 5 | 0.00 | 0.00 |
| 81 | 1 | 5 | 5 | 0.00 | 0.00 |
| 81 | 1 | 5 | 2 | 0.00 | 4.50 |
| 81 | 1 | 5 | 2 | 0.00 | 7.60 |
| 81 | 2 | 5 | 5 | 0.00 | 0.00 |
| 81 | 2 | 5 | 5 | 0.00 | 0.00 |
| 81 | 2 | 5 | 5 | 0.00 | 0.00 |
| 81 | 2 | 5 | 5 | 0.00 | 0.00 |
| 81 | 2 | 5 | 5 | 0.00 | 0.00 |
| 81 | 2 | 5 | 5 | 0.00 | 0.00 |

| | | | | | | |
|---|---|---|---|---|---|---|
| | 81 | 2 | 5 | 5 | 0.00 | 0.00 |
| | 81 | 2 | 5 | 5 | 0.00 | 0.00 |
| | 81 | 2 | 5 | 2 | 0.00 | 3.60 |
| | 81 | 2 | 5 | 2 | 0.00 | 3.90 |
| | 82 | 1 | 5 | 5 | 0.00 | 0.00 |
| | 82 | 1 | 5 | 5 | 0.00 | 0.00 |
| | 82 | 1 | 5 | 5 | 0.00 | 0.00 |
| | 82 | 1 | 1 | 5 | 0.50 | 0.00 |
| | 82 | 1 | 1 | 5 | 9.50 | 0.00 |
| | 82 | 1 | 1 | 2 | 3.70 | 0.50 |
| | 82 | 1 | 1 | 2 | 3.60 | 5.00 |
| | 82 | 1 | 1 | 2 | 3.20 | 7.80 |
| | 82 | 1 | 1 | 2 | 10.40 | 6.60 |
| | 82 | 1 | 1 | 2 | 5.40 | 6.30 |
| | 82 | 2 | 5 | 5 | 0.00 | 0.00 |
| | 82 | 2 | 5 | 5 | 0.00 | 0.00 |
| | 82 | 2 | 3 | 2 | 6.40 | 1.20 |
| | 82 | 2 | 1 | 2 | 0.50 | 0.50 |
| | 82 | 2 | 1 | 2 | 0.50 | 0.50 |
| | 82 | 2 | 1 | 2 | 0.50 | 3.60 |
| | 82 | 2 | 1 | 2 | 4.60 | 4.50 |
| | 82 | 2 | 1 | 2 | 4.30 | 4.00 |
| | 82 | 2 | 1 | 2 | 7.90 | 10.40 |
| | 82 | 2 | 1 | 2 | 8.00 | 3.60 |
| 2023 | Mp705 | 1 | 5 | 5 | 0.00 | 0.00 |
| 2023 | Mp705 | 1 | 1 | 1 | 15.60 | 19.40 |
| 2023 | Mp705 | 1 | 1 | 1 | 17.40 | 20.60 |
| 2023 | Mp705 | 1 | 1 | 1 | 16.20 | 18.30 |
| 2023 | Mp705 | 1 | 1 | 1 | 2.20 | 18.70 |
| 2023 | Mp705 | 1 | 1 | 1 | 20.70 | 20.50 |
| 2023 | Mp705 | 1 | 1 | 1 | 25.40 | 20.00 |
| 2023 | Mp705 | 1 | 1 | 1 | 21.00 | 18.40 |
| 2023 | Mp705 | 1 | 1 | 1 | 20.80 | 19.60 |
| 2023 | Mp705 | 1 | 1 | 1 | 24.90 | 18.00 |
| 2023 | Mp705 | 2 | 1 | 2 | 19.20 | 18.40 |
| 2023 | Mp705 | 2 | 1 | 2 | 10.60 | 19.60 |
| 2023 | Mp705 | 2 | 1 | 2 | 20.10 | 18.00 |
| 2023 | Mp705 | 2 | 1 | 2 | 21.70 | 18.80 |
| 2023 | Mp705 | 2 | 1 | 2 | 19.40 | 21.80 |
| 2023 | Mp705 | 2 | 1 | 1 | 19.50 | 12.50 |
| 2023 | Mp705 | 2 | 1 | 1 | 18.00 | 22.30 |
| 2023 | Mp705 | 2 | 1 | 1 | 15.60 | 19.60 |
| 2023 | Mp705 | 2 | 1 | 1 | 17.00 | 25.40 |
| 2023 | Mp705 | 2 | 1 | 1 | 20.40 | 19.40 |
| 2023 | W22 BRINK | 1 | 5 | 1 | 0.00 | 9.30 |
| 2023 | W22 BRINK | 1 | 1 | 1 | 22.10 | 10.90 |
| 2023 | W22 BRINK | 1 | 1 | 1 | 18.20 | 7.00 |
| 2023 | W22 BRINK | 1 | 1 | 1 | 11.40 | 5.00 |
| 2023 | W22 BRINK | 1 | 1 | 1 | 9.70 | 8.50 |
| 2023 | W22 BRINK | 1 | 1 | 1 | 19.60 | 15.50 |
| 2023 | W22 BRINK | 1 | 1 | 1 | 8.20 | 11.50 |
| 2023 | W22 BRINK | 1 | 1 | 1 | 12.50 | 9.50 |
| 2023 | W22 BRINK | 1 | 1 | 1 | 13.40 | 9.80 |
| 2023 | W22 BRINK | 1 | 1 | 5 | 18.20 | 0.00 |
| 2023 | W22 BRINK | 2 | 1 | 2 | 16.40 | 12.00 |
| 2023 | W22 BRINK | 2 | 1 | 2 | 12.70 | 20.40 |
| 2023 | W22 BRINK | 2 | 1 | 2 | 15.20 | 16.20 |
| 2023 | W22 BRINK | 2 | 1 | 1 | 14.70 | 11.00 |

| | | | | | | |
|---|---|---|---|---|---|---|
| 2023 | W22 BRINK | 2 | 1 | 1 | 11.90 | 18.30 |
| 2023 | W22 BRINK | 2 | 1 | 1 | 14.30 | 16.00 |
| 2023 | W22 BRINK | 2 | 1 | 1 | 17.90 | 6.20 |
| 2023 | W22 BRINK | 2 | 1 | 1 | 13.20 | 6.50 |
| 2023 | W22 BRINK | 2 | 1 | 1 | 8.00 | 13.20 |
| 2023 | W22 BRINK | 2 | 1 | 1 | 9.40 | 14.40 |
| 2023 | Hi53 | 1 | 5 | 5 | 0.00 | 0.00 |
| 2023 | Hi53 | 1 | 3 | 1 | 5.20 | 4.00 |
| 2023 | Hi53 | 1 | 3 | 1 | 3.50 | 8.60 |
| 2023 | Hi53 | 1 | 1 | 1 | 13.60 | 10.50 |
| 2023 | Hi53 | 1 | 1 | 1 | 12.10 | 5.90 |
| 2023 | Hi53 | 1 | 1 | 1 | 16.70 | 10.50 |
| 2023 | Hi53 | 1 | 1 | 5 | 12.50 | 0.00 |
| 2023 | Hi53 | 1 | 1 | 1 | 10.20 | 9.50 |
| 2023 | Hi53 | 1 | 1 | 1 | 8.40 | 6.00 |
| 2023 | Hi53 | 1 | 1 | 1 | 16.10 | 2.50 |
| 2023 | Hi53 | 2 | 1 | 5 | 12.60 | 0.00 |
| 2023 | Hi53 | 2 | 1 | 5 | 12.80 | 0.00 |
| 2023 | Hi53 | 2 | 1 | 3 | 10.20 | 7.70 |
| 2023 | Hi53 | 2 | 1 | 3 | 12.10 | 3.00 |
| 2023 | Hi53 | 2 | 1 | 3 | 13.00 | 9.20 |
| 2023 | Hi53 | 2 | 1 | 3 | 8.50 | 12.80 |
| 2023 | Hi53 | 2 | 1 | 3 | 12.70 | 3.50 |
| 2023 | Hi53 | 2 | 1 | 3 | 7.20 | 5.40 |
| 2023 | Hi53 | 2 | 1 | 3 | 9.40 | 6.00 |
| 2023 | Hi53 | 2 | 1 | 3 | 11.20 | 8.50 |
| 2023 | T143 | 1 | 5 | 5 | 0.00 | 0.00 |
| 2023 | T143 | 1 | 1 | 5 | 11.50 | 0.00 |
| 2023 | T143 | 1 | 1 | 5 | 13.40 | 0.00 |
| 2023 | T143 | 1 | 1 | 5 | 15.60 | 0.00 |
| 2023 | T143 | 1 | 1 | 2 | 11.20 | 16.60 |
| 2023 | T143 | 1 | 1 | 2 | 16.50 | 7.80 |
| 2023 | T143 | 1 | 1 | 1 | 10.70 | 12.70 |
| 2023 | T143 | 1 | 1 | 1 | 6.20 | 12.50 |
| 2023 | T143 | 1 | 1 | 1 | 3.30 | 14.00 |
| 2023 | T143 | 1 | 1 | 1 | 5.20 | 10.70 |
| 2023 | T143 | 2 | 5 | 1 | 0.00 | 16.40 |
| 2023 | T143 | 2 | 1 | 1 | 14.20 | 12.50 |
| 2023 | T143 | 2 | 1 | 1 | 9.40 | 16.20 |
| 2023 | T143 | 2 | 1 | 1 | 5.80 | 10.00 |
| 2023 | T143 | 2 | 1 | 1 | 13.90 | 14.00 |
| 2023 | T143 | 2 | 1 | 1 | 10.50 | 4.50 |
| 2023 | T143 | 2 | 1 | 1 | 14.30 | 1.00 |
| 2023 | T143 | 2 | 1 | 1 | 7.10 | 3.60 |
| 2023 | T143 | 2 | 1 | 1 | 5.60 | 5.90 |
| 2023 | T143 | 2 | 1 | 2 | 12.50 | 8.80 |
| 2023 | TSU-CHIAO-HSI-WU 102 | 1 | 1 | 2 | 10.50 | 10.00 |
| 2023 | TSU-CHIAO-HSI-WU 102 | 1 | 1 | 2 | 13.00 | 11.40 |
| 2023 | TSU-CHIAO-HSI-WU 102 | 1 | 1 | 2 | 12.50 | 16.20 |
| 2023 | TSU-CHIAO-HSI-WU 102 | 1 | 1 | 2 | 15.00 | 5.50 |
| 2023 | TSU-CHIAO-HSI-WU 102 | 1 | 1 | 2 | 13.10 | 14.50 |
| 2023 | TSU-CHIAO-HSI-WU 102 | 1 | 1 | 1 | 14.30 | 9.20 |
| 2023 | TSU-CHIAO-HSI-WU 102 | 1 | 1 | 1 | 14.00 | 15.70 |
| 2023 | TSU-CHIAO-HSI-WU 102 | 1 | 1 | 1 | 13.50 | 10.20 |
| 2023 | TSU-CHIAO-HSI-WU 102 | 1 | 1 | 1 | 16.70 | 3.70 |
| 2023 | TSU-CHIAO-HSI-WU 102 | 1 | 1 | 1 | 12.60 | 10.70 |
| 2023 | TSU-CHIAO-HSI-WU 102 | 2 | 1 | 2 | 15.30 | 6.70 |
| 2023 | TSU-CHIAO-HSI-WU 102 | 2 | 1 | 2 | 12.50 | 6.50 |

| | | | | | | |
|---|---|---|---|---|---|---|
| 2023 | TSU-CHIAO-HSI-WU 102 | 2 | 1 | 2 | 14.20 | 10.50 |
| 2023 | TSU-CHIAO-HSI-WU 102 | 2 | 1 | 2 | 15.30 | 11.00 |
| 2023 | TSU-CHIAO-HSI-WU 102 | 2 | 1 | 2 | 12.70 | 9.50 |
| 2023 | TSU-CHIAO-HSI-WU 102 | 2 | 1 | 2 | 15.60 | 3.00 |
| 2023 | TSU-CHIAO-HSI-WU 102 | 2 | 1 | 1 | 13.00 | 11.50 |
| 2023 | TSU-CHIAO-HSI-WU 102 | 2 | 1 | 1 | 18.60 | 11.00 |
| 2023 | TSU-CHIAO-HSI-WU 102 | 2 | 1 | 1 | 16.80 | 7.00 |
| 2023 | TSU-CHIAO-HSI-WU 102 | 2 | 1 | 1 | 14.70 | 7.60 |
| 2023 | INBRED 39-1546 | 1 | 3 | 5 | 5.20 | 0.00 |
| 2023 | INBRED 39-1546 | 1 | 3 | 4 | 10.00 | 9.00 |
| 2023 | INBRED 39-1546 | 1 | 1 | 2 | 11.50 | 12.50 |
| 2023 | INBRED 39-1546 | 1 | 1 | 2 | 4.60 | 12.50 |
| 2023 | INBRED 39-1546 | 1 | 1 | 4 | 21.90 | 16.50 |
| 2023 | INBRED 39-1546 | 1 | 1 | 2 | 12.50 | 7.70 |
| 2023 | INBRED 39-1546 | 1 | 1 | 2 | 13.70 | 8.50 |
| 2023 | INBRED 39-1546 | 1 | 1 | 2 | 11.40 | 3.00 |
| 2023 | INBRED 39-1546 | 1 | 1 | 2 | 13.80 | 6.50 |
| 2023 | INBRED 39-1546 | 1 | 1 | 2 | 5.60 | 3.50 |
| 2023 | INBRED 39-1546 | 2 | 1 | 5 | 8.60 | 0.00 |
| 2023 | INBRED 39-1546 | 2 | 1 | 1 | 11.30 | 0.50 |
| 2023 | INBRED 39-1546 | 2 | 1 | 1 | 19.50 | 5.60 |
| 2023 | INBRED 39-1546 | 2 | 1 | 1 | 17.20 | 10.50 |
| 2023 | INBRED 39-1546 | 2 | 1 | 1 | 12.40 | 9.30 |
| 2023 | INBRED 39-1546 | 2 | 1 | 1 | 18.00 | 9.00 |
| 2023 | INBRED 39-1546 | 2 | 1 | 1 | 15.50 | 11.60 |
| 2023 | INBRED 39-1546 | 2 | 1 | 1 | 10.10 | 13.20 |
| 2023 | INBRED 39-1546 | 2 | 1 | 1 | 11.20 | 17.10 |
| 2023 | INBRED 39-1546 | 2 | 1 | 1 | 9.50 | 11.70 |
| 2023 | CE-777 | 1 | 1 | 2 | 1.00 | 4.00 |
| 2023 | CE-777 | 1 | 1 | 1 | 4.50 | 6.50 |
| 2023 | CE-777 | 1 | 1 | 2 | 12.40 | 15.00 |
| 2023 | CE-777 | 1 | 1 | 1 | 10.60 | 1.50 |
| 2023 | CE-777 | 1 | 1 | 2 | 7.50 | 20.20 |
| 2023 | CE-777 | 1 | 1 | 1 | 3.00 | 10.30 |
| 2023 | CE-777 | 1 | 1 | 1 | 8.50 | 11.50 |
| 2023 | CE-777 | 1 | 1 | 1 | 6.40 | 17.80 |
| 2023 | CE-777 | 1 | 1 | 1 | 5.20 | 8.00 |
| 2023 | CE-777 | 1 | 1 | 1 | 6.50 | 11.00 |
| 2023 | CE-777 | 2 | 1 | 1 | 8.20 | 12.00 |
| 2023 | CE-777 | 2 | 1 | 1 | 9.60 | 5.80 |
| 2023 | CE-777 | 2 | 1 | 1 | 5.90 | 6.50 |
| 2023 | CE-777 | 2 | 1 | 1 | 8.30 | 9.00 |
| 2023 | CE-777 | 2 | 1 | 1 | 7.60 | 10.80 |
| 2023 | CE-777 | 2 | 1 | 1 | 9.90 | 10.60 |
| 2023 | CE-777 | 2 | 1 | 1 | 8.20 | 9.70 |
| 2023 | CE-777 | 2 | 1 | 1 | 1.50 | 12.50 |
| 2023 | CE-777 | 2 | 1 | 1 | 2.60 | 6.40 |
| 2023 | CE-777 | 2 | 1 | 2 | 6.80 | 5.50 |
| 2023 | CML182 | 1 | 1 | 5 | 0.50 | 0.00 |
| 2023 | CML182 | 1 | 1 | 2 | 13.50 | 8.00 |
| 2023 | CML182 | 1 | 1 | 2 | 1.00 | 8.80 |
| 2023 | CML182 | 1 | 1 | 1 | 10.20 | 4.50 |
| 2023 | CML182 | 1 | 1 | 2 | 12.60 | 16.50 |
| 2023 | CML182 | 1 | 1 | 2 | 7.40 | 11.20 |
| 2023 | CML182 | 1 | 1 | 1 | 11.30 | 14.00 |
| 2023 | CML182 | 1 | 1 | 2 | 5.10 | 12.40 |
| 2023 | CML182 | 1 | 1 | 2 | 9.00 | 14.00 |
| 2023 | CML182 | 1 | 1 | 2 | 6.50 | 2.00 |

| | | | | | | |
|---|---|---|---|---|---|---|
| 2023 | CML182 | 2 | 5 | 5 | 0.00 | 0.00 |
| 2023 | CML182 | 2 | 1 | 1 | 8.20 | 10.00 |
| 2023 | CML182 | 2 | 1 | 1 | 8.50 | 8.10 |
| 2023 | CML182 | 2 | 1 | 1 | 11.60 | 4.00 |
| 2023 | CML182 | 2 | 1 | 1 | 9.50 | 5.80 |
| 2023 | CML182 | 2 | 1 | 1 | 11.20 | 4.80 |
| 2023 | CML182 | 2 | 1 | 2 | 11.70 | 13.00 |
| 2023 | CML182 | 2 | 1 | 2 | 8.00 | 14.60 |
| 2023 | CML182 | 2 | 1 | 2 | 9.30 | 4.50 |
| 2023 | CML182 | 2 | 1 | 2 | 8.50 | 11.50 |
| 2023 | F252 | 1 | 1 | 1 | 3.60 | 1.30 |
| 2023 | F252 | 1 | 1 | 1 | 1.50 | 3.60 |
| 2023 | F252 | 1 | 1 | 1 | 1.90 | 2.80 |
| 2023 | F252 | 1 | 1 | 1 | 1.00 | 1.60 |
| 2023 | F252 | 1 | 1 | 1 | 5.50 | 3.40 |
| 2023 | F252 | 1 | 1 | 1 | 4.20 | 3.20 |
| 2023 | F252 | 1 | 1 | 2 | 1.50 | 0.50 |
| 2023 | F252 | 1 | 1 | 2 | 1.20 | 2.80 |
| 2023 | F252 | 1 | 1 | 2 | 0.50 | 10.70 |
| 2023 | F252 | 1 | 1 | 2 | 0.50 | 3.20 |
| 2023 | F252 | 2 | 1 | 5 | 1.20 | 0.00 |
| 2023 | F252 | 2 | 1 | 5 | 2.10 | 0.00 |
| 2023 | F252 | 2 | 1 | 2 | 7.40 | 0.50 |
| 2023 | F252 | 2 | 1 | 2 | 9.60 | 0.50 |
| 2023 | F252 | 2 | 1 | 2 | 8.70 | 0.50 |
| 2023 | F252 | 2 | 1 | 2 | 12.90 | 0.50 |
| 2023 | F252 | 2 | 1 | 2 | 11.70 | 1.20 |
| 2023 | F252 | 2 | 1 | 2 | 13.50 | 1.40 |
| 2023 | F252 | 2 | 1 | 2 | 0.50 | 3.50 |
| 2023 | F252 | 2 | 1 | 2 | 2.90 | 3.60 |
| 2023 | B73 | 1 | 1 | 5 | 4.80 | 0.00 |
| 2023 | B73 | 1 | 1 | 5 | 7.50 | 0.00 |
| 2023 | B73 | 1 | 1 | 2 | 1.90 | 0.60 |
| 2023 | B73 | 1 | 1 | 2 | 14.20 | 1.90 |
| 2023 | B73 | 1 | 1 | 2 | 13.50 | 1.10 |
| 2023 | B73 | 1 | 1 | 2 | 4.60 | 7.50 |
| 2023 | B73 | 1 | 1 | 2 | 1.40 | 6.50 |
| 2023 | B73 | 1 | 1 | 2 | 3.20 | 6.40 |
| 2023 | B73 | 1 | 1 | 2 | 7.30 | 1.40 |
| 2023 | B73 | 1 | 1 | 2 | 10.60 | 2.90 |
| 2023 | B73 | 2 | 1 | 5 | 17.00 | 0.00 |
| 2023 | B73 | 2 | 1 | 5 | 16.80 | 0.00 |
| 2023 | B73 | 2 | 1 | 5 | 18.90 | 0.00 |
| 2023 | B73 | 2 | 1 | 5 | 12.10 | 0.00 |
| 2023 | B73 | 2 | 1 | 5 | 23.10 | 0.00 |
| 2023 | B73 | 2 | 1 | 5 | 19.20 | 0.00 |
| 2023 | B73 | 2 | 1 | 2 | 10.50 | 2.90 |
| 2023 | B73 | 2 | 1 | 2 | 14.00 | 3.90 |
| 2023 | B73 | 2 | 1 | 2 | 15.60 | 1.50 |
| 2023 | B73 | 2 | 1 | 2 | 18.40 | 1.90 |

**Table S2a.** Phenotypic measures for the 10 public inbred lines. The length of coleoptile was determined in the inoculated (A) and control (C) kernels of inbred lines in two replications. Values represent the mean (cm) of each line with standard deviation.

| Line | Treatment | Replication | Length of coleoptile |
|---|---|---|---|
| B73 | A | 1 | 2.8 ± 1.28 |
| B73 | A | 2 | 1.0 ± 1.28 |
| B73 | C | 1 | 6.9 ± 6.83 |
| B73 | C | 2 | 16.6 ± 6.83 |
| CE-777 | A | 1 | 10.6 ± 1.20 |
| CE-777 | A | 2 | 8.9 ± 1.20 |
| CE-777 | C | 1 | 6.6 ± 0.21 |
| CE-777 | C | 2 | 6.9 ± 0.21 |
| CML182 | A | 1 | 9.1 ± 1.07 |
| CML182 | A | 2 | 7.6 ± 1.07 |
| CML182 | C | 1 | 7.7 ± 0.66 |
| CML182 | C | 2 | 8.6 ± 0.66 |
| F252 | A | 1 | 3.3 ± 1.51 |
| F252 | A | 2 | 1.2 ± 1.51 |
| F252 | C | 1 | 2.2 ± 3.47 |
| F252 | C | 2 | 7.1 ± 3.47 |
| Hi53 | A | 1 | 5.8 ± 0.10 |
| Hi53 | A | 2 | 5.6 ± 0.10 |
| Hi53 | C | 1 | 9.8 ± 0.81 |
| Hi53 | C | 2 | 11.0 ± 0.81 |
| INBRED 39-1546 | A | 1 | 8.0 ± 0.62 |
| INBRED 39-1546 | A | 2 | 8.9 ± 0.62 |
| INBRED 39-1546 | C | 1 | 11.0 ± 1.63 |
| INBRED 39-1546 | C | 2 | 13.3 ± 1.63 |
| Mp705 | A | 1 | 17.4 ± 1.58 |
| Mp705 | A | 2 | 19.6 ± 1.58 |
| Mp705 | C | 1 | 16.4 ± 1.22 |
| Mp705 | C | 2 | 18.2 ± 1.22 |
| T143 | A | 1 | 7.4 ± 1.32 |
| T143 | A | 2 | 9.3 ± 1.32 |
| T143 | C | 1 | 9.4 ± 0.02 |
| T143 | C | 2 | 9.3 ± 0.02 |
| TSU-CHIAO-HSI-WU 102 | A | 1 | 10.7 ± 1.61 |
| TSU-CHIAO-HSI-WU 102 | A | 2 | 8.4 ± 1.61 |
| TSU-CHIAO-HSI-WU 102 | C | 1 | 13.5 ± 0.95 |
| TSU-CHIAO-HSI-WU 102 | C | 2 | 14.9 ± 0.95 |
| W22 BRINK | A | 1 | 8.7 ± 3.34 |
| W22 BRINK | A | 2 | 13.4 ± 3.34 |
| W22 BRINK | C | 1 | 13.3 ± 0.03 |
| W22 BRINK | C | 2 | 13.4 ± 0.03 |

**Table S2b.** Statistical output (analysis of variance - ANOVA) of the phenotypic measures (length of coleoptile) having Line, Treatment, Replication and the interaction Line * Treatment as parameters. The normality of the dataset was tested prior to ANOVA using the Shapiro-Wilk tests.

| Parameters | Degree of freedom | Sum Square | Mean Square | F value | Pr (>F) | p-value |
|---|---|---|---|---|---|---|
| Line | 9.0 | 534.0 | 59.3 | 14.6 | <0.0001 | *** |
| Treatment | 1.0 | 58.3 | 58.3 | 14.3 | 0.0 | ** |
| Replication | 1.0 | 12.6 | 12.6 | 3.1 | 0.1 | . |
| Line * Treatment | 9.0 | 118.0 | 13.1 | 3.2 | 0.0 | * |

p-value; no significant: ° p< 1, . p<0.1; significant: * p<0.05, ** p<0.01, *** p< 0.001

**Table S2c.** Statistical output (analysis of variance - ANOVA) of the phenotypic measures (length of coleoptile) considering only the inoculated kernels of inbred lines. Line and Replication were considered as parameters. The normality of the dataset was tested prior to ANOVA using the Shapiro-Wilk tests.

| Parameters | Degree of freedom | Sum Square | Mean Square | F value | Pr (>F) | p-value |
|---|---|---|---|---|---|---|
| Line | 9.0 | 397.6 | 44.2 | 16.0 | <0.0001 | *** |
| Replication | 1.0 | 0.0 | 0.0 | 0.0 | 1.0 | ° |

p-value; no significant: ° p< 1, . p<0.1; significant: * p<0.05, ** p<0.01, *** p< 0.001

**Table S3a.** Phenotypic measures for B73 inbred line with raw and transformed data (Log transformation) to achieve the normal distribution. The length of coleoptile was determined in the control (C) and inoculated (A) kernels of this line in two replications.

| Line | Treatment | Replication | Length of coleoptile | Log transformation |
|---|---|---|---|---|
| B73 | C | 1 | 4.80 | 0.68 |
| B73 | C | 1 | 7.50 | 0.87 |
| B73 | C | 1 | 1.90 | 0.28 |
| B73 | C | 1 | 14.20 | 1.15 |
| B73 | C | 1 | 13.50 | 1.13 |
| B73 | C | 1 | 4.60 | 0.66 |
| B73 | C | 1 | 1.40 | 0.15 |
| B73 | C | 1 | 3.20 | 0.50 |
| B73 | C | 1 | 7.30 | 0.86 |
| B73 | C | 1 | 10.60 | 1.02 |
| B73 | C | 2 | 17.00 | 1.23 |
| B73 | C | 2 | 16.80 | 1.22 |
| B73 | C | 2 | 18.90 | 1.28 |
| B73 | C | 2 | 12.10 | 1.08 |
| B73 | C | 2 | 23.10 | 1.36 |
| B73 | C | 2 | 19.20 | 1.28 |
| B73 | C | 2 | 10.50 | 1.02 |
| B73 | C | 2 | 14.00 | 1.15 |
| B73 | C | 2 | 15.60 | 1.19 |
| B73 | C | 2 | 18.40 | 1.26 |
| B73 | A | 1 | 0.00 | 0.00 |
| B73 | A | 1 | 0.00 | 0.00 |
| B73 | A | 1 | 0.60 | -0.222 |
| B73 | A | 1 | 1.90 | 0.28 |
| B73 | A | 1 | 1.10 | 0.04 |
| B73 | A | 1 | 7.50 | 0.87 |
| B73 | A | 1 | 6.50 | 0.81 |
| B73 | A | 1 | 6.40 | 0.81 |
| B73 | A | 1 | 1.40 | 0.15 |
| B73 | A | 1 | 2.90 | 0.46 |
| B73 | A | 2 | 0.00 | 0.00 |
| B73 | A | 2 | 0.00 | 0.00 |
| B73 | A | 2 | 0.00 | 0.00 |
| B73 | A | 2 | 0.00 | 0.00 |
| B73 | A | 2 | 0.00 | 0.00 |
| B73 | A | 2 | 0.00 | 0.00 |
| B73 | A | 2 | 2.90 | 0.46 |
| B73 | A | 2 | 3.90 | 0.59 |
| B73 | A | 2 | 1.50 | 0.18 |
| B73 | A | 2 | 1.90 | 0.28 |

**Table S3b.** Statistical output (analysis of variance - ANOVA) of the phenotypic measures (length of coleoptile) for each inbred line having Treatment and Replication as parameters. The normality of the dataset was tested prior to ANOVA using the Shapiro-Wilk or Kolmogorov-Smirnov tests.

| Line | Normality | Treatment | | | | | |
|---|---|---|---|---|---|---|---|
| | | DF | Sum of squares | Mean squares | F | Pr>F | p-value |
| B73 | Kolmogorov-Smirnov | 1.00 | 5.40 | 5.40 | 64.38 | <0.0001 | *** |
| F252 | Kolmogorov-Smirnov | 1.00 | 55.46 | 55.46 | 5.91 | 0.020 | * |
| T143 | Kolmogorov-Smirnov | 1.00 | 9.70 | 9.70 | 0.31 | 0.584 | ° |
| CML182 | Shapiro-Wilk | 1.00 | 0.42 | 0.42 | 0.02 | 0.888 | ° |
| W22 BRINK | Shapiro-Wilk | 1.00 | 52.44 | 52.44 | 2.31 | 0.137 | ° |
| Hi53 | Shapiro-Wilk | 1.00 | 222.78 | 222.78 | 13.15 | 0.001 | *** |
| TSU-CHIAO-HSI-WU 102 | Shapiro-Wilk | 1.00 | 213.91 | 213.91 | 28.14 | <0.0001 | *** |
| INBRED 39-1546 | Shapiro-Wilk | 1.00 | 141.75 | 141.75 | 5.88 | 0.020 | * |
| CE-777 | Shapiro-Wilk | 1.00 | 91.20 | 91.20 | 5.94 | 0.020 | * |
| Mp705 (*) | Kolmogorov-Smirnov | 1.00 | 15.35 | 15.35 | 0.99 | 0.325 | ° |

p-value; no significant: ° p< 1, . p<0.1; significant: * p<0.05, ** p<0.01, *** p< 0.001

(*) two observed outliers of the dataset for this line have been replaced by the average value of replication.

**Table S4a.** Phenotypic measures of 14 maize hybrids labeled with specific code. The length of coleoptile was determined in the inoculated (A) and control (C) kernels of hybrids in two replications. Values represent the mean (cm) of each hybrid.

| Hybrid Code | Treatment | Replication | Length of coleoptile |
|---|---|---|---|
| 1a | A | 1 | 11.20 |
| 1a | A | 2 | 15.10 |
| 1a | C | 1 | 8.39 |
| 1a | C | 2 | 12.30 |
| 1b | A | 1 | 15.30 |
| 1b | A | 2 | 13.50 |
| 1b | C | 1 | 13.20 |
| 1b | C | 2 | 13.40 |
| 2a | A | 1 | 10.70 |
| 2a | A | 2 | 6.42 |
| 2a | C | 1 | 11.30 |
| 2a | C | 2 | 13.50 |
| 2b | A | 1 | 7.51 |
| 2b | A | 2 | 5.59 |
| 2b | C | 1 | 6.40 |
| 2b | C | 2 | 8.32 |
| 3a | A | 1 | 15.10 |
| 3a | A | 2 | 14.00 |
| 3a | C | 1 | 14.00 |
| 3a | C | 2 | 14.30 |
| 3b | A | 1 | 10.20 |
| 3b | A | 2 | 11.40 |
| 3b | C | 1 | 10.60 |
| 3b | C | 2 | 12.10 |
| 4a | A | 1 | 3.92 |
| 4a | A | 2 | 2.61 |
| 4a | C | 1 | 18.60 |
| 4a | C | 2 | 14.70 |
| 4b | A | 1 | 2.53 |
| 4b | A | 2 | 7.73 |
| 4b | C | 1 | 10.80 |
| 4b | C | 2 | 8.10 |
| 5a | A | 1 | 4.04 |
| 5a | A | 2 | 6.35 |
| 5a | C | 1 | 16.40 |
| 5a | C | 2 | 13.00 |
| 5b | A | 1 | 2.02 |
| 5b | A | 2 | 3.82 |
| 5b | C | 1 | 17.00 |
| 5b | C | 2 | 8.23 |
| 6a | A | 1 | 16.00 |
| 6a | A | 2 | 21.30 |
| 6a | C | 1 | 20.20 |
| 6a | C | 2 | 20.00 |

| | | | |
|---|---|---|---|
| 6b | A | 1 | 7.31 |
| 6b | A | 2 | 16.80 |
| 6b | C | 1 | 21.30 |
| 6b | C | 2 | 23.30 |
| 7a | A | 1 | 9.92 |
| 7a | A | 2 | 17.10 |
| 7a | C | 1 | 21.10 |
| 7a | C | 2 | 22.90 |
| 7b | A | 1 | 9.68 |
| 7b | A | 2 | 13.90 |
| 7b | C | 1 | 17.80 |
| 7b | C | 2 | 21.50 |

**Table S4b.** Statistical output (analysis of variance - ANOVA) of the phenotypic measures (length of coleoptile) having Hybrid Code, Treatment and Replication as parameters. The normality of the dataset was tested prior to ANOVA using the Shapiro-Wilk tests.

| Parameters | Degree of freedom | Sum Square | Mean Square | F value | Pr (>F) | p-value |
|---|---|---|---|---|---|---|
| Hybrid Code | 13.0 | 850.64 | 65.4 | 4.9 | <0.0001 | *** |
| Treatment | 1.0 | 308.8 | 308.8 | 23.0 | <0.0001 | *** |
| Replication | 1.0 | 14.8 | 14.8 | 1.1 | 0.299 | ° |

p-value; no significant: ° p< 1, . p<0.1; significant: * p<0.05, ** p<0.01, *** p< 0.001

**Table S4c.** Statistical output (analysis of variance - ANOVA) of the phenotypic measures (length of coleoptile) considering only the inoculated 7 maize hybrids. Hybrid Code and Replication used as parameters. The normality of the dataset was tested prior to ANOVA using the Shapiro-Wilk tests.

| Parameters | Degree of freedom | Sum Square | Mean Square | F value | Pr (>F) | p-value |
|---|---|---|---|---|---|---|
| Hybrid Code | 6.0 | 350.60 | 58.4 | 9.2 | 0.0 | ** |
| Replication | 1.0 | 6.1 | 6.1 | 1.0 | 0.4 | ° |

p-value; no significant: ° p< 1, . p<0.1; significant: * p<0.05, ** p<0.01, *** p< 0.001

**Table S5a.** Phenotypic measures of the maize hybrid 6a and its reciprocal 6b. The length of coleoptile was determined in the inoculated (A) kernels in two replications.

| Hybrid Code | Treatment | Replication | Length of coleoptile |
|---|---|---|---|
| 6a | A | 1 | 21.50 |
| 6a | A | 1 | 21.70 |
| 6a | A | 1 | 14.50 |
| 6a | A | 1 | 12.10 |
| 6a | A | 1 | 8.60 |
| 6a | A | 1 | 12.50 |
| 6a | A | 1 | 17.50 |
| 6a | A | 1 | 16.20 |
| 6a | A | 1 | 19.40 |
| 6a | A | 1 | 15.70 |
| 6a | A | 2 | 20.70 |
| 6a | A | 2 | 19.6 |
| 6a | A | 2 | 21.30 |
| 6a | A | 2 | 25.50 |
| 6a | A | 2 | 20.50 |
| 6a | A | 2 | 20.70 |
| 6a | A | 2 | 18.50 |
| 6a | A | 2 | 20.80 |
| 6a | A | 2 | 22.00 |
| 6a | A | 2 | 23.60 |
| 6b | A | 1 | 0.00 |
| 6b | A | 1 | 3.50 |
| 6b | A | 1 | 9.50 |
| 6b | A | 1 | 10.70 |
| 6b | A | 1 | 10.50 |
| 6b | A | 1 | 9.20 |
| 6b | A | 1 | 8.70 |
| 6b | A | 1 | 7.50 |
| 6b | A | 1 | 5.20 |
| 6b | A | 1 | 8.30 |
| 6b | A | 2 | 10.00 |
| 6b | A | 2 | 4.50 |
| 6b | A | 2 | 24.20 |
| 6b | A | 2 | 21.70 |
| 6b | A | 2 | 21.50 |
| 6b | A | 2 | 15.30 |
| 6b | A | 2 | 19.00 |
| 6b | A | 2 | 20.70 |
| 6b | A | 2 | 21.00 |
| 6b | A | 2 | 10.00 |

**Table S5b.** Statistical output (analysis of variance - ANOVA) of the phenotypic measures (length of coleoptile) having Hybrid Code as parameter. The normality of the dataset was tested prior to ANOVA using Kolmogorov-Smirnov test.

| Parameters | Degree of freedom | Sum Square | Mean Square | F value | Pr (>F) | p-value |
|---|---|---|---|---|---|---|
| Hybrid Code | 1.0 | 434.90 | 434.90 | 22.1 | <0.0001 | *** |

p-value; no significant: ° p< 1, . p<0.1; significant: * p<0.05, ** p<0.01, *** p< 0.001

**Table S6a.** Phenotypic measures for 16 inbred lines/year tested. The inbred lines have a specific code; the length of coleoptile (cm) was determined in the control (C) and inoculated (A) kernels in two replications.

| Year | Line Code | Replication | Length of coleoptile (C) | Length of coleoptile (A) |
|---|---|---|---|---|
| 2001 | 1 | 1 | 3.10 | 0.00 |
| 2001 | 1 | 1 | 3.70 | 0.00 |
| 2001 | 1 | 1 | 3.90 | 0.00 |
| 2001 | 1 | 1 | 1.50 | 0.00 |
| 2001 | 1 | 1 | 7.50 | 0.00 |
| 2001 | 1 | 1 | 3.60 | 0.00 |
| 2001 | 1 | 1 | 0.50 | 0.00 |
| 2001 | 1 | 1 | 0.00 | 0.00 |
| 2001 | 1 | 1 | 0.00 | 4.70 |
| 2001 | 1 | 1 | 0.00 | 4.20 |
| 2001 | 1 | 2 | 0.00 | 0.00 |
| 2001 | 1 | 2 | 0.00 | 0.00 |
| 2001 | 1 | 2 | 0.00 | 0.00 |
| 2001 | 1 | 2 | 0.00 | 0.00 |
| 2001 | 1 | 2 | 0.00 | 5.50 |
| 2001 | 1 | 2 | 5.50 | 4.70 |
| 2001 | 1 | 2 | 6.00 | 3.00 |
| 2001 | 1 | 2 | 3.30 | 2.60 |
| 2001 | 1 | 2 | 2.00 | 2.10 |
| 2001 | 1 | 2 | 1.50 | 3.60 |
| 2001 | 2 | 1 | 0.00 | 0.00 |
| 2001 | 2 | 1 | 3.30 | 2.60 |
| 2001 | 2 | 1 | 5.10 | 6.90 |
| 2001 | 2 | 1 | 6.40 | 7.20 |
| 2001 | 2 | 1 | 0.50 | 0.50 |
| 2001 | 2 | 1 | 2.20 | 0.50 |
| 2001 | 2 | 1 | 1.90 | 6.20 |
| 2001 | 2 | 1 | 2.10 | 3.10 |
| 2001 | 2 | 1 | 2.60 | 5.30 |
| 2001 | 2 | 1 | 0.50 | 3.60 |
| 2001 | 2 | 2 | 0.00 | 0.00 |

| | | | | |
|---|---|---|---|---|
| 2001 | 2 | 2 | 0.00 | 0.00 |
| 2001 | 2 | 2 | 0.00 | 7.30 |
| 2001 | 2 | 2 | 0.00 | 9.60 |
| 2001 | 2 | 2 | 0.00 | 0.90 |
| 2001 | 2 | 2 | 0.00 | 2.60 |
| 2001 | 2 | 2 | 5.50 | 1.50 |
| 2001 | 2 | 2 | 2.50 | 6.80 |
| 2001 | 2 | 2 | 0.50 | 5.40 |
| 2001 | 2 | 2 | 0.50 | 3.20 |
| 2001 | 4 | 1 | 0.00 | 0.00 |
| 2001 | 4 | 1 | 0.00 | 0.00 |
| 2001 | 4 | 1 | 0.00 | 0.00 |
| 2001 | 4 | 1 | 0.00 | 0.00 |
| 2001 | 4 | 1 | 0.00 | 0.00 |
| 2001 | 4 | 1 | 2.90 | 0.00 |
| 2001 | 4 | 1 | 4.70 | 4.00 |
| 2001 | 4 | 1 | 4.60 | 4.00 |
| 2001 | 4 | 1 | 8.40 | 4.20 |
| 2001 | 4 | 1 | 4.90 | 4.50 |
| 2001 | 4 | 2 | 0.00 | 0.00 |
| 2001 | 4 | 2 | 0.00 | 0.00 |
| 2001 | 4 | 2 | 0.00 | 0.00 |
| 2001 | 4 | 2 | 0.00 | 0.00 |
| 2001 | 4 | 2 | 0.00 | 0.00 |
| 2001 | 4 | 2 | 4.20 | 5.30 |
| 2001 | 4 | 2 | 5.70 | 6.50 |
| 2001 | 4 | 2 | 6.50 | 2.60 |
| 2001 | 4 | 2 | 14.20 | 4.90 |
| 2001 | 4 | 2 | 4.60 | 2.80 |
| 2001 | 5 | 1 | 1.90 | 2.80 |
| 2001 | 5 | 1 | 4.60 | 4.70 |
| 2001 | 5 | 1 | 5.00 | 7.00 |
| 2001 | 5 | 1 | 6.40 | 4.90 |
| 2001 | 5 | 1 | 7.80 | 4.60 |
| 2001 | 5 | 1 | 6.90 | 4.80 |
| 2001 | 5 | 1 | 4.90 | 5.00 |
| 2001 | 5 | 1 | 4.60 | 0.50 |
| 2001 | 5 | 1 | 4.70 | 3.10 |
| 2001 | 5 | 1 | 4.50 | 2.10 |
| 2001 | 5 | 2 | 0.00 | 0.00 |
| 2001 | 5 | 2 | 0.00 | 9.80 |
| 2001 | 5 | 2 | 3.60 | 7.40 |
| 2001 | 5 | 2 | 0.70 | 3.50 |
| 2001 | 5 | 2 | 5.10 | 6.40 |
| 2001 | 5 | 2 | 9.30 | 5.90 |
| 2001 | 5 | 2 | 7.90 | 7.90 |
| 2001 | 5 | 2 | 10.60 | 4.50 |

| | | | | |
|---|---|---|---|---|
| 2001 | 5 | 2 | 7.60 | 3.70 |
| 2001 | 5 | 2 | 6.80 | 3.60 |
| 2001 | 6 | 1 | 14.30 | 0.00 |
| 2001 | 6 | 1 | 5.00 | 0.00 |
| 2001 | 6 | 1 | 8.50 | 0.00 |
| 2001 | 6 | 1 | 8.30 | 0.00 |
| 2001 | 6 | 1 | 8.00 | 0.00 |
| 2001 | 6 | 1 | 3.00 | 0.00 |
| 2001 | 6 | 1 | 4.60 | 1.00 |
| 2001 | 6 | 1 | 7.60 | 3.70 |
| 2001 | 6 | 1 | 14.40 | 6.60 |
| 2001 | 6 | 1 | 12.30 | 9.50 |
| 2001 | 6 | 2 | 2.50 | 0.00 |
| 2001 | 6 | 2 | 3.00 | 0.00 |
| 2001 | 6 | 2 | 4.40 | 0.00 |
| 2001 | 6 | 2 | 5.00 | 0.00 |
| 2001 | 6 | 2 | 11.50 | 0.00 |
| 2001 | 6 | 2 | 18.00 | 0.00 |
| 2001 | 6 | 2 | 12.00 | 0.00 |
| 2001 | 6 | 2 | 5.40 | 0.00 |
| 2001 | 6 | 2 | 0.00 | 7.00 |
| 2001 | 6 | 2 | 0.00 | 10.00 |
| 2001 | 7 | 1 | 0.00 | 6.20 |
| 2001 | 7 | 1 | 0.00 | 6.00 |
| 2001 | 7 | 1 | 1.80 | 4.80 |
| 2001 | 7 | 1 | 0.90 | 4.30 |
| 2001 | 7 | 1 | 3.20 | 4.40 |
| 2001 | 7 | 1 | 5.20 | 0.00 |
| 2001 | 7 | 1 | 3.10 | 0.00 |
| 2001 | 7 | 1 | 3.20 | 0.00 |
| 2001 | 7 | 1 | 1.80 | 0.00 |
| 2001 | 7 | 1 | 3.90 | 0.00 |
| 2001 | 7 | 2 | 0.00 | 0.00 |
| 2001 | 7 | 2 | 0.00 | 0.00 |
| 2001 | 7 | 2 | 0.00 | 0.00 |
| 2001 | 7 | 2 | 0.00 | 0.00 |
| 2001 | 7 | 2 | 0.00 | 0.00 |
| 2001 | 7 | 2 | 3.00 | 0.00 |
| 2001 | 7 | 2 | 2.30 | 3.50 |
| 2001 | 7 | 2 | 2.50 | 2.00 |
| 2001 | 7 | 2 | 0.10 | 5.00 |
| 2001 | 7 | 2 | 0.10 | 5.00 |
| 2001 | 8 | 1 | 0.00 | 0.00 |
| 2001 | 8 | 1 | 0.00 | 0.00 |
| 2001 | 8 | 1 | 9.30 | 0.00 |
| 2001 | 8 | 1 | 5.60 | 0.00 |
| 2001 | 8 | 1 | 3.90 | 0.00 |

| | | | | |
|---|---|---|---|---|
| 2001 | 8 | 1 | 3.50 | 2.90 |
| 2001 | 8 | 1 | 2.50 | 2.50 |
| 2001 | 8 | 1 | 5.50 | 3.80 |
| 2001 | 8 | 1 | 9.30 | 0.50 |
| 2001 | 8 | 1 | 7.50 | 0.50 |
| 2001 | 8 | 2 | 0.00 | 0.00 |
| 2001 | 8 | 2 | 0.00 | 0.00 |
| 2001 | 8 | 2 | 0.00 | 3.70 |
| 2001 | 8 | 2 | 2.90 | 0.50 |
| 2001 | 8 | 2 | 3.90 | 6.70 |
| 2001 | 8 | 2 | 3.00 | 3.30 |
| 2001 | 8 | 2 | 2.50 | 6.60 |
| 2001 | 8 | 2 | 3.80 | 5.40 |
| 2001 | 8 | 2 | 3.60 | 0.00 |
| 2001 | 8 | 2 | 3.10 | 0.00 |
| 2001 | 9 | 1 | 0.00 | 0.00 |
| 2001 | 9 | 1 | 0.00 | 0.00 |
| 2001 | 9 | 1 | 10.90 | 0.00 |
| 2001 | 9 | 1 | 7.40 | 0.00 |
| 2001 | 9 | 1 | 10.90 | 0.00 |
| 2001 | 9 | 1 | 6.70 | 0.00 |
| 2001 | 9 | 1 | 3.50 | 0.00 |
| 2001 | 9 | 1 | 1.50 | 5.00 |
| 2001 | 9 | 1 | 9.50 | 8.50 |
| 2001 | 9 | 1 | 5.40 | 0.00 |
| 2001 | 9 | 2 | 0.00 | 0.00 |
| 2001 | 9 | 2 | 5.60 | 0.00 |
| 2001 | 9 | 2 | 8.50 | 11.20 |
| 2001 | 9 | 2 | 14.00 | 14.40 |
| 2001 | 9 | 2 | 2.50 | 8.90 |
| 2001 | 9 | 2 | 5.50 | 2.00 |
| 2001 | 9 | 2 | 5.00 | 4.20 |
| 2001 | 9 | 2 | 3.00 | 3.00 |
| 2001 | 9 | 2 | 7.50 | 3.70 |
| 2001 | 9 | 2 | 4.00 | 4.50 |
| 2001 | 10 | 1 | 0.00 | 0.00 |
| 2001 | 10 | 1 | 0.00 | 0.00 |
| 2001 | 10 | 1 | 6.20 | 0.00 |
| 2001 | 10 | 1 | 4.90 | 0.00 |
| 2001 | 10 | 1 | 7.30 | 0.00 |
| 2001 | 10 | 1 | 7.20 | 0.00 |
| 2001 | 10 | 1 | 1.60 | 2.60 |
| 2001 | 10 | 1 | 2.30 | 5.50 |
| 2001 | 10 | 1 | 3.70 | 4.00 |
| 2001 | 10 | 1 | 2.90 | 3.60 |
| 2001 | 10 | 2 | 0.00 | 0.00 |
| 2001 | 10 | 2 | 4.20 | 0.00 |

| | | | | |
|---|---|---|---|---|
| 2001 | 10 | 2 | 5.60 | 0.00 |
| 2001 | 10 | 2 | 4.30 | 2.00 |
| 2001 | 10 | 2 | 2.60 | 2.80 |
| 2001 | 10 | 2 | 10.80 | 2.70 |
| 2001 | 10 | 2 | 0.50 | 1.80 |
| 2001 | 10 | 2 | 2.30 | 1.80 |
| 2001 | 10 | 2 | 10.50 | 1.50 |
| 2001 | 10 | 2 | 3.70 | 3.00 |
| 2001 | 11 | 1 | 0.00 | 0.00 |
| 2001 | 11 | 1 | 3.90 | 11.00 |
| 2001 | 11 | 1 | 4.40 | 3.00 |
| 2001 | 11 | 1 | 5.70 | 10.00 |
| 2001 | 11 | 1 | 5.80 | 5.40 |
| 2001 | 11 | 1 | 7.90 | 7.30 |
| 2001 | 11 | 1 | 6.50 | 3.00 |
| 2001 | 11 | 1 | 7.60 | 10.00 |
| 2001 | 11 | 1 | 3.70 | 7.80 |
| 2001 | 11 | 1 | 12.90 | 10.30 |
| 2001 | 11 | 2 | 0.00 | 0.00 |
| 2001 | 11 | 2 | 5.20 | 0.00 |
| 2001 | 11 | 2 | 11.10 | 0.00 |
| 2001 | 11 | 2 | 14.50 | 0.00 |
| 2001 | 11 | 2 | 13.80 | 4.80 |
| 2001 | 11 | 2 | 7.60 | 4.00 |
| 2001 | 11 | 2 | 14.30 | 7.80 |
| 2001 | 11 | 2 | 5.70 | 9.80 |
| 2001 | 11 | 2 | 4.20 | 6.40 |
| 2001 | 11 | 2 | 6.80 | 6.50 |
| 2001 | 12 | 1 | 0.00 | 0.00 |
| 2001 | 12 | 1 | 0.00 | 0.00 |
| 2001 | 12 | 1 | 9.80 | 0.00 |
| 2001 | 12 | 1 | 10.60 | 0.00 |
| 2001 | 12 | 1 | 2.70 | 5.00 |
| 2001 | 12 | 1 | 13.40 | 8.30 |
| 2001 | 12 | 1 | 8.20 | 7.00 |
| 2001 | 12 | 1 | 13.00 | 11.00 |
| 2001 | 12 | 1 | 4.80 | 4.90 |
| 2001 | 12 | 1 | 5.90 | 7.80 |
| 2001 | 12 | 2 | 7.40 | 0.00 |
| 2001 | 12 | 2 | 4.20 | 0.00 |
| 2001 | 12 | 2 | 6.30 | 2.40 |
| 2001 | 12 | 2 | 3.90 | 1.50 |
| 2001 | 12 | 2 | 2.60 | 1.50 |
| 2001 | 12 | 2 | 4.20 | 5.50 |
| 2001 | 12 | 2 | 1.20 | 1.50 |
| 2001 | 12 | 2 | 2.70 | 2.50 |
| 2001 | 12 | 2 | 4.40 | 2.50 |

| | | | | |
|---|---|---|---|---|
| 2001 | 12 | 2 | 1.60 | 4.00 |
| 2001 | 13 | 1 | 0.00 | 0.00 |
| 2001 | 13 | 1 | 13.20 | 0.00 |
| 2001 | 13 | 1 | 10.50 | 0.00 |
| 2001 | 13 | 1 | 7.50 | 13.50 |
| 2001 | 13 | 1 | 3.60 | 11.70 |
| 2001 | 13 | 1 | 9.30 | 8.20 |
| 2001 | 13 | 1 | 10.20 | 10.30 |
| 2001 | 13 | 1 | 15.40 | 8.40 |
| 2001 | 13 | 1 | 14.70 | 8.50 |
| 2001 | 13 | 1 | 0.00 | 11.20 |
| 2001 | 13 | 2 | 0.00 | 0.00 |
| 2001 | 13 | 2 | 6.80 | 4.00 |
| 2001 | 13 | 2 | 11.70 | 4.40 |
| 2001 | 13 | 2 | 3.90 | 5.00 |
| 2001 | 13 | 2 | 2.70 | 6.00 |
| 2001 | 13 | 2 | 12.30 | 6.20 |
| 2001 | 13 | 2 | 4.30 | 3.80 |
| 2001 | 13 | 2 | 5.90 | 4.30 |
| 2001 | 13 | 2 | 6.70 | 3.60 |
| 2001 | 13 | 2 | 1.80 | 3.50 |
| 2001 | 14 | 1 | 0.00 | 0.00 |
| 2001 | 14 | 1 | 0.00 | 3.20 |
| 2001 | 14 | 1 | 0.00 | 4.10 |
| 2001 | 14 | 1 | 3.20 | 32.00 |
| 2001 | 14 | 1 | 1.20 | 2.30 |
| 2001 | 14 | 1 | 1.10 | 2.60 |
| 2001 | 14 | 1 | 1.30 | 2.80 |
| 2001 | 14 | 1 | 1.50 | 2.80 |
| 2001 | 14 | 1 | 0.70 | 3.00 |
| 2001 | 14 | 1 | 2.50 | 2.50 |
| 2001 | 14 | 2 | 0.00 | 0.00 |
| 2001 | 14 | 2 | 0.00 | 2.00 |
| 2001 | 14 | 2 | 2.50 | 3.00 |
| 2001 | 14 | 2 | 3.00 | 2.40 |
| 2001 | 14 | 2 | 2.50 | 2.00 |
| 2001 | 14 | 2 | 2.70 | 1.50 |
| 2001 | 14 | 2 | 3.20 | 2.50 |
| 2001 | 14 | 2 | 3.30 | 2.40 |
| 2001 | 14 | 2 | 3.20 | 2.00 |
| 2001 | 14 | 2 | 0.50 | 2.50 |
| 2001 | 15 | 1 | 0.00 | 4.30 |
| 2001 | 15 | 1 | 0.00 | 11.70 |
| 2001 | 15 | 1 | 0.00 | 3.70 |
| 2001 | 15 | 1 | 5.40 | 5.30 |
| 2001 | 15 | 1 | 6.30 | 3.80 |
| 2001 | 15 | 1 | 7.50 | 0.00 |

| | | | | |
|---|---|---|---|---|
| 2001 | 15 | 1 | 7.80 | 0.00 |
| 2001 | 15 | 1 | 4.20 | 0.00 |
| 2001 | 15 | 1 | 1.50 | 0.00 |
| 2001 | 15 | 1 | 0.00 | 0.00 |
| 2001 | 15 | 2 | 0.00 | 0.00 |
| 2001 | 15 | 2 | 0.00 | 0.00 |
| 2001 | 15 | 2 | 6.20 | 0.00 |
| 2001 | 15 | 2 | 4.30 | 2.00 |
| 2001 | 15 | 2 | 9.00 | 3.80 |
| 2001 | 15 | 2 | 6.50 | 2.50 |
| 2001 | 15 | 2 | 5.20 | 4.50 |
| 2001 | 15 | 2 | 2.50 | 0.00 |
| 2001 | 15 | 2 | 3.60 | 0.00 |
| 2001 | 15 | 2 | 0.80 | 0.00 |
| 2001 | 16 | 1 | 0.00 | 0.00 |
| 2001 | 16 | 1 | 0.00 | 0.00 |
| 2001 | 16 | 1 | 2.10 | 0.00 |
| 2001 | 16 | 1 | 0.70 | 0.00 |
| 2001 | 16 | 1 | 2.60 | 0.00 |
| 2001 | 16 | 1 | 2.00 | 4.40 |
| 2001 | 16 | 1 | 0.40 | 4.60 |
| 2001 | 16 | 1 | 0.30 | 1.00 |
| 2001 | 16 | 1 | 2.60 | 1.50 |
| 2001 | 16 | 1 | 1.80 | 0.00 |
| 2001 | 16 | 2 | 0.00 | 0.00 |
| 2001 | 16 | 2 | 0.00 | 0.00 |
| 2001 | 16 | 2 | 0.00 | 0.00 |
| 2001 | 16 | 2 | 0.00 | 0.00 |
| 2001 | 16 | 2 | 0.00 | 0.00 |
| 2001 | 16 | 2 | 2.60 | 0.00 |
| 2001 | 16 | 2 | 1.00 | 0.00 |
| 2001 | 16 | 2 | 2.80 | 0.00 |
| 2001 | 16 | 2 | 1.50 | 0.00 |
| 2001 | 16 | 2 | 0.50 | 1.80 |
| 2010 | 20 | 1 | 7.50 | 1.90 |
| 2010 | 20 | 1 | 6.60 | 4.10 |
| 2010 | 20 | 1 | 11.40 | 6.80 |
| 2010 | 20 | 1 | 9.20 | 5.90 |
| 2010 | 20 | 1 | 3.60 | 9.40 |
| 2010 | 20 | 1 | 5.30 | 9.80 |
| 2010 | 20 | 1 | 4.20 | 1.80 |
| 2010 | 20 | 1 | 7.30 | 7.40 |
| 2010 | 20 | 1 | 2.50 | 6.80 |
| 2010 | 20 | 2 | 0.50 | 7.30 |
| 2010 | 20 | 2 | 2.80 | 0.50 |
| 2010 | 20 | 2 | 9.00 | 2.40 |
| 2010 | 20 | 2 | 10.50 | 8.40 |

| | | | | |
|---|---|---|---|---|
| 2010 | 20 | 2 | 11.80 | 5.10 |
| 2010 | 20 | 2 | 9.90 | 2.30 |
| 2010 | 20 | 2 | 10.90 | 7.30 |
| 2010 | 20 | 2 | 10.40 | 6.40 |
| 2010 | 20 | 2 | 6.40 | 6.10 |
| 2010 | 20 | 2 | 5.60 | 2.50 |
| 2010 | 20 | 1 | 3.50 | 3.40 |
| 2010 | 21 | 1 | 4.80 | 4.00 |
| 2010 | 21 | 1 | 5.10 | 7.30 |
| 2010 | 21 | 1 | 6.10 | 1.50 |
| 2010 | 21 | 1 | 4.60 | 8.80 |
| 2010 | 21 | 1 | 6.90 | 6.50 |
| 2010 | 21 | 1 | 5.60 | 7.00 |
| 2010 | 21 | 1 | 7.50 | 5.60 |
| 2010 | 21 | 1 | 6.20 | 5.90 |
| 2010 | 21 | 1 | 10.00 | 6.10 |
| 2010 | 21 | 1 | 5.50 | 4.50 |
| 2010 | 21 | 2 | 10.20 | 5.20 |
| 2010 | 21 | 2 | 11.50 | 6.60 |
| 2010 | 21 | 2 | 4.00 | 6.70 |
| 2010 | 21 | 2 | 5.60 | 2.60 |
| 2010 | 21 | 2 | 4.60 | 8.10 |
| 2010 | 21 | 2 | 9.70 | 1.90 |
| 2010 | 21 | 2 | 8.50 | 1.80 |
| 2010 | 21 | 2 | 8.90 | 4.30 |
| 2010 | 21 | 2 | 10.40 | 6.50 |
| 2010 | 21 | 2 | 8.70 | 5.80 |
| 2010 | 22 | 1 | 4.20 | 0.00 |
| 2010 | 22 | 1 | 0.50 | 0.00 |
| 2010 | 22 | 1 | 2.20 | 0.00 |
| 2010 | 22 | 1 | 3.20 | 0.00 |
| 2010 | 22 | 1 | 3.50 | 0.00 |
| 2010 | 22 | 1 | 2.50 | 0.00 |
| 2010 | 22 | 1 | 1.50 | 4.30 |
| 2010 | 22 | 1 | 4.50 | 4.60 |
| 2010 | 22 | 1 | 2.50 | 2.80 |
| 2010 | 22 | 1 | 1.20 | 3.40 |
| 2010 | 22 | 2 | 3.60 | 0.00 |
| 2010 | 22 | 2 | 8.10 | 0.00 |
| 2010 | 22 | 2 | 8.90 | 0.50 |
| 2010 | 22 | 2 | 8.40 | 0.50 |
| 2010 | 22 | 2 | 9.60 | 0.50 |
| 2010 | 22 | 2 | 3.20 | 0.50 |
| 2010 | 22 | 2 | 11.50 | 0.50 |
| 2010 | 22 | 2 | 6.90 | 0.40 |
| 2010 | 22 | 2 | 9.30 | 1.10 |
| 2010 | 22 | 2 | 11.40 | 3.10 |

| | | | | |
|---|---|---|---|---|
| 2010 | 23 | 1 | 0.00 | 6.30 |
| 2010 | 23 | 1 | 0.00 | 5.20 |
| 2010 | 23 | 1 | 0.00 | 6.00 |
| 2010 | 23 | 1 | 3.50 | 7.60 |
| 2010 | 23 | 1 | 2.90 | 3.40 |
| 2010 | 23 | 1 | 5.20 | 4.30 |
| 2010 | 23 | 1 | 4.30 | 0.50 |
| 2010 | 23 | 1 | 0.50 | 3.20 |
| 2010 | 23 | 1 | 5.50 | 5.60 |
| 2010 | 23 | 1 | 2.20 | 7.30 |
| 2010 | 23 | 2 | 3.60 | 9.40 |
| 2010 | 23 | 2 | 1.90 | 3.60 |
| 2010 | 23 | 2 | 5.50 | 7.20 |
| 2010 | 23 | 2 | 5.80 | 6.50 |
| 2010 | 23 | 2 | 8.60 | 4.40 |
| 2010 | 23 | 2 | 4.50 | 5.20 |
| 2010 | 23 | 2 | 4.30 | 2.90 |
| 2010 | 23 | 2 | 4.40 | 9.20 |
| 2010 | 23 | 2 | 4.30 | 4.10 |
| 2010 | 23 | 2 | 4.10 | 6.30 |
| 2010 | 24 | 1 | 0.50 | 0.50 |
| 2010 | 24 | 1 | 1.70 | 6.30 |
| 2010 | 24 | 1 | 1.20 | 4.20 |
| 2010 | 24 | 1 | 1.90 | 5.10 |
| 2010 | 24 | 1 | 1.60 | 4.30 |
| 2010 | 24 | 1 | 1.70 | 3.60 |
| 2010 | 24 | 1 | 6.50 | 4.90 |
| 2010 | 24 | 1 | 5.50 | 4.10 |
| 2010 | 24 | 1 | 1.30 | 2.30 |
| 2010 | 24 | 1 | 0.00 | 4.20 |
| 2010 | 24 | 2 | 0.00 | 7.70 |
| 2010 | 24 | 2 | 1.60 | 5.40 |
| 2010 | 24 | 2 | 2.00 | 5.60 |
| 2010 | 24 | 2 | 3.50 | 4.80 |
| 2010 | 24 | 2 | 0.50 | 4.50 |
| 2010 | 24 | 2 | 0.50 | 0.50 |
| 2010 | 24 | 2 | 2.60 | 0.50 |
| 2010 | 24 | 2 | 5.50 | 3.30 |
| 2010 | 24 | 2 | 5.30 | 4.20 |
| 2010 | 24 | 2 | 0.70 | 5.10 |
| 2010 | 25 | 1 | 3.20 | 10.40 |
| 2010 | 25 | 1 | 12.50 | 13.30 |
| 2010 | 25 | 1 | 13.00 | 11.30 |
| 2010 | 25 | 1 | 11.00 | 9.60 |
| 2010 | 25 | 1 | 11.50 | 12.20 |
| 2010 | 25 | 1 | 10.50 | 14.70 |
| 2010 | 25 | 1 | 9.20 | 11.10 |

| | | | | |
|---|---|---|---|---|
| 2010 | 25 | 1 | 12.50 | 10.50 |
| 2010 | 25 | 1 | 10.70 | 6.60 |
| 2010 | 25 | 1 | 0.00 | 11.60 |
| 2010 | 25 | 2 | 0.50 | 11.20 |
| 2010 | 25 | 2 | 9.20 | 12.20 |
| 2010 | 25 | 2 | 14.30 | 3.50 |
| 2010 | 25 | 2 | 15.80 | 8.50 |
| 2010 | 25 | 2 | 12.00 | 6.30 |
| 2010 | 25 | 2 | 10.20 | 5.50 |
| 2010 | 25 | 2 | 18.50 | 0.90 |
| 2010 | 25 | 2 | 18.90 | 5.90 |
| 2010 | 25 | 2 | 9.60 | 3.80 |
| 2010 | 25 | 2 | 10.80 | 0.00 |
| 2010 | 26 | 1 | 1.00 | 0.50 |
| 2010 | 26 | 1 | 0.50 | 0.50 |
| 2010 | 26 | 1 | 0.50 | 0.50 |
| 2010 | 26 | 1 | 1.20 | 0.50 |
| 2010 | 26 | 1 | 1.40 | 0.50 |
| 2010 | 26 | 1 | 0.00 | 2.30 |
| 2010 | 26 | 1 | 0.00 | 1.90 |
| 2010 | 26 | 1 | 0.00 | 1.60 |
| 2010 | 26 | 1 | 0.00 | 3.20 |
| 2010 | 26 | 1 | 0.00 | 4.10 |
| 2010 | 26 | 2 | 0.50 | 1.20 |
| 2010 | 26 | 2 | 3.20 | 0.00 |
| 2010 | 26 | 2 | 3.90 | 0.50 |
| 2010 | 26 | 2 | 0.50 | 0.50 |
| 2010 | 26 | 2 | 0.50 | 0.50 |
| 2010 | 26 | 2 | 4.20 | 0.50 |
| 2010 | 26 | 2 | 0.50 | 0.50 |
| 2010 | 26 | 2 | 4.20 | 0.50 |
| 2010 | 26 | 2 | 2.70 | 0.50 |
| 2010 | 26 | 2 | 1.30 | 0.50 |
| 2010 | 27 | 1 | 8.70 | 0.00 |
| 2010 | 27 | 1 | 4.50 | 0.00 |
| 2010 | 27 | 1 | 5.10 | 0.00 |
| 2010 | 27 | 1 | 11.30 | 1.10 |
| 2010 | 27 | 1 | 5.20 | 1.50 |
| 2010 | 27 | 1 | 3.90 | 1.30 |
| 2010 | 27 | 1 | 11.70 | 0.90 |
| 2010 | 27 | 1 | 7.70 | 1.60 |
| 2010 | 27 | 1 | 10.60 | 2.30 |
| 2010 | 27 | 1 | 4.50 | 1.30 |
| 2010 | 27 | 2 | 4.30 | 3.10 |
| 2010 | 27 | 2 | 4.60 | 4.30 |
| 2010 | 27 | 2 | 4.90 | 6.20 |
| 2010 | 27 | 2 | 10.30 | 2.00 |

| | | | | |
|---|---|---|---|---|
| 2010 | 27 | 2 | 5.50 | 3.60 |
| 2010 | 27 | 2 | 6.10 | 0.50 |
| 2010 | 27 | 2 | 4.40 | 1.10 |
| 2010 | 27 | 2 | 8.90 | 0.90 |
| 2010 | 27 | 2 | 6.10 | 0.70 |
| 2010 | 27 | 2 | 5.30 | 1.40 |
| 2010 | 28 | 1 | 6.50 | 0.00 |
| 2010 | 28 | 1 | 5.40 | 3.90 |
| 2010 | 28 | 1 | 4.70 | 4.20 |
| 2010 | 28 | 1 | 4.60 | 3.40 |
| 2010 | 28 | 1 | 3.80 | 3.30 |
| 2010 | 28 | 1 | 6.10 | 4.20 |
| 2010 | 28 | 1 | 1.50 | 4.60 |
| 2010 | 28 | 1 | 6.50 | 6.40 |
| 2010 | 28 | 1 | 3.40 | 5.80 |
| 2010 | 28 | 1 | 5.20 | 2.30 |
| 2010 | 28 | 2 | 5.30 | 4.20 |
| 2010 | 28 | 2 | 5.90 | 3.90 |
| 2010 | 28 | 2 | 0.50 | 4.40 |
| 2010 | 28 | 2 | 3.60 | 3.50 |
| 2010 | 28 | 2 | 5.80 | 3.80 |
| 2010 | 28 | 2 | 8.30 | 1.90 |
| 2010 | 28 | 2 | 6.60 | 4.00 |
| 2010 | 28 | 2 | 4.20 | 3.00 |
| 2010 | 28 | 2 | 3.10 | 4.20 |
| 2010 | 28 | 2 | 4.20 | 3.70 |
| 2010 | 29 | 1 | 0.00 | 0.00 |
| 2010 | 29 | 1 | 2.20 | 0.00 |
| 2010 | 29 | 1 | 2.40 | 6.20 |
| 2010 | 29 | 1 | 2.20 | 4.30 |
| 2010 | 29 | 1 | 2.30 | 2.90 |
| 2010 | 29 | 1 | 0.50 | 2.80 |
| 2010 | 29 | 1 | 4.50 | 2.80 |
| 2010 | 29 | 1 | 0.50 | 11.20 |
| 2010 | 29 | 1 | 0.50 | 7.40 |
| 2010 | 29 | 1 | 2.80 | 3.70 |
| 2010 | 29 | 2 | 0.00 | 0.00 |
| 2010 | 29 | 2 | 3.20 | 0.50 |
| 2010 | 29 | 2 | 6.80 | 0.50 |
| 2010 | 29 | 2 | 2.70 | 1.50 |
| 2010 | 29 | 2 | 5.90 | 0.60 |
| 2010 | 29 | 2 | 4.60 | 1.70 |
| 2010 | 29 | 2 | 4.70 | 3.40 |
| 2010 | 29 | 2 | 4.10 | 0.90 |
| 2010 | 29 | 2 | 4.30 | 0.50 |
| 2010 | 29 | 2 | 5.30 | 0.40 |
| 2010 | 30 | 1 | 3.80 | 2.90 |

| | | | | |
|---|---|---|---|---|
| 2010 | 30 | 1 | 6.70 | 3.60 |
| 2010 | 30 | 1 | 10.50 | 2.70 |
| 2010 | 30 | 1 | 10.10 | 0.50 |
| 2010 | 30 | 1 | 9.00 | 3.70 |
| 2010 | 30 | 1 | 7.90 | 6.90 |
| 2010 | 30 | 1 | 9.30 | 5.50 |
| 2010 | 30 | 1 | 6.90 | 9.60 |
| 2010 | 30 | 1 | 2.50 | 3.70 |
| 2010 | 30 | 1 | 5.00 | 4.50 |
| 2010 | 30 | 2 | 14.60 | 0.00 |
| 2010 | 30 | 2 | 17.20 | 4.40 |
| 2010 | 30 | 2 | 14.20 | 6.20 |
| 2010 | 30 | 2 | 17.40 | 5.20 |
| 2010 | 30 | 2 | 14.40 | 5.30 |
| 2010 | 30 | 2 | 14.70 | 5.70 |
| 2010 | 30 | 2 | 8.30 | 5.40 |
| 2010 | 30 | 2 | 3.00 | 10.10 |
| 2010 | 30 | 2 | 9.20 | 5.30 |
| 2010 | 30 | 2 | 0.50 | 7.20 |
| 2010 | 31 | 1 | 4.70 | 10.00 |
| 2010 | 31 | 1 | 10.20 | 6.00 |
| 2010 | 31 | 1 | 8.90 | 7.20 |
| 2010 | 31 | 1 | 3.50 | 9.50 |
| 2010 | 31 | 1 | 9.00 | 9.00 |
| 2010 | 31 | 1 | 8.50 | 8.40 |
| 2010 | 31 | 1 | 5.90 | 6.00 |
| 2010 | 31 | 1 | 7.80 | 2.50 |
| 2010 | 31 | 1 | 3.60 | 4.00 |
| 2010 | 31 | 1 | 6.00 | 5.00 |
| 2010 | 31 | 2 | 12.60 | 7.20 |
| 2010 | 31 | 2 | 12.70 | 6.10 |
| 2010 | 31 | 2 | 13.10 | 7.90 |
| 2010 | 31 | 2 | 9.30 | 4.30 |
| 2010 | 31 | 2 | 8.20 | 4.30 |
| 2010 | 31 | 2 | 9.90 | 5.10 |
| 2010 | 31 | 2 | 11.60 | 7.80 |
| 2010 | 31 | 2 | 8.40 | 4.60 |
| 2010 | 31 | 2 | 7.10 | 5.70 |
| 2010 | 31 | 2 | 7.40 | 4.80 |
| 2010 | 32 | 1 | 0.00 | 0.00 |
| 2010 | 32 | 1 | 0.00 | 0.00 |
| 2010 | 32 | 1 | 2.30 | 0.00 |
| 2010 | 32 | 1 | 3.50 | 0.00 |
| 2010 | 32 | 1 | 8.70 | 0.00 |
| 2010 | 32 | 1 | 4.50 | 0.00 |
| 2010 | 32 | 1 | 3.20 | 4.20 |
| 2010 | 32 | 1 | 3.50 | 2.90 |

| | | | | |
|---|---|---|---|---|
| 2010 | 32 | 1 | 5.10 | 2.20 |
| 2010 | 32 | 1 | 4.30 | 2.10 |
| 2010 | 32 | 2 | 3.90 | 0.00 |
| 2010 | 32 | 2 | 2.80 | 0.50 |
| 2010 | 32 | 2 | 3.60 | 0.50 |
| 2010 | 32 | 2 | 1.90 | 0.50 |
| 2010 | 32 | 2 | 4.90 | 0.50 |
| 2010 | 32 | 2 | 10.80 | 0.50 |
| 2010 | 32 | 2 | 2.60 | 0.50 |
| 2010 | 32 | 2 | 3.80 | 0.50 |
| 2010 | 32 | 2 | 3.40 | 0.50 |
| 2010 | 32 | 2 | 1.40 | 0.50 |
| 2010 | 34 | 1 | 14.30 | 5.50 |
| 2010 | 34 | 1 | 7.90 | 4.40 |
| 2010 | 34 | 1 | 11.10 | 3.60 |
| 2010 | 34 | 1 | 4.60 | 7.90 |
| 2010 | 34 | 1 | 11.30 | 5.60 |
| 2010 | 34 | 1 | 12.50 | 5.60 |
| 2010 | 34 | 1 | 11.40 | 7.50 |
| 2010 | 34 | 1 | 9.80 | 2.50 |
| 2010 | 34 | 1 | 13.30 | 9.50 |
| 2010 | 34 | 1 | 5.50 | 5.50 |
| 2010 | 34 | 2 | 10.20 | 11.00 |
| 2010 | 34 | 2 | 9.50 | 6.00 |
| 2010 | 34 | 2 | 9.70 | 4.20 |
| 2010 | 34 | 2 | 16.60 | 8.90 |
| 2010 | 34 | 2 | 13.70 | 5.80 |
| 2010 | 34 | 2 | 17.80 | 10.10 |
| 2010 | 34 | 2 | 19.90 | 7.30 |
| 2010 | 34 | 2 | 13.60 | 11.50 |
| 2010 | 34 | 2 | 18.00 | 10.50 |
| 2010 | 34 | 2 | 27.20 | 3.00 |
| 2010 | 35 | 1 | 0.00 | 4.90 |
| 2010 | 35 | 1 | 4.50 | 5.60 |
| 2010 | 35 | 1 | 2.70 | 5.20 |
| 2010 | 35 | 1 | 5.00 | 5.50 |
| 2010 | 35 | 1 | 6.80 | 4.00 |
| 2010 | 35 | 1 | 5.40 | 6.50 |
| 2010 | 35 | 1 | 3.90 | 5.50 |
| 2010 | 35 | 1 | 2.50 | 2.70 |
| 2010 | 35 | 1 | 2.50 | 1.50 |
| 2010 | 35 | 1 | 4.10 | 9.40 |
| 2010 | 35 | 2 | 21.50 | 4.20 |
| 2010 | 35 | 2 | 12.30 | 5.70 |
| 2010 | 35 | 2 | 5.60 | 3.60 |
| 2010 | 35 | 2 | 10.30 | 7.70 |
| 2010 | 35 | 2 | 6.40 | 7.20 |

| | | | | |
|---|---|---|---|---|
| 2010 | 35 | 2 | 7.30 | 7.10 |
| 2010 | 35 | 2 | 14.60 | 13.10 |
| 2010 | 35 | 2 | 11.10 | 8.40 |
| 2010 | 35 | 2 | 10.20 | 9.40 |
| 2010 | 35 | 2 | 9.60 | 10.90 |
| 2010 | 36 | 1 | 7.20 | 8.30 |
| 2010 | 36 | 1 | 9.50 | 5.50 |
| 2010 | 36 | 1 | 9.00 | 6.00 |
| 2010 | 36 | 1 | 13.60 | 6.20 |
| 2010 | 36 | 1 | 11.20 | 11.00 |
| 2010 | 36 | 1 | 15.50 | 10.10 |
| 2010 | 36 | 1 | 16.50 | 6.20 |
| 2010 | 36 | 1 | 11.60 | 6.90 |
| 2010 | 36 | 1 | 11.90 | 0.50 |
| 2010 | 36 | 1 | 13.20 | 11.00 |
| 2010 | 36 | 2 | 0.00 | 14.60 |
| 2010 | 36 | 2 | 0.00 | 13.80 |
| 2010 | 36 | 2 | 18.40 | 3.50 |
| 2010 | 36 | 2 | 19.20 | 6.10 |
| 2010 | 36 | 2 | 10.60 | 5.50 |
| 2010 | 36 | 2 | 21.50 | 13.30 |
| 2010 | 36 | 2 | 19.90 | 12.40 |
| 2010 | 36 | 2 | 13.60 | 12.90 |
| 2010 | 36 | 2 | 11.40 | 13.20 |
| 2010 | 36 | 2 | 4.90 | 8.20 |
| 2022 | 42 | 1 | 12.30 | 0.00 |
| 2022 | 42 | 1 | 14.50 | 0.00 |
| 2022 | 42 | 1 | 15.50 | 0.00 |
| 2022 | 42 | 1 | 13.20 | 0.00 |
| 2022 | 42 | 1 | 10.70 | 0.00 |
| 2022 | 42 | 1 | 10.40 | 0.50 |
| 2022 | 42 | 1 | 0.00 | 0.50 |
| 2022 | 42 | 1 | 0.00 | 7.90 |
| 2022 | 42 | 1 | 0.00 | 8.90 |
| 2022 | 42 | 1 | 0.00 | 4.60 |
| 2022 | 42 | 2 | 0.00 | 0.00 |
| 2022 | 42 | 2 | 0.00 | 0.00 |
| 2022 | 42 | 2 | 0.00 | 0.00 |
| 2022 | 42 | 2 | 0.00 | 0.00 |
| 2022 | 42 | 2 | 25.00 | 6.30 |
| 2022 | 42 | 2 | 20.20 | 5.50 |
| 2022 | 42 | 2 | 9.80 | 3.90 |
| 2022 | 42 | 2 | 13.30 | 4.60 |
| 2022 | 42 | 2 | 10.70 | 2.20 |
| 2022 | 42 | 2 | 8.90 | 2.20 |
| 2022 | 43 | 1 | 6.00 | 0.00 |
| 2022 | 43 | 1 | 7.00 | 0.00 |

| | | | | |
|---|---|---|---|---|
| 2022 | 43 | 1 | 7.50 | 0.00 |
| 2022 | 43 | 1 | 5.00 | 0.00 |
| 2022 | 43 | 1 | 0.00 | 0.00 |
| 2022 | 43 | 1 | 0.00 | 0.00 |
| 2022 | 43 | 1 | 0.00 | 0.00 |
| 2022 | 43 | 1 | 0.00 | 0.00 |
| 2022 | 43 | 1 | 0.00 | 0.00 |
| 2022 | 43 | 1 | 0.00 | 0.00 |
| 2022 | 43 | 2 | 0.00 | 0.00 |
| 2022 | 43 | 2 | 0.00 | 0.00 |
| 2022 | 43 | 2 | 0.00 | 0.00 |
| 2022 | 43 | 2 | 6.50 | 0.00 |
| 2022 | 43 | 2 | 12.00 | 4.00 |
| 2022 | 43 | 2 | 10.00 | 2.00 |
| 2022 | 43 | 2 | 19.00 | 13.00 |
| 2022 | 43 | 2 | 6.00 | 5.00 |
| 2022 | 43 | 2 | 13.00 | 5.50 |
| 2022 | 43 | 2 | 6.50 | 13.00 |
| 2022 | 44 | 1 | 22.30 | 0.00 |
| 2022 | 44 | 1 | 14.50 | 0.00 |
| 2022 | 44 | 1 | 14.30 | 0.00 |
| 2022 | 44 | 1 | 25.20 | 0.00 |
| 2022 | 44 | 1 | 18.30 | 21.50 |
| 2022 | 44 | 1 | 7.50 | 14.50 |
| 2022 | 44 | 1 | 17.80 | 16.20 |
| 2022 | 44 | 1 | 0.00 | 0.00 |
| 2022 | 44 | 1 | 0.00 | 0.00 |
| 2022 | 44 | 1 | 0.00 | 0.00 |
| 2022 | 44 | 2 | 0.00 | 0.00 |
| 2022 | 44 | 2 | 0.00 | 0.00 |
| 2022 | 44 | 2 | 12.30 | 0.00 |
| 2022 | 44 | 2 | 23.70 | 0.00 |
| 2022 | 44 | 2 | 19.60 | 0.00 |
| 2022 | 44 | 2 | 21.00 | 6.50 |
| 2022 | 44 | 2 | 14.90 | 2.70 |
| 2022 | 44 | 2 | 2.50 | 13.50 |
| 2022 | 44 | 2 | 4.50 | 14.30 |
| 2022 | 44 | 2 | 16.40 | 12.50 |
| 2022 | 45 | 1 | 3.50 | 5.20 |
| 2022 | 45 | 1 | 7.60 | 10.00 |
| 2022 | 45 | 1 | 8.10 | 8.50 |
| 2022 | 45 | 1 | 5.40 | 7.00 |
| 2022 | 45 | 1 | 8.60 | 8.10 |
| 2022 | 45 | 1 | 5.50 | 1.50 |
| 2022 | 45 | 1 | 7.60 | 3.00 |
| 2022 | 45 | 1 | 12.30 | 6.10 |
| 2022 | 45 | 1 | 10.10 | 8.30 |

| | | | | |
|---|---|---|---|---|
| 2022 | 45 | 1 | 6.70 | 0.50 |
| 2022 | 45 | 2 | 0.00 | 5.50 |
| 2022 | 45 | 2 | 0.00 | 8.00 |
| 2022 | 45 | 2 | 4.50 | 3.20 |
| 2022 | 45 | 2 | 6.00 | 6.00 |
| 2022 | 45 | 2 | 11.50 | 5.50 |
| 2022 | 45 | 2 | 5.90 | 3.20 |
| 2022 | 45 | 2 | 0.50 | 2.00 |
| 2022 | 45 | 2 | 5.50 | 3.00 |
| 2022 | 45 | 2 | 10.50 | 4.20 |
| 2022 | 45 | 2 | 5.60 | 8.70 |
| 2022 | 47 | 1 | 10.80 | 0.50 |
| 2022 | 47 | 1 | 11.30 | 0.50 |
| 2022 | 47 | 1 | 1.50 | 0.50 |
| 2022 | 47 | 1 | 0.50 | 4.00 |
| 2022 | 47 | 1 | 3.50 | 0.50 |
| 2022 | 47 | 1 | 5.70 | 0.50 |
| 2022 | 47 | 1 | 5.50 | 1.50 |
| 2022 | 47 | 1 | 3.50 | 3.50 |
| 2022 | 47 | 1 | 3.70 | 4.60 |
| 2022 | 47 | 1 | 4.00 | 5.70 |
| 2022 | 47 | 2 | 5.50 | 0.50 |
| 2022 | 47 | 2 | 3.50 | 0.50 |
| 2022 | 47 | 2 | 6.70 | 0.50 |
| 2022 | 47 | 2 | 6.90 | 3.50 |
| 2022 | 47 | 2 | 5.80 | 4.20 |
| 2022 | 47 | 2 | 8.55 | 9.70 |
| 2022 | 47 | 2 | 0.50 | 4.80 |
| 2022 | 47 | 2 | 3.50 | 9.50 |
| 2022 | 47 | 2 | 3.70 | 16.20 |
| 2022 | 47 | 2 | 4.50 | 0.50 |
| 2022 | 48 | 1 | 0.00 | 0.00 |
| 2022 | 48 | 1 | 0.00 | 0.00 |
| 2022 | 48 | 1 | 0.00 | 0.00 |
| 2022 | 48 | 1 | 0.00 | 0.00 |
| 2022 | 48 | 1 | 0.00 | 0.00 |
| 2022 | 48 | 1 | 0.00 | 0.00 |
| 2022 | 48 | 1 | 0.00 | 0.00 |
| 2022 | 48 | 1 | 0.00 | 6.00 |
| 2022 | 48 | 1 | 0.00 | 2.00 |
| 2022 | 48 | 1 | 5.00 | 2.50 |
| 2022 | 48 | 2 | 0.00 | 0.00 |
| 2022 | 48 | 2 | 0.00 | 0.00 |
| 2022 | 48 | 2 | 0.00 | 0.00 |
| 2022 | 48 | 2 | 0.00 | 0.00 |
| 2022 | 48 | 2 | 0.00 | 0.00 |
| 2022 | 48 | 2 | 3.00 | 0.00 |

| | | | | |
|---|---|---|---|---|
| 2022 | 48 | 2 | 13.00 | 0.00 |
| 2022 | 48 | 2 | 11.00 | 1.50 |
| 2022 | 48 | 2 | 13.00 | 4.50 |
| 2022 | 48 | 2 | 12.00 | 6.00 |
| 2022 | 49 | 1 | 12.30 | 2.50 |
| 2022 | 49 | 1 | 2.50 | 2.50 |
| 2022 | 49 | 1 | 3.50 | 2.50 |
| 2022 | 49 | 1 | 4.60 | 3.70 |
| 2022 | 49 | 1 | 4.30 | 4.20 |
| 2022 | 49 | 1 | 6.30 | 5.40 |
| 2022 | 49 | 1 | 2.50 | 4.50 |
| 2022 | 49 | 1 | 2.40 | 1.50 |
| 2022 | 49 | 1 | 1.80 | 3.00 |
| 2022 | 49 | 1 | 2.30 | 2.50 |
| 2022 | 49 | 2 | 0.00 | 0.50 |
| 2022 | 49 | 2 | 0.00 | 0.50 |
| 2022 | 49 | 2 | 1.50 | 0.50 |
| 2022 | 49 | 2 | 2.50 | 1.50 |
| 2022 | 49 | 2 | 1.50 | 4.00 |
| 2022 | 49 | 2 | 2.70 | 3.60 |
| 2022 | 49 | 2 | 5.40 | 1.50 |
| 2022 | 49 | 2 | 0.50 | 2.00 |
| 2022 | 49 | 2 | 2.60 | 0.50 |
| 2022 | 49 | 2 | 0.50 | 0.00 |
| 2022 | 50 | 1 | 0.00 | 4.40 |
| 2022 | 50 | 1 | 0.00 | 0.50 |
| 2022 | 50 | 1 | 0.00 | 0.00 |
| 2022 | 50 | 1 | 0.00 | 0.00 |
| 2022 | 50 | 1 | 5.60 | 0.00 |
| 2022 | 50 | 1 | 11.20 | 0.00 |
| 2022 | 50 | 1 | 16.20 | 0.00 |
| 2022 | 50 | 1 | 0.50 | 0.00 |
| 2022 | 50 | 1 | 4.50 | 0.00 |
| 2022 | 50 | 1 | 13.20 | 0.00 |
| 2022 | 50 | 2 | 21.50 | 4.90 |
| 2022 | 50 | 2 | 8.50 | 9.50 |
| 2022 | 50 | 2 | 0.00 | 0.00 |
| 2022 | 50 | 2 | 0.00 | 0.00 |
| 2022 | 50 | 2 | 0.00 | 0.00 |
| 2022 | 50 | 2 | 0.00 | 0.00 |
| 2022 | 50 | 2 | 0.00 | 0.00 |
| 2022 | 50 | 2 | 0.00 | 0.00 |
| 2022 | 50 | 2 | 0.00 | 0.00 |
| 2022 | 50 | 2 | 0.00 | 0.00 |
| 2022 | 52 | 1 | 9.50 | 0.50 |
| 2022 | 52 | 1 | 10.20 | 1.50 |
| 2022 | 52 | 1 | 15.20 | 3.50 |

| | | | | |
|---|---|---|---|---|
| 2022 | 52 | 1 | 1.50 | 8.10 |
| 2022 | 52 | 1 | 7.40 | 10.00 |
| 2022 | 52 | 1 | 4.20 | 9.00 |
| 2022 | 52 | 1 | 11.60 | 4.50 |
| 2022 | 52 | 1 | 10.30 | 5.00 |
| 2022 | 52 | 1 | 7.40 | 9.20 |
| 2022 | 52 | 1 | 6.20 | 5.60 |
| 2022 | 52 | 2 | 0.00 | 9.50 |
| 2022 | 52 | 2 | 11.50 | 7.90 |
| 2022 | 52 | 2 | 10.30 | 7.40 |
| 2022 | 52 | 2 | 9.50 | 10.00 |
| 2022 | 52 | 2 | 8.20 | 6.20 |
| 2022 | 52 | 2 | 7.40 | 7.30 |
| 2022 | 52 | 2 | 6.50 | 6.50 |
| 2022 | 52 | 2 | 0.50 | 6.70 |
| 2022 | 52 | 2 | 2.50 | 2.90 |
| 2022 | 52 | 2 | 5.70 | 0.50 |
| 2022 | 53 | 1 | 0.00 | 0.50 |
| 2022 | 53 | 1 | 7.50 | 5.60 |
| 2022 | 53 | 1 | 8.20 | 3.50 |
| 2022 | 53 | 1 | 9.40 | 4.50 |
| 2022 | 53 | 1 | 0.50 | 6.40 |
| 2022 | 53 | 1 | 11.50 | 3.60 |
| 2022 | 53 | 1 | 12.10 | 7.00 |
| 2022 | 53 | 1 | 9.50 | 4.30 |
| 2022 | 53 | 1 | 8.40 | 5.20 |
| 2022 | 53 | 1 | 7.50 | 4.00 |
| 2022 | 53 | 2 | 2.50 | 0.00 |
| 2022 | 53 | 2 | 8.10 | 0.00 |
| 2022 | 53 | 2 | 4.50 | 2.40 |
| 2022 | 53 | 2 | 0.50 | 5.60 |
| 2022 | 53 | 2 | 0.50 | 5.50 |
| 2022 | 53 | 2 | 7.60 | 6.00 |
| 2022 | 53 | 2 | 8.50 | 10.50 |
| 2022 | 53 | 2 | 9.20 | 3.00 |
| 2022 | 53 | 2 | 0.00 | 4.90 |
| 2022 | 53 | 2 | 0.00 | 0.50 |
| 2022 | 54 | 1 | 0.00 | 0.00 |
| 2022 | 54 | 1 | 0.00 | 10.00 |
| 2022 | 54 | 1 | 0.00 | 14.00 |
| 2022 | 54 | 1 | 0.00 | 15.00 |
| 2022 | 54 | 1 | 9.00 | 12.50 |
| 2022 | 54 | 1 | 17.50 | 12.00 |
| 2022 | 54 | 1 | 15.50 | 19.50 |
| 2022 | 54 | 1 | 16.00 | 15.00 |
| 2022 | 54 | 1 | 20.00 | 20.50 |
| 2022 | 54 | 1 | 11.00 | 18.00 |

| | | | | |
|---|---|---|---|---|
| 2022 | 54 | 2 | 0.00 | 0.00 |
| 2022 | 54 | 2 | 0.00 | 0.00 |
| 2022 | 54 | 2 | 18.00 | 0.00 |
| 2022 | 54 | 2 | 17.50 | 0.00 |
| 2022 | 54 | 2 | 21.00 | 0.00 |
| 2022 | 54 | 2 | 21.00 | 14.50 |
| 2022 | 54 | 2 | 17.50 | 12.00 |
| 2022 | 54 | 2 | 19.50 | 11.00 |
| 2022 | 54 | 2 | 20.50 | 17.00 |
| 2022 | 54 | 2 | 18.00 | 13.00 |
| 2022 | 56 | 1 | 3.00 | 0.00 |
| 2022 | 56 | 1 | 9.00 | 0.00 |
| 2022 | 56 | 1 | 0.00 | 0.00 |
| 2022 | 56 | 1 | 0.00 | 0.00 |
| 2022 | 56 | 1 | 0.00 | 0.00 |
| 2022 | 56 | 1 | 0.00 | 0.00 |
| 2022 | 56 | 1 | 0.00 | 0.00 |
| 2022 | 56 | 1 | 0.00 | 0.00 |
| 2022 | 56 | 1 | 0.00 | 0.00 |
| 2022 | 56 | 1 | 0.00 | 0.00 |
| 2022 | 56 | 2 | 16.00 | 12.00 |
| 2022 | 56 | 2 | 0.00 | 0.00 |
| 2022 | 56 | 2 | 0.00 | 0.00 |
| 2022 | 56 | 2 | 0.00 | 0.00 |
| 2022 | 56 | 2 | 0.00 | 0.00 |
| 2022 | 56 | 2 | 0.00 | 0.00 |
| 2022 | 56 | 2 | 0.00 | 0.00 |
| 2022 | 56 | 2 | 0.00 | 0.00 |
| 2022 | 56 | 2 | 0.00 | 0.00 |
| 2022 | 56 | 2 | 0.00 | 0.00 |
| 2022 | 57 | 1 | 0.50 | 0.00 |
| 2022 | 57 | 1 | 0.50 | 0.00 |
| 2022 | 57 | 1 | 0.50 | 0.00 |
| 2022 | 57 | 1 | 4.50 | 0.50 |
| 2022 | 57 | 1 | 6.30 | 0.50 |
| 2022 | 57 | 1 | 7.10 | 0.50 |
| 2022 | 57 | 1 | 2.50 | 1.50 |
| 2022 | 57 | 1 | 3.40 | 4.00 |
| 2022 | 57 | 1 | 5.40 | 3.50 |
| 2022 | 57 | 1 | 7.30 | 5.20 |
| 2022 | 57 | 2 | 0.00 | 0.00 |
| 2022 | 57 | 2 | 0.00 | 3.50 |
| 2022 | 57 | 2 | 6.50 | 5.20 |
| 2022 | 57 | 2 | 5.40 | 0.50 |
| 2022 | 57 | 2 | 3.50 | 0.40 |
| 2022 | 57 | 2 | 5.90 | 3.20 |
| 2022 | 57 | 2 | 1.50 | 5.40 |

| | | | | |
|---|---|---|---|---|
| 2022 | 57 | 2 | 1.50 | 2.90 |
| 2022 | 57 | 2 | 7.70 | 5.80 |
| 2022 | 57 | 2 | 4.20 | 8.00 |
| 2022 | 59 | 1 | 0.00 | 0.00 |
| 2022 | 59 | 1 | 0.00 | 0.00 |
| 2022 | 59 | 1 | 0.00 | 0.00 |
| 2022 | 59 | 1 | 0.00 | 0.00 |
| 2022 | 59 | 1 | 0.00 | 3.00 |
| 2022 | 59 | 1 | 17.00 | 15.00 |
| 2022 | 59 | 1 | 15.00 | 4.50 |
| 2022 | 59 | 1 | 3.50 | 10.00 |
| 2022 | 59 | 1 | 20.50 | 18.00 |
| 2022 | 59 | 1 | 14.50 | 20.00 |
| 2022 | 59 | 2 | 0.00 | 0.00 |
| 2022 | 59 | 2 | 0.00 | 0.00 |
| 2022 | 59 | 2 | 0.00 | 0.00 |
| 2022 | 59 | 2 | 0.00 | 0.00 |
| 2022 | 59 | 2 | 0.00 | 0.00 |
| 2022 | 59 | 2 | 4.50 | 0.00 |
| 2022 | 59 | 2 | 22.00 | 0.00 |
| 2022 | 59 | 2 | 20.50 | 5.00 |
| 2022 | 59 | 2 | 30.00 | 11.00 |
| 2022 | 59 | 2 | 22.50 | 10.00 |
| 2022 | 60 | 1 | 0.00 | 0.00 |
| 2022 | 60 | 1 | 4.50 | 0.00 |
| 2022 | 60 | 1 | 9.50 | 0.00 |
| 2022 | 60 | 1 | 6.20 | 4.00 |
| 2022 | 60 | 1 | 0.50 | 6.20 |
| 2022 | 60 | 1 | 0.50 | 8.60 |
| 2022 | 60 | 1 | 3.50 | 7.30 |
| 2022 | 60 | 1 | 4.70 | 2.50 |
| 2022 | 60 | 1 | 8.30 | 6.00 |
| 2022 | 60 | 1 | 5.10 | 1.50 |
| 2022 | 60 | 2 | 0.00 | 0.50 |
| 2022 | 60 | 2 | 0.00 | 0.50 |
| 2022 | 60 | 2 | 0.00 | 0.50 |
| 2022 | 60 | 2 | 0.00 | 0.50 |
| 2022 | 60 | 2 | 2.80 | 0.50 |
| 2022 | 60 | 2 | 0.50 | 7.00 |
| 2022 | 60 | 2 | 4.30 | 4.50 |
| 2022 | 60 | 2 | 5.60 | 3.00 |
| 2022 | 60 | 2 | 4.40 | 5.20 |
| 2022 | 60 | 2 | 6.30 | 3.00 |
| 2022 | 62 | 1 | 0.00 | 8.30 |
| 2022 | 62 | 1 | 0.00 | 7.50 |
| 2022 | 62 | 1 | 0.00 | 6.10 |
| 2022 | 62 | 1 | 0.50 | 5.20 |

| | | | | |
|---|---|---|---|---|
| 2022 | 62 | 1 | 11.80 | 4.00 |
| 2022 | 62 | 1 | 12.90 | 6.50 |
| 2022 | 62 | 1 | 5.60 | 0.00 |
| 2022 | 62 | 1 | 8.70 | 0.00 |
| 2022 | 62 | 1 | 7.60 | 0.00 |
| 2022 | 62 | 1 | 7.30 | 0.00 |
| 2022 | 62 | 2 | 0.00 | 0.00 |
| 2022 | 62 | 2 | 0.00 | 0.00 |
| 2022 | 62 | 2 | 10.60 | 0.00 |
| 2022 | 62 | 2 | 10.40 | 0.00 |
| 2022 | 62 | 2 | 14.70 | 0.00 |
| 2022 | 62 | 2 | 8.60 | 0.50 |
| 2022 | 62 | 2 | 8.90 | 7.10 |
| 2022 | 62 | 2 | 10.30 | 9.40 |
| 2022 | 62 | 2 | 4.30 | 5.10 |
| 2022 | 62 | 2 | 6.80 | 0.50 |

**Table S6b.** Phenotypic measures for 16 inbred lines/year tested. The inbred lines have a specific code; the length of coleoptile (cm) was determined as mean of ten inoculated kernels with *A. flavus* for each line in two replications. Values represent the mean of each line/replication.

| Year | Line Code | Replication | Length of coleoptile |
|---|---|---|---|
| 2001 | 1 | 1 | 0.89 |
| 2001 | 2 | 1 | 3.59 |
| 2001 | 4 | 1 | 1.67 |
| 2001 | 5 | 1 | 3.95 |
| 2001 | 6 | 1 | 2.08 |
| 2001 | 7 | 1 | 2.57 |
| 2001 | 8 | 1 | 1.02 |
| 2001 | 9 | 1 | 1.35 |
| 2001 | 10 | 1 | 1.57 |
| 2001 | 11 | 1 | 6.78 |
| 2001 | 12 | 1 | 4.40 |
| 2001 | 13 | 1 | 7.18 |
| 2001 | 14 | 1 | 5.53 |
| 2001 | 15 | 1 | 2.88 |
| 2001 | 16 | 1 | 1.15 |
| 2001 | 17 | 1 | 9.85 |
| 2010 | 20 | 1 | 6.12 |
| 2010 | 21 | 1 | 5.72 |
| 2010 | 22 | 1 | 1.51 |
| 2010 | 23 | 1 | 4.94 |
| 2010 | 24 | 1 | 3.95 |
| 2010 | 25 | 1 | 11.10 |
| 2010 | 26 | 1 | 1.56 |

| | | | |
|---|---|---|---|
| 2010 | 27 | 1 | 1.00 |
| 2010 | 28 | 1 | 3.81 |
| 2010 | 29 | 1 | 4.13 |
| 2010 | 30 | 1 | 4.36 |
| 2010 | 31 | 1 | 6.78 |
| 2010 | 32 | 1 | 1.14 |
| 2010 | 34 | 1 | 5.76 |
| 2010 | 35 | 1 | 5.08 |
| 2010 | 36 | 1 | 7.17 |
| 2022 | 42 | 1 | 2.24 |
| 2022 | 43 | 1 | 5.00 |
| 2022 | 44 | 1 | 8.61 |
| 2022 | 45 | 1 | 5.82 |
| 2022 | 47 | 1 | 2.18 |
| 2022 | 48 | 1 | 1.05 |
| 2022 | 49 | 1 | 3.23 |
| 2022 | 50 | 1 | 0.49 |
| 2022 | 52 | 1 | 5.69 |
| 2022 | 53 | 1 | 4.46 |
| 2022 | 54 | 1 | 13.65 |
| 2022 | 56 | 1 | 0.00 |
| 2022 | 57 | 1 | 1.57 |
| 2022 | 59 | 1 | 7.05 |
| 2022 | 60 | 1 | 3.61 |
| 2022 | 62 | 1 | 3.76 |
| 2001 | 1 | 2 | 2.15 |
| 2001 | 2 | 2 | 3.73 |
| 2001 | 4 | 2 | 2.21 |
| 2001 | 5 | 2 | 5.27 |
| 2001 | 6 | 2 | 1.70 |
| 2001 | 7 | 2 | 1.55 |
| 2001 | 8 | 2 | 2.62 |
| 2001 | 9 | 2 | 5.19 |
| 2001 | 10 | 2 | 1.56 |
| 2001 | 11 | 2 | 3.93 |
| 2001 | 12 | 2 | 2.14 |
| 2001 | 13 | 2 | 4.08 |
| 2001 | 14 | 2 | 2.03 |
| 2001 | 15 | 2 | 1.28 |
| 2001 | 16 | 2 | 0.18 |
| 2001 | 17 | 2 | 7.33 |
| 2010 | 20 | 2 | 4.44 |
| 2010 | 21 | 2 | 4.95 |
| 2010 | 22 | 2 | 0.71 |
| 2010 | 23 | 2 | 5.88 |

| | | | |
|---|---|---|---|
| 2010 | 24 | 2 | 4.16 |
| 2010 | 25 | 2 | 5.78 |
| 2010 | 26 | 2 | 0.52 |
| 2010 | 27 | 2 | 2.38 |
| 2010 | 28 | 2 | 3.66 |
| 2010 | 29 | 2 | 1.00 |
| 2010 | 30 | 2 | 5.48 |
| 2010 | 31 | 2 | 5.78 |
| 2010 | 32 | 2 | 0.45 |
| 2010 | 34 | 2 | 7.83 |
| 2010 | 35 | 2 | 7.73 |
| 2010 | 36 | 2 | 10.4 |
| 2022 | 42 | 2 | 2.47 |
| 2022 | 43 | 2 | 4.25 |
| 2022 | 44 | 2 | 4.95 |
| 2022 | 45 | 2 | 4.93 |
| 2022 | 47 | 2 | 4.99 |
| 2022 | 48 | 2 | 1.20 |
| 2022 | 49 | 2 | 1.46 |
| 2022 | 50 | 2 | 1.44 |
| 2022 | 52 | 2 | 6.49 |
| 2022 | 53 | 2 | 3.84 |
| 2022 | 54 | 2 | 6.75 |
| 2022 | 56 | 2 | 1.20 |
| 2022 | 57 | 2 | 3.49 |
| 2022 | 59 | 2 | 2.60 |
| 2022 | 60 | 2 | 2.52 |
| 2022 | 62 | 2 | 2.26 |

**Table S6c.** Statistical output (analysis of variance - ANOVA) of the phenotypic measures (length of coleoptile) for 16 inbred lines having Year as parameter. The normality of the dataset was tested prior to ANOVA using the Kolmogorov-Smirnov tests.

| Parameters | Degree of freedom | Sum Square | Mean Square | F value | Pr (>F) | p-value |
|---|---|---|---|---|---|---|
| Year | 2.0 | 348.34 | 174.17 | 9.39 | <0.0001 | *** |

p-value; no significant: ° p< 1, . p<0.1; significant: * p<0.05, ** p<0.01, *** p< 0.001

**Table S6d.** Statistical output (analysis of variance - ANOVA) of the phenotypic measures (length of coleoptile) for 16 inbred lines in 2001 having Treatment as parameter. The normality of the dataset was tested prior to ANOVA using the Kolmogorov-Smirnov tests.

| Parameters | Degree of freedom | Sum Square | Mean Square | F value | Pr (>F) | p-value |
|---|---|---|---|---|---|---|
| Treatment | 1.0 | 27.20 | 27.20 | 3.60 | 0.1 | (no significant) |

p-value; no significant: ° p< 1,. p<0.1; significant: * p<0.05, ** p<0.01, *** p< 0.001
